# Association between child and youth physical activity and family functioning: a systematic review of observational studies

**DOI:** 10.1186/s12966-025-01782-z

**Published:** 2025-07-22

**Authors:** Yasmine Forghani Soong, Heather Hollman, Ryan E. Rhodes

**Affiliations:** Behavioural Medicine Laboratory, School of Exercise Science, Physical and Health Education, University of Victoria, 3010, Victoria, BC V8W3N4 Canada

**Keywords:** Family functioning, Children, Youth, Physical activity

## Abstract

**Background:**

There are numerous psychosocial and health benefits linked to physical activity; however, 80% of children and youth are not meeting the recommended guidelines. Genetics, socio-economic status and familial factors impact childhood movement behaviors. In particular, active families support well developed and resilient offspring. As the family unit is optimally placed during a critical time in development, it is pertinent to explore the relationship between family functioning and child and youth physical activity. Purpose: To synthesize and analyze the literature to determine the associations between general domains of family functioning (general family functioning, cohesion, communication, conflict, organization, family problem-solving ability, household chaos, and affective environment) and child and youth (children: aged five to 12, youth: aged 13 to 17) physical activity.

**Methods:**

Literature searches across six databases were performed. Inclusion: Studies that performed and presented a statistical analysis between direct measurements of child and youth physical activity and general domains of family functioning. Exclusion: Indirect measurements of family functioning (e.g., support and encouragement). The summary median effect sizes (Pearson r) and interquartile range [IQR] were calculated between child or youth physical activity and each family functioning domain.

**Results:**

Search results k = 12,999. Included articles k = 43. Child physical activity had a small median effect size and indeterminate association with general family functioning (*r* = 0.09; [IQR]: -0.06 to 0.09) and cohesion (*r* = 0.06; [IQR]: 0.05 to 0.22). Youth physical activity presented with small median effect sizes and significant positive associations with the domains of general family functioning (r = 0.04; [IQR]: 0.02 to 0.06), cohesion (*r* = 0.09; [IQR]: 0.07 to 0.14), communication (*r* = 0.17; [IQR]: 0.09 to 0.40), and a negative association with the domain of conflict (*r* = -0.09; [IQR]: -0.21 to 0.02). Family problem-solving ability, organization, household chaos, and affective environment were understudied across both age groups.

**Conclusions:**

A small effect size in the domains of cohesion, communication, and conflict highlights the association between child and youth physical activity and family functioning. These findings provide a new avenue for researchers, programmers, and policy writers to target to support child and youth physical activity.

**Trial Registration:**

This review is registered with The National Institute for Health and Care Research at The International Prospective Register of Systematic Reviews (PROSPERO). PROSPERO ID number is CRD42023454220.

**Supplementary Information:**

The online version contains supplementary material available at 10.1186/s12966-025-01782-z.

## Background

It is widely acknowledged that regular engagement in physical activity provides many health benefits in children and youth [1–3], ranging from protective factors against chronic health conditions [3], development of muscles and bone mineral density [4] to positive effects on working memory and cognitive life skills [5]. As a result, the World Health Organization recommends that children and youth (aged five to 17 years) should accumulate at least 60 minutes of moderate- to vigorous-intensity physical activity each day [6]. Despite the positive health benefits, few children and youth worldwide are meeting these public health recommendations. For example, global estimates of physical inactivity indicated that more than three quarters of children and youth did not meet the recommended physical activity guidelines [7]. Thus, understanding the factors associated with child and youth physical activity is critical to improve promotion efforts, tailor interventions, and inform future research.

Research supports child and youth physical activity as resulting from a broad socio-ecological collection of factors, from individual and biological to policy and the built environment [8–10]. Family-level beliefs and behaviors shape children’s movement behaviors and ultimately influence their health status and quality of life [11]. Movement behaviors established during childhood and adolescence are known to track into adulthood and have been linked to long-term health, environmental, and economic outcomes [12]. While individual factors such as genetics, biological predispositions, and access to resources play important roles in shaping activity behaviors [13], the social environments, particularly the family environment, is influential in shaping children’s health-related behaviors and quality of life [11, 14–16]. While many parents acknowledge the positive effects of physical activity for their child, parental support and child and youth engagement are less than adequate [17–19]. Thus, to ensure the healthy growth and development in children and youth, it is imperative to explore areas within the family unit which can be leveraged to support regular engagement in physical activity [20].

Family systems theory highlights that families exist in a constant cycle of interactions, growth, and change, with evolution and problems being intrinsic to family life [21, 22]. In evaluating the behavior of a family member for intervention, it is crucial to take into account that recurring problems, reorganization, and adjustment are the normal components of the family life cycle [22, 23]. Therefore, when changing or managing a child's behavior it is important to examine not only the development of the child but also their place within the broader family system [23]. The family systems model is widely accepted and used in mental illness and behavioral treatment [23–25]. Given the complex, dynamic interplay of factors within a family, it is essential to break down the family unit into it’s domains of functioning, as each is constantly interacting and influencing one another and health behaviors, such as physical activity.

Family functioning encompasses the internal dynamics of the family unit and extends across five domains: cohesion (emotional bonding between family members), communication (the ability to effectively convey information and emotions), problem-solving ability (the ability to quickly resolve and work through conflict), affective environment (the emotional environment created by the family unit), and organization (the maintenance of the roles and responsibilities within the family unit) [26]. Family systems theory suggests that inadequate family functioning (e.g., minimal communication, limited affection, and/or inconsistent enforcement of rules/structure) may predispose a child to engage in unhealthy behaviors [27]. Thus, a family with adequate functioning could support their child to be more physically active, and vice versa [19]. For example, families with increased levels of cohesion could be more inclined to motivate their children or support their children to engage in sports, outdoor play, or other areas of physical activity. Furthermore, families with better communication may ensure that their child feels comfortable discussing physical activity interests or preferences (i.e., if a child feels more comfortable disclosing an interest in a certain sport), which in turn will likely result in them being more inclined to continue participating [28]. Similarly, families that are organized are likely able to provide consistent routines and support systems that facilitate regular physical activity [28]; whether this be to ensure that their child is transported to and from their sporting events, to planning for time to take their child out to the park.

While an adequately functioning family can support a child’s physical activity in many ways, there is also a reciprocal relationship, as a child who engages in more physical activity could also support a better-functioning family [29]. For example, children who engage in more physical activity have lower levels of stress [30]. Reduced levels of stress, in turn, allow for a child to be more receptive to bonding with parents (versus self-isolating), and ease the potential for tension or conflict in the family unit [31]. Indeed, it has been demonstrated that physical activity supports the development of communication skills in children, further facilitating a family’s ability to communicate with each other [32].

Furthermore, public health researchers have highlighted the importance of utilizing a family systems approach when designing health behavior interventions for children, particularly interventions to promote physical activity [33–35]. For instance, research suggests that targeting areas like family cohesion or communication can enhance overall family functioning and lead to more successful interventions [27, 36]. Emphasizing these domains in policy design could foster more holistic approaches to child and youth health interventions, particularly in promoting physical activity. Given the ever-diversifying climate of our population, this perspective could guide more effective, family-centered policy development that supports sustainable relevant changes in children’s health behaviors and overall well-being.

While current literature has summarized specific measures of family functioning as they relate to child and youth physical activity, such as familial support [37], or family engagement [38], there is a gap in the literature synthesizing observational studies exploring the association between general domains of family functioning and child and youth physical activity. Observational studies allow researchers to study children and families in their everyday environment without manipulation or interference [39]. They also allow for the potential sampling of a larger often much more heterogenous population than intervention or clinical case studies. Racialized minorities, such as individuals of Hispanic or African American ethnicity are often underrepresented during intervention trials [40]. A broader sampling range of observational studies can allow for a much more representative sample, potentially mitigating underrepresentation [39]. This is particularly important as these populations are often at a higher risk of developing chronic diseases such as type II diabetes or cardiovascular disease, which are both preventable with adequate physical activity [41]. Further, the current literature has been systematically reviewed for the impacts of family-based physical activity interventions on family functioning [29], how family-based interventions can increase physical activity in children [42], and the relationship between child and adolescent obesity and family functioning [43]. Not only that, but a growing body of observational research identifies parental and familial influence as consistent correlates of child and youth physical activity behaviors [11, 27, 36]. Thus, a complementary step is to synthesize observational studies that have explored the association between child and youth physical activity and family functioning.

As such, the purpose of this review is to synthesize observational study findings on the association between family functioning and child and youth physical activity. We believe that a focus on this information will prove beneficial to help inform future interventions on which family function domains are associated with child and youth physical activity, thus assisting in targeting policies and programs to promote increased physical activity in children and youth, all with the overall goal of working toward healthier family dynamics.

## Methods

To ensure transparency and comprehensive reporting, this review was conducted following the preferred reporting items for systematic reviews and meta-analyses [44] and further tailored using guidelines for conducting systematic reviews and meta-analyses of observational studies of etiology [45]. The proposed study was registered on PROSPERO on August 8th, 2023 (ID: CRD42023454220).

### Eligibility criteria

The included population was defined as children and youth (ages five to 17 years) and their family unit (e.g., parent-child, mother-child, father-child, guardian-child, grandparent-child). The age range for children and youth was based off the recommendations of the World Health Organization physical activity and sedentary behavior guidelines for children and adolescents, and the Canadian physical activity guidelines for children and youth. Both these organizations categorize children and youth as those between the ages of five to 17 years [6, 46]. Clinical populations (e.g., diabetic, liver transplant, etc.) were considered eligible for review. All forms of observational, non-experimental quantitative studies (e.g., cross-sectional, longitudinal, etc.) presenting an association between child physical activity and general family functioning domains (cohesion, organization, communication, affective environment, problem-solving ability, and household chaos) were included [29]. Child physical activity, defined as any sort of voluntary bodily movement that is not sedentary behavior, and is measured and recorded within the study was eligible for inclusion [47]. As there is a wide range of modalities, synonyms, keywords, and headings for physical activity, the physical activity search concept was based on an article that surveyed Canadian children to determine commonly engaged modes of physical activity [48]. This ensured the physical activity search concept was relevant and applicable to the current review. Finally, only studies that reported a test of association between family functioning and child and youth physical activity were deemed eligible for inclusion.

### Exclusion criteria

Indirect measures of family functioning (e.g., family support, encouragement, and general attitudes surrounding physical activity) were not included in the current study. Furthermore, studies observing child or youth athletes were only included if a direct measure of physical activity was recorded and reported during the study period.

### Search strategy development

#### Six databases

Medline (OVID), APA PsycINFO (EBSCO), SPORTDiscus (EBSCO), Web of Science Core Collection (Web of Science), Scopus (Elsevier), and CINAHL (EBSCO) were searched by the research team. All databases were set to include theses and dissertations. To further expand the scope of the grey literature search Scopus and Web of Science were configured to include theses/dissertations, conference proceedings, and book chapters. The search strategy (Appendix A) was developed by YFS with help from librarian ZP at the University of Victoria and reviewed by HH and RER.

### Study screening and selection

#### Title and abstract screening

Similar to another review in the field of physical activity [49], an artificial intelligence (AI) screening tool, ASReview, was implemented to assist with title and abstract screening. ASReview is a systematic review screening tool that is trained to rearrange the included texts, by placing the more relevant pieces at the beginning of the queue, and the less relevant ones at the back. This allows for a more efficient screening, both in terms of reduced time, but also because human screening can be prone to error [50, 51].

Adhering to ASReview screening platform setup instructions [52], YFS trained the AI using articles from the uploaded set of texts, with seven relevant seed papers, and seven randomly picked irrelevant articles. In addition to training the AI, the following settings were applied to the screening platform: the mode of screening was set to “Oracle”, allowing for review of the dataset with an interactive artificial intelligence; the model followed the default setup which included Term Frequency-Inverse Document Frequency (TF-IDF) for feature extraction technique; Naïve Bayes for classifier; maximum for query strategy; and dynamic resampling (double) for the balance strategy. This setup allowed for implementation of ASReview’s active learning system to label records [52].

Based on suggested ASReview screening processes, a data-driven stop criterion was adopted [52]. A data-driven screening strategy can achieve 95% sensitivity, making it a practical stopping criterion [53]. Based on ASReview platform recommendation [52], and previously conducted research [49], a stop criterion of 50 back-to-back irrelevant studies was agreed upon by all authors.

#### Full text screening

Upon screening 50 back-to-back irrelevant studies on the ASReview platform, YFS uploaded the included full text records to the systematic review data extraction and screening platform, Covidence [54]. YFS and HH screened all full text records. Conflicts between screeners were sent to RER for resolution.

### Data extraction

Data extraction was conducted by YFS and reviewed by RER and HH. Following the Preferred Reporting Items for Systematic Reviews and Meta-Analyses (PRISMA) guidelines the following areas of each study were extracted: Title of article, author(s), year of publication, country, sample population, family unit description, study design, study methods, if the data was obtained via a subset of a prior study cohort, physical activity measure, physical activity results, family functioning measure, report of family functioning domain, and results of association between physical activity and domain(s) of family functioning.

### Quality assessment

In a review of quality assessment tools for human observational studies, nine key domains (selection, exposure, outcome assessment, confounding, loss to follow-up, analysis, selective reporting, conflicts of interest, and other) were found to be important for quality assessment tools to address to ensure clear and transparent reporting [55]. The Appraisal Tool for Cross-Sectional Studies (AXIS) was chosen as it covers five of the nine domains and was cited as a commonly used and recommended tool for risk of bias assessment in systematic reviews of observational studies [56]. YFS and HH independently performed the risk of bias assessment of the included studies. When finished screening YFS re-checked areas of conflict and resolved them through discussion with HH. Each domain provided in the AXIS tool had a substantial agreement to perfect agreement between raters [57]. The kappa scores are as follows: introduction a score of 1, methods a score of 0.81, results a score of 0.77, discussion a score of 1, and other a score of 0.71. The quality scoring can be found in Table 1.

**Table 1:** Observed agreement and Cohen’s Kappa for risk of bias assessment

		Reviewer A (Yes)	Reviewer A (No)	Reviewer B (Yes)		Reviewer B (No)			Cohen’s Kappa
Introduction									
Question 1		43	0	43		0			1
Total for Section									1
Methods									
Question 2		43	0	43		0			1
Question 3		15	28	11		32			0.78
Question 4		43	1	41		2			0.66
Question 5		42	1	40		3			0.48
Question 6		42	1	42		1			1
Question 7		16	27	10		33			0.68
Question 8		35	8	34		9			0.93
Question 9		37	6	35		7			0.72
Question 10		42	1	42		2			1
Question 11		41	3	39		4			0.82
Total for Section									0.81
Results									
Question 12		41	2	40		3			0.79
Question 13		25	18	24		19			0.57
Question 14		16	27	17		26			0.66
Question 15		40	3	39		4			0.84
Question 16		42	1	42		1			1
Total for Section									0.77
Discussion									
Question 17		43	0	43		0			1
Question 18		42	1	42		1			1
Total for Section									1
Other									
Question 19		22	21	26		27			0.60
Question 20		32	11	35		8			0.81
Total for Section									0.71

### Analysis

Following the initial read-throughs of the final included studies, considerable heterogeneity in measurement, design, and sampling was found. Thus, applying suggestions for synthesizing literature [58], a meta-analysis of the data was deemed inappropriate, and an alternative synthesis method was taken [59]. Following recommendations from the Cochrane Handbook for Systematic Reviews, summarizing effect estimates was deemed to be the most acceptable alternative method and was applied as an analysis approach. The authors categorized the findings based on family functioning outcome (general family functioning, cohesion, communication, conflict, organization, household chaos, affective environment, and family problem-solving ability), and sub-categorized based on child age (children five to twelve years, and youth 13–17years) [26]. This sub-categorization was based on the significant contrast between children and youth characterized by unique developmental needs, cognitive abilities, and emotional experiences [60]. Furthermore, this sub-categorization has been used in a previous review [29].

#### Summarizing effect estimates

To execute thematic classification, effect sizes, and significance values directly measuring the relationship between family functioning and child physical activity were extracted [61]. Median effect sizes and the interquartile ranges were used to summarize effect estimates [59]. As several studies presented multiple effect sizes the authors established a hierarchy of effect sizes. Firstly, as the authors were most interested in a bidirectional relationship, the Pearson correlation coefficient (*r*) was prioritized for median and interquartile range calculations. Secondly, for studies that presented multiple effect sizes based on different types/measurement devices of physical activity, moderate to vigorous measures of physical activity, and device-measured measurement (i.e., accelerometry) were prioritized. Moderate to vigorous physical activity was chosen as it is the intensity type described in the Canadian 24-hour movement guidelines for children and youth aged 5 to 17 years [62]. Device measurement of physical activity was chosen as it has been shown to provide a more accurate representation of a child’s movement behaviors [63]. When multiple effect sizes based on different measurements of family functioning were presented, parental self-report of family functioning was prioritized. Parental self-reporting has been shown to provide a more accurate picture of a family’s functioning compared to children’s reports, which can be more susceptible to variance [64]. Lastly, an aggregate of values was taken for studies that presented separate effect sizes and significance levels for males and females, children and youth, or mothers and fathers.

A Fischer transformation calculator was used to standardize the effect sizes and allow for interquartile and median effect size calculations [65]. The prioritized values were placed into a Google Sheets document, and the functions “Quartile” and “Median” were used to calculate the interquartile and median effect sizes for each of their respective domains. A minimum of three studies were required per domain of family functioning to perform calculations. Strength of effect sizes were categorized as following Cohen (1992) recommendations: Small *r* =.10, medium *r* =.30, large *r*≥.50 [66].

#### Secondary analyses

Since not all studies presented effect sizes, a rubric for determining the valence and consistency of findings was implemented as an additional means of synthesis [67]. A theme was classified as follows: positive pathway if more than 59% of studies reported a positive effect; negative pathway if more than 59% of studies reported a negative effect; inconclusive if 34%–59% of studies found an association in either direction; and (4) no association if fewer than 34% of studies showed any association [67]. Statistical significance (*p* < 0.05) needed to be present to conclude if there was a positive or negative interaction. In the studies where an aggregate of multiple measurements was necessary, the paper was deemed significant if 50% or more of the tests in a given category provided significant results [37, 68]. A minimum of two studies was required to perform an analysis.

## Results

### Study selection

**Fig. 1:**
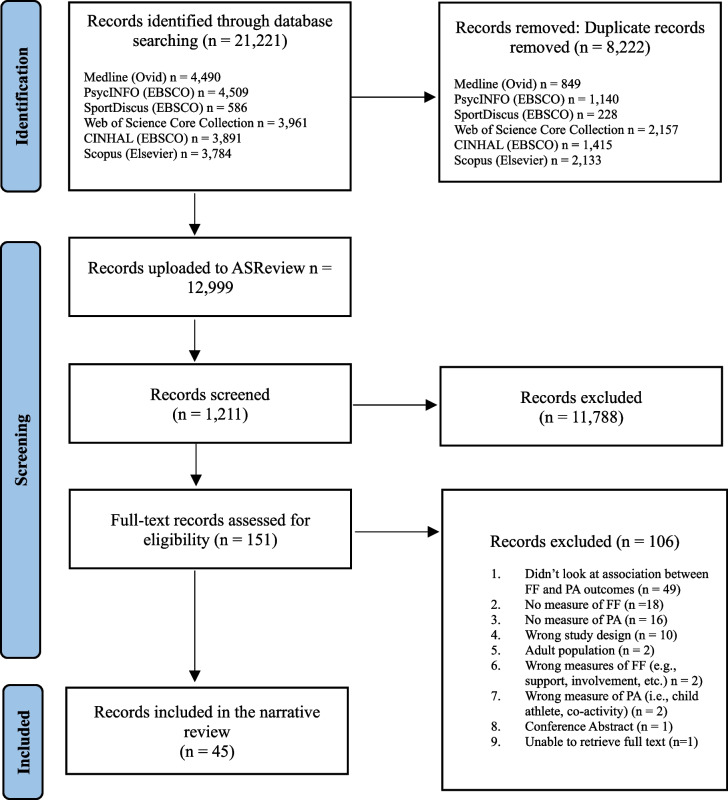
PRISMA Flow Diagram. Source inclusion process. Adapted from PRISMA Statement, Page et al. [44].

The search strategy was run on January 9th, 2024, across the six databases and yielded a total of 21,221 items. After uploading the search results to reference manager software EndNote [69], YFS used the duplicate remover function to remove a total of 8,128 articles. Further manual de-duplication of 94 articles by YFS, provided a total of 12999 articles to be screened. The potentially relevant articles were uploaded to ASReview for title and abstract screening. YFS screened 275 studies before hitting 50 back-to-back irrelevant studies, satisfying the initially chosen stop criterion. HH reviewed the 50 irrelevant articles and opted to include one of the articles for further screening. YFS continued screening, and for the second round of screening a stop criterion of 25 was agreed upon by RER and HH. Agreement of both YFS and HH on the exclusion of the 25 irrelevant articles was required to move into the full-text screening phase. YFS screened a total of 1,211 articles before satisfying the stop criterion. A total of 151 relevant articles were exported from ASReview to proceed to full-text screening using Covidence [70]. YFS and HH independently screened the 151 relevant articles. Conflicts were resolved first with YFS re-reading articles and communicating the reason for exclusion or inclusion with HH. Studies that needed further clarification were sent to RER for final decision. Of the 151 full texts screened, 43 were included for data extraction and analysis (see Fig. 1).

### Study characteristics

Table 2 presents the characteristics of the 43 included studies. More than 70 percent of the included studies measured participants between the ages of 13 to 17, with only 30% of the studies measuring children between the ages of five to 12. Most of the studies (k = 36) were cross-sectional in design, and the rest (k = 7) presented with a longitudinal design. General family functioning (k = 20), cohesion (k = 19), and communication (k = 10) were the most measured domains of family functioning. Conflict (k = 6), affective environment (k = 3), household chaos (k = 1), and organization (k = 1), were the least measured.

**Table 2:** Overall Study Characteristics

	k = 43 Samples	Percentage
Sample Size (M=4,874.14, SD = 12,238.62)		
Mean Age		
5-12	13	30.23%
13-17	30	69.77%
Population Measured		
Mother-Child Dyad	5	11.63%
Father-Child Dyad	1	2.33%
Family-Child Dyad (Parent not specified)	18	41.86%
Child/Youth Only	19	44.19%
Geographic Location		
North America	23	53.49%
Europe	11	25.58%
Asia	10	20.93%
Oceania	1	2.33%
Study design		
Longitudinal	7	16.28%
Cross-Sectional	36	83.72%
Domain of Family Functioning Measured		
Cohesion	19	44.19%
Communication	10	20.93%
Conflict	6	13.95%
Organization	1	2.33%
Affective Environment	3	6.98%
Family Problem-Solving Ability	1	2.33%
Household Chaos	1	2.33%
General Family Functioning (i.e., Family Relationship)	20	46.51%
Family Functioning Measurement		
The Inventory of Parent and Peer Attachment (IPPA-R)	2	4.65%
The Resnick Family Connectedness Scale	1	2.33%
The Family Closeness Scale	1	2.33%
The Family Adaptability and Cohesion Evaluation Scale (FACES)	3	6.98%
The Family Life Questionnaire	1	2.33%
The Parent-Adolescent Communication Scale	1	2.33%
The Iowa Family Interaction Rating Scale	1	2.33%
The Confusion Hubbub, and Order Scale (CHAOS)	1	4.33%
The Family APGAR Scale	3	6.98%
The Athlete's Family Environment Questionnaire	1	2.33%
The Scale of the Perception of Family Relations	1	2.33%
The Family Assessment Device (60-item)	2	4.65%
The Family Environment Scale	2	4.65%
The Parental Attachment Scale	1	2.33%
Generated scale based on previously tested devices or subscale embedded into a questionnaire not related to family functioning	23	53.49%
Physical Activity Measurement		
Device Measurement (e.g., accelerometry, heart rate monitor, movement sensor)	4	9.30%
Self Report	39	90.70%
The Physical Activity Questionnaire (PAQ)	3	7.69%
The Physical Activity Rating Questionnaire for Children and Youth	1	2.56%
The Godin-Shephard Leisure Time Physical Activity Questionnaire	3	7.69%
Leung’s Physical Activity Rating Scale	1	2.56%
Self-Administered Physical Activity Checklist	2	5.13%
The International Physical Activity Questionnaire	1	2.56%
WHO’S Global Physical Activity Questionnaire	1	2.56%
The Physical Day Activity Recall	1	2.56%
Other (i.e., study created self-report questionnaires generated from previously tested physical activity measurement devices)	25	64.10%

The sample was mixed in terms of measurement devices used to evaluate family functioning and physical activity. A wide variety of self-report questionnaires were used to measure family functioning and physical activity. Many of the studies (family functioning k = 23, physical activity k = 25) generated questionnaires based on previously tested devices to suit the research question of their study. Physical activity measurement also included device measurements, such as accelerometry (k = 4).

### Risk of bias assessment outcome

The risk of bias assessment (Table 3) displayed a few potential areas of bias in the included studies. Firstly, 73 percent of the included studies did not perform a prior sample size calculation. Secondly, 65 percent of the included studies did not adequately address or describe non-respondents. Lastly, 48 percent of studies did not declare any sources of funding or conflicts of interest. These methodological gaps may compromise the validity, reliability and generalizability of the included studies.

**Table 3:** Assessment of the included studies according to the Appraisal Tool for Cross-Sectional Studies (AXIS) (k = 43)

	Q1	Q2	Q3	Q4	Q5	Q6	Q7	Q8	Q9	Q10	Q11	Q12	Q13	Q14	Q15	Q16	Q17	Q18	Q19	Q20
Li et al., (2016) [96]	Y	Y	N	Y	Y	Y	Y	Y	Y	Y	Y	Y	N	Y	Y	Y	Y	Y	Y	Y
Botero-Carvajal et al., (2023) [95]	Y	Y	N	Y	Y	Y	N	Y	N	Y	Y	Y	Y	N	Y	Y	Y	Y	Y	Y
Kleszczewska et al., (2018) [78]	Y	Y	N	Y	Y	Y	N	N	Y	Y	Y	Y	Y	N	Y	Y	Y	Y	N	Y
Field et al., (2001) [97]	Y	Y	N	N	N	N	N	Y	N	Y	N	N	Y	N	N	Y	Y	N	Y	N
Oman et al., (2018) [93]	Y	Y	N	Y	Y	Y	N	N	Y	Y	Y	Y	Y	N	N	Y	Y	Y	N	N
Ryan & Kaskas (2023) [98]	Y	Y	Y	Y	Y	Y	Y	Y	Y	Y	Y	Y	N	Y	Y	Y	Y	Y	Y	Y
Leppard & Dufur (2022)[80]	Y	Y	N	N	Y	Y	Y	Y	Y	Y	Y	Y	Y	N	Y	Y	Y	Y	Y	Y
Sabo et al., (1999) [89]	Y	Y	N	Y	Y	Y	N	Y	Y	Y	N	Y	Y	N	Y	Y	Y	Y	Y	N
Tan et al., (2023) [72]	Y	Y	N	Y	Y	Y	Y	Y	Y	Y	Y	Y	N	Y	Y	Y	Y	Y	N	Y
Van Hulst et al., (2023)	Y	Y	N	Y	Y	Y	N	Y	Y	Y	Y	Y	Y	N	Y	N	Y	Y	N	Y
Berge et al., (2019) [99]	Y	Y	Y	Y	Y	Y	N	Y	Y	Y	Y	Y	N	N	Y	Y	Y	Y	N	Y
Yang et al., (2014) [90]	Y	Y	N	Y	Y	Y	N	Y	Y	Y	Y	Y	Y	Y	N	Y	Y	Y	Y	N
Gilic et al., (2020) [85]	Y	Y	N	Y	Y	Y	N	Y	Y	Y	Y	Y	Y	N	Y	Y	Y	Y	N	Y
Loprinzi (2015) [27]	Y	Y	N	Y	Y	Y	N	N	N	Y	Y	Y	N	N	Y	Y	Y	Y	Y	Y
McArthur et al., (2023) [100]	Y	Y	N	Y	Y	Y	Y	Y	N	Y	Y	Y	N	Y	Y	Y	Y	Y	N	Y
Lebron et al., (2018) [79]	Y	Y	N	Y	Y	Y	N	Y	Y	Y	Y	Y	Y	N	Y	Y	Y	Y	Y	Y
Carter et al., (2007) [101]	Y	Y	N	Y	Y	Y	N	Y	Y	Y	Y	Y	N	Y	Y	Y	Y	Y	Y	Y
Chen (2002) [71]	Y	Y	Y	Y	Y	Y	N	Y	Y	Y	Y	Y	Y	N	Y	Y	Y	Y	Y	Y
Mackay (2008) [102]	Y	Y	N	Y	Y	Y	Y	Y	Y	Y	Y	N	Y	Y	Y	Y	Y	Y	Y	Y
Shennar-Golan & Walter (2018) [82]	Y	Y	N	Y	Y	Y	N	Y	Y	Y	Y	Y	Y	N	Y	Y	Y	Y	N	Y
Kobayashi et al., (2019) [92]	Y	Y	Y	Y	Y	Y	Y	Y	Y	Y	Y	Y	Y	Y	Y	Y	Y	Y	Y	Y
Ostrowska-Karpisz et al., (2018) [81]	Y	Y	N	Y	Y	Y	N	Y	Y	Y	Y	Y	Y	N	Y	Y	Y	Y	Y	N
Bigman et al., (2015) [36]	Y	Y	N	Y	Y	Y	Y	Y	Y	Y	Y	Y	N	Y	Y	Y	Y	Y	N	Y
Ornelas et al., (2017) [131]	Y	Y	Y	Y	Y	Y	N	Y	Y	Y	Y	Y	N	Y	Y	Y	Y	Y	N	N
Dong et al., (2019) [103]	Y	Y	N	Y	Y	Y	N	N	N	Y	Y	Y	Y	N	Y	Y	Y	Y	N	Y
Zambon et al., (2006) [94]	Y	Y	N	Y	Y	Y	N	Y	N	N	Y	Y	Y	N	Y	Y	Y	Y	Y	N
Ghaffari et al., (2020) [104]	Y	Y	N	Y	Y	Y	N	Y	Y	Y	Y	Y	N	N	Y	Y	Y	Y	N	Y
Sukys et al., (2015) [84]	Y	Y	N	Y	Y	Y	N	N	Y	Y	Y	Y	Y	N	Y	Y	Y	Y	Y	Y
Knox & Muros (2017) [105]	Y	Y	Y	Y	Y	Y	Y	Y	Y	Y	Y	Y	N	Y	Y	Y	Y	Y	N	Y
Yonghe (2021) [132]	Y	Y	N	Y	Y	Y	Y	Y	Y	Y	Y	Y	N	Y	Y	Y	Y	Y	Y	N
Dzewaltowski et al., (2008)[74]	Y	Y	N	Y	Y	Y	N	Y	Y	Y	Y	Y	Y	N	Y	Y	Y	Y	Y	Y
Ho et al., (2015) [106]	Y	Y	Y	Y	Y	Y	Y	Y	Y	Y	Y	Y	N	N	Y	Y	Y	Y	N	Y
Carbert et al., (2019) [77]	Y	Y	N	Y	Y	Y	Y	Y	Y	Y	Y	Y	N	N	Y	Y	Y	Y	N	Y
Knoester & Fields (2020) [75]	Y	Y	N	Y	Y	Y	N	N	N	Y	N	Y	Y	N	Y	Y	Y	Y	N	N
Aira et al., (2023) [91]	Y	Y	Y	Y	Y	Y	N	Y	Y	Y	Y	Y	Y	N	Y	Y	Y	Y	N	Y
Chen et al., (2006) [76]	Y	Y	N	Y	Y	Y	N	Y	Y	Y	Y	Y	Y	N	Y	Y	Y	Y	Y	Y
Suris & Parera (2005) [83]	Y	Y	N	Y	Y	Y	Y	N	N	Y	Y	Y	N	Y	Y	Y	Y	Y	Y	N
Xiao et al., (2021) [133]	Y	Y	N	Y	Y	Y	N	Y	Y	Y	Y	Y	Y	N	Y	Y	Y	Y	N	N
Melguizo-Ibanez et al., ., (2021) [107]	Y	Y	N	Y	Y	Y	N	Y	Y	Y	Y	Y	Y	N	Y	Y	Y	Y	N	Y
Zurita-Ortega et al., ., (2023) [73]	Y	Y	Y	Y	Y	Y	Y	Y	Y	Y	Y	Y	N	Y	Y	Y	Y	Y	N	Y
Riley-Lawless (2000) [88]	Y	Y	Y	Y	Y	Y	N	Y	Y	Y	Y	Y	Y	Y	Y	Y	Y	Y	Y	Y
Berge et al., (2013) [108]	Y	Y	N	Y	Y	Y	N	Y	Y	Y	Y	Y	N	N	Y	Y	Y	Y	Y	Y
Coviak (1998)[86]	Y	Y	Y	Y	Y	Y	Y	Y	Y	Y	Y	Y	N	Y	Y	Y	Y	Y	Y	Y
Total Y	43	43	12	41	42	42	17	35	34	41	39	40	24	16	39	42	42	42	22	31

Q1: Were the aims/objectives of the study clear? Q2: Was the study design appropriate for the stated aim(s)? Q3: Was the sample size justified? Q4: Was the target/reference population clearly defined? (Is it clear who the research was about?) Q5: Was the sample frame taken from an appropriate population base so that it closely represented the target/reference population under investigation? Q6: Was the selection process likely to select subjects/participants that were representative of the target/reference population under investigation? Q7: Were measures undertaken to address and categorise non-responders? Q8: Were the risk factors and outcome variables measured appropriate to the aims of the study? Q9: Were the risk factor and outcome variables measured correctly using instruments/measurements that had been trialed, piloted, or published previously? Q10: Is it clear what was used to determine statistical significance and/or precision estimates? (e.g., p values CIs) Q11: Were the methods (including statistical methods) sufficiently described to enable them to be repeated? Q12: Were the basic data adequately described? Q13: Does the response rate raise concerns about non-response bias? Q14: If appropriate, was information about non-responders described? Q15: Were the results internally consistent? Q16: Were the results for the analyses described in the methods, presented? Q17: Were the authors’ discussions and conclusions justified by the results? Q18: Were the limitations of the study discussed? Q19: Were there any funding sources or conflicts of interest that may affect the authors’ interpretation of the results? Q20: Was ethical approval or consent of participants attained?

### Family functioning outcomes

Tables 4 and 5 report the effects and significance of the association between child physical activity and family functioning, respectively. A total of 37 studies reported effect size calculations and were thus included in the interquartile range and median calculations. Eleven studies presented effect sizes for children, and 31 studies presented effect sizes for youth. All studies performed significance calculations and were thus included in the thematic analysis. The full extracted results can be found in Appendix D.

**Table 4:** Synthesis of Effect Sizes of Studies Including Children Aged Five to 12 years (k=11)

Family Functioning Construct	Studies with an Effect Size	Median *r*	Interquartile Range
General Family Functioning	Loprinzi (2015) [27], Chen (2002) [71], Tan et al., (2023) [72] Ghaffari et al., (2019), Zurita-Ortega et al., (2023) [73]	0.09	-0.06 - 0.09
Cohesion	Loprinzi (2015) [27], McArthur et al., (2021), Dzewaltowski et al., (2008) [74], Wing Ho et al., (2015), [75], Yonghe (2021) [132]	0.06	0.05 - 0.22
Communication	Loprinzi (2015) [27], Chen (2002) [71]	N/A	N/A
Conflict	Loprinzi (2015) [27]	N/A	N/A
Organization	Chen (2002) [71]	N/A	N/A
Affective Environment	Chen (2002) [71], Chen (2006) [76]	N/A	N/A
Family Problem-Solving Ability	Chen (2002) [71]	N/A	N/A
Household Chaos	N/A	N/A	N/A

**Table 5:** Synthesis of Effect Sizes of Studies Including Youth Aged 13 to 17 years (k=26)

Family Functioning Construct	Studies with an Effect Size	Median *r*	Interquartile Range
General Family Functioning	Carbert et al., (2019) [77], Kleszcezewska et al., (2018) [78], Ryan & Kaskas (2023) [130], Lebron et al., (2018) [79] Leppard & Dufur (2022) [80], Ostrowska-Karpi et al., (2018) [81], Shennar-Golan & Walter (2018) [82], Dong et al., (2018), Berge et al., (2012), Suris & Parera (2005) [83]	0.04	0.02 - 0.06
Cohesion	Li et al., (2015), Sukys et al., (2015) [84], Bigam et al., (2015) [36], Gilic et al., (2020) [85], Mackay (2007), Coviak (1998) [86], Ornelas et al., (2007) [87], Riley-Lawless (2000) [88], Sabo et al., (1999) [89],Yang et al., (2014) [90]	0.09	0.07 - 0.14
Communication	Sukys et al., (2015) [84], Lebron et al., (2018) [79], Ornelas et al., (2007) [87],, Aira et al., (2023) [91], Kobayashi et al., (2019) [92], Oman et al., (2018) [93], Zambon et al., (2006) [94],	0.17	0.09 - 0.40
Conflict	Bigam et al., (2015) [36], Gilic et al., (2020) [85], Riley-Lawless (2000) [88], Botero-Carvajal et al., (2023) [95], Xiao et al., (2021) [133],	-0.09	-0.21 - 0.02
Organization	N/A	N/A	N/A
Affective Environment	N/A	N/A	N/A
Family Problem-Solving Ability	N/A	N/A	N/A
Household Chaos	N/A	N/A	N/A

#### General family functioning

Twenty studies reported outcomes of general family functioning (i.e., parent-child relationship) [27, 71–73, 77–83, 97–99, 103–105, 107, 108]. 15 of the 20 studies reported effect sizes and were included in the median and interquartile range calculations. Five studies assessed children [27, 71–73, 104]. The analysis yielded median and interquartile range (IQR) calculations of r = 0.09 (IQR: −0.06to 0.09). Ten studies presented results for youth [77–83, 98, 103, 108]. The analysis yielded median and interquartile range calculations of *r* = 0.04 (IQR: 0.02 to 0.06). These small median effect sizes suggest a weak positive association between general family functioning and youth physical activity (Tables 6, and 7 ).

**Table 6:** Significance of Included Studies Including Children Aged 5 to 12 years (k=13)

Family Functioning Construct	Count of Papers with a Significant Positive Effect	Count of Papers with No Significant Positive Effect	Count of Papers with a Significant Negative Effect	Count of Papers with No Significant Negative Effect	Overall Relationship
General Family Functioning	Ghaffari et al., (2019), Berge et al., (2023) [99], Zurita-Ortega et al., (2019) [73],	Tan et al., (2023) [72], Melguizo-Ibanez et al., (2020)	Loprinzi (2015) [27]	Chen (2002) [71]	?
Cohesion	Yonghe (2021) [132], Berge et al., (2019), [99], Dzewaltowski et al., (2008) [74], Knoester & Fields (2020) [75]	McArthur et al., (2021), Wing Ho et al., (2015)	Loprinzi ([27])		?
Communication		Chen ([71]), Berge et al., ([99])	Loprinzi ([27])		?
Conflict			Loprinzi ([27])		N/A
Organization				Chen (2002) [71]	N/A
Affective Environment	Chen et al., (2006) [76]	Berge et al., (2019) [99]		Chen (2002) [71]	ns
Family Problem-Solving Ability				Chen (2002) [71]	N/A
Household Chaos					N/A

At least two studies were required for a theme and an estimate of effect. + = positive association (>59% of studies), - = negative association (>59% of studies),? = indeterminate (34-59% of studies showing an association) and ns = no association (<34% of studies showing any association)

**Table 7:** Significance of Included Studies Including Youth Aged 13 to 17 years (k=30)

Family Functioning Construct	Count of Papers with a Significant Positive Effect	Count of Papers with No Significant Positive Effect	Count of Papers with a Significant Negative Effect	Count of Papers with No Significant Negative Effect	Overall Relationship
General Family Functioning	Kleszcezewska et al., (2018) [78], Knox & Muros (2017) [105], Dong et al., (2018), Field et al., (2011), Leppard & Dufur (2022) [80], Berge et al., (2012), Shennar-Golan & Walter (2018) [82], Suris & Parera (2005) [83], Ostrowska-Karpi et al., (2018)	Ryan & Kaskas (2023) [130]	Lebron et al., (2018) [79]	Carbert et al., ([77])	+
Cohesion	Sabo et al., (1999) [89], Mackay (2007), Bigam et al., (2015) [36], Field et al., (2011), Li et al., (2015), Ornelas et al., (2007) [87], Sukys et al., (2015) [84], Coviak (1998) [86], Carter et al., (2007) [101]	Yang et al., ([90]), Riley-Lawless ([88])	Gilic et al., (2020) [85]		+
Communication	Sukys et al., (2015) [84], Ornelas et al., (2007) [87]), Aira, et al., (2023) [91]), Kobayashi et al., (2019) [92], Oman et al., (2018) [93], Zambon et al., (2016) [94]	Lebron et al., ([79])			+
Conflict		Bigam et al., (2015) [36], Riley-Lawless (2000) [88]	Gilic et al., (2020)[85], Botero-Carvajal et al., (2023) [95], Xiao et al., (2021) [133]		-
Organization					N/A
Affective Environment					N/A
Family Problem-Solving Ability					N/A
Household Chaos		Van Hulst et al., (2023)			N/A

At least two studies were required for a theme and an estimate of effect. + = positive association (>59% of studies), - = negative association (>59% of studies),? = indeterminate (34-59% of studies show an association) and ns = no association (<34% of studies showing any association)

The spread of the studies across the four categories resulted in an indeterminate relationship between general family functioning and child physical activity. Nine studies reported a significant positive effect between general family functioning and youth physical activity resulting in a positive relationship [78, 80–83, 97, 103, 105, 108]. Positive links between youth physical activity and general family functioning suggest a promising relationship, despite unclear findings for child physical activity.

#### Cohesion

Nineteen studies reported outcomes of cohesion, with 16 studies presenting effect sizes [27, 36, 74, 75, 84–90, 96, 97, 99–102, 106, 109]. Six studies measured children yielding a median and interquartile range of r =0.06 (IQR: 0.05 to 0.22) [27, 74, 75, 100, 106, 109]. Ten studies measured youth resulting in median and interquartile range calculations of r =0.09 (IQR: 0.07 to 0.14) [36, 84–90, 96, 102]. All 19 studies reported significance values and were included in the thematic analysis. The analysis between child physical activity and cohesion yielded an indeterminate effect as the studies were spread across the four categories. The youth subcategory presented nine studies that had a significant positive effect [36, 84, 86, 87, 89, 96, 97, 101, 102], two with an insignificant positive effect [88, 90], and one with a significant negative effect [85]. Thus, the family functioning domain of cohesion had an overall positive association with youth physical activity.

#### Communication

Eleven studies reported outcomes of a relationship between communication and child and youth physical activity [27, 71, 79, 84, 87, 91–94, 99]. Two studies [27, 71] presented effect sizes in the child age category and seven [79, 84, 87, 91–94] in the youth age category (r = 0.17; IQR: 0.09 to 0.40). In the child age category two studies [71, 99] had no significant positive effect, and one study had a significant negative effect [27], resulting in an indeterminate theme. The 13 to 17 age category reported a positive association between communication and physical activity as six studies [84, 87, 91–94] presented significant positive effects, and one study reported a nonsignificant effect [79]. Overall, these findings suggest that better family communication may be associated with greater physical activity in youth.

#### Conflict

Six studies reported measures of conflict [27, 36, 85, 88, 95, 110]. One study [27] presented effect sizes for children, and five studies [36, 85, 88, 95, 110] reported effect sizes for youth, r = −0.11 (IQR: −0.19 to −0.02). All studies reported significance values and were included in the thematic analysis.

Three studies [85, 95, 110] reported a significant negative effect and two studies presented insignificant positive effects in the youth age category [36, 88]. This resulted in the domain of conflict having a significant negative relationship with youth physical activity. This trend toward a negative relationship highlights an overall association between higher family conflict and lower youth physical activity.

#### Organization, family problem-solving ability, affective environment, and household chaos

The domains of organization, family problem-solving ability, affective environment, and household chaos had minimal literature making it not possible to perform an analysis. The domain of affective environment in the child age category had three studies [71, 76, 99] that presented significance values however, these values were all evenly dispersed across categories, which resulted in no association.

## Discussion

To our knowledge, this review is the first to summarize and appraise the interrelationship between the general domains of family functioning (general family functioning, cohesion, communication, conflict, organization, household chaos, affective environment, and family problem-solving ability) and child (five to twelve) and youth (13 to 17) physical activity. Across the six databases searched, our review identified 43 studies that met the inclusion criteria. The domains of general family functioning, cohesion, communication, and conflict were the most sampled domains of family functioning. Despite some limitations, the present sample represents a rich dataset to appraise the relationship between family functioning and child and youth physical activity.

General family functioning is the dynamic interplay of the seven domains (cohesion, communication, conflict, organization, household chaos, affective environment, and family problem-solving ability) of a family’s functioning [29]. While the analysis presented small effect sizes [66], these results are likely due to the multifaceted nature of family functioning [26]. As examining each of the seven family functioning domains is vital to provide a complete and balanced overview of a family’s general functioning [11], it could be understandable that the effect size for an unstandardized aggregate definition of family functioning across studies presents with a small effect size. Given how varied each of the family functioning domains are, some domains may show no association with child and youth physical activity, while others may show a positive or negative association. When these domains are aggregated under the umbrella of general family functioning, the resulting effect size may be diluted or masked due to the variability across the contributing subdomains. Furthermore, many studies operationalized the definition of family functioning differently. Some considered general family functioning to encompass just cohesion and communication [79, 87], while others chose to include more subdomains [27, 71, 99]. This variability again could lend itself to the small effect size, as analysis of only a few of the subdomains of family functioning could lead to a varied and unbalanced relationship between general family functioning and child and youth physical activity. So, despite small number of studies in this domain, and the varied nature of the literature this analysis begins to suggest the impactful relationship between family functioning and child and youth physical activity. Furthermore, it is important to highlight that the relationship between general family functioning and child and youth physical activity is likely best understood through the analysis of its subdomains. Untangling the complex inner workings of the family unit allows us to gain a clearer understanding of how each domain may associate with child and youth physical activity [111]. In turn, this approach allows future interventions, programs, and research to be more focused and effective.

The domain of cohesion, defined as the bond between the parent and the child, presented a small effect size across both age groups, and a significant positive association with youth physical activity. Research has shown that parents who are strongly bonded to their children are more actively engaged, which in turn is associated with increased engagement in physical activity [36, 112]. For example, active engagement can manifest as parents coaching their child through learning new sporting techniques, participating in family hikes, or taking their child to the playground. Given the small effect size and positive association between cohesion and child and youth physical activity, interventions, research, and programs can take a more focused approach to increase child and youth movement by directly fostering family cohesion.

The domain of communication presented a small effect size and a significant positive association with youth physical activity. Research indicates that families with good communication are better equipped to discuss and model healthy movement behaviors [113]. Fostering open lines of communication within the family is important as it ensures that the child is receptive to suggestions surrounding healthy movement behaviors [114, 115]. Given the small effect size and positive association between communication and child and youth physical activity, future research and interventions should explore how enhancing family communication and family physical activity may mutually support one another.

The domain of conflict presented a small effect size with a significant negative association with youth physical activity. A family with a high level of conflict has been shown to create an environment where members are more likely to feel depressed, anxious, and stressed [116]. Given that there is a relationship between child and youth physical activity and family conflict, it stands to reason that the multitude of research showing the beneficial effects of physical activity on emotional well-being, depression, anxiety, and stress reactivity also applies to the relationship between conflict and child physical activity [117–119]. Therefore, families reporting lower conflict may have environments that are less stressful, anxiety and depression inducing, and more conducive to engagement in physical activity, and vice versa [119]. However, future research is needed to better understand and tease apart these potential reciprocal influences.

Across both age ranges the domains of organization, affective environment, family problem-solving ability, and household chaos did not have enough literature to perform further analysis. It is important to note that the domains of family functioning need to be equally researched to ensure a complete understanding of the complex and dynamic interplay that the domains all lend to the overall picture of a family’s functioning and, in turn, child and youth physical activity. For example, families that are highly organized with good problem-solving abilities and low chaos can create a routine, delegate pick-up and drop-off tasks, and maneuver potential scheduling conflicts. Research has shown that families with clear roles and responsibilities are more likely to enforce routines, and maintain a clean home environment, thus creating for their child an environment more conducive to physical activity engagement [71, 120]. Further research in these areas has the potential to reveal pertinent information within the family unit that can be leveraged to support active, healthy lifestyles for children and youth. This comprehensive approach will be key to fostering environments where physical activity is not just encouraged but seamlessly integrated into daily family life.

While the effect sizes observed within this review are small, they align with prior research, indicating that these findings are still meaningful. Firstly, prior reviews exploring more specific markers of family function, such as parental support [37] or involvement [38] presented slightly larger effect sizes. These indirect measurements of family functioning are more tangible precise markers of a family’s functioning. In contrast, the general domains of family functioning analyzed in this review are more abstract and thus harder to conceptualize, as they present many complex inputs and outputs [26, 111]. Therefore, given this complexity, it is coherent that this review presented a smaller effect size. Furthermore, given that physical activity is just one possible outlet or antecedent of a family’s functioning it adds to the reasoning behind the small effect sizes.

While the literature supporting the association between youth physical activity and family functioning is more robust, allowing for further evaluation into the subdomains of communication and conflict, the domains that did present with enough literature to support analysis in both children and youth (general family functioning and cohesion) presented similar findings. This consistency, even with the small effect sizes, suggests that physical activity is an important contributor to a family’s functioning across multiple subdomains and age ranges. Additionally, the assessed subdomains appear to replicate the general family functioning small effect size results, with the effect appearing equally distributed. While the under-researched domains of family functioning may yield different results, this finding indicates that no single area seems to disproportionately affect child and youth physical activity. This further lends to the idea that each of the subdomains of family functioning is interconnected, and each contributes an important piece to forming a family’s overall functioning. As it stands, physical activity is associated with several of the domains of family functioning and thus could be a meaningful component of family functioning, despite the small effect sizes.

While the included studies presented a rich dataset to appraise, there were a few notable limitations. Firstly, 70% of the included studies sampled youth. While understanding the relationship between youth physical activity and family functioning is crucial as adolescence marks a time of significant developmental changes [121], it is important to note that this period does not present a complete picture. During the ages of five to twelve, children spend more time in the presence of their family and have movement behaviors/habits that have not yet been developed and are thus easily malleable [122]. Therefore, it is recommended that future literature focus on a younger age group.

Secondly, many of the included studies did not perform a prior sample size calculation, adequately address or describe non-respondents, or declare any sources of funding or conflicts of interest. These quality limitations have the potential to introduce bias that could influence the overall confidence of the review’s findings.

Thirdly, as most of the studies in our review employed a cross-sectional study design, this likely provided but a snapshot of the relationship between a family’s functioning and child and youth physical activity. A parent-child relationship is dynamic, constantly evolving as the child ages and develops [123]. Given that a child’s physical activity behaviors naturally change as they age, due to factors such as environmental and/or physical changes, it is important to study the relationship between family functioning and child and youth physical activity across a child’s development [124]. Therefore, it is recommended that future research employ a longitudinal study design to allow for further insight into how development from childhood to adolescence impacts the relationship between family functioning and child and youth physical activity.

Additionally, all studies used a self-report questionnaire to measure family functioning and child and youth physical activity. Given the nature of self-report questionnaires to be somewhat one-dimensional, it can make it challenging to fully understand the intricate relationship between a parent and a child. While there are standardized and previously evaluated self-report questionnaires, many of the studies chose to design questionnaires based on the needs of their study. Quantifying a complicated relationship, like that of a parent and a child, with a Likert scale can only provide a surface-level understanding of the relationship. In the future, it is recommended that multiple data collection methods be used. For example, self-report questionnaires coupled with interviews could provide researchers with a deeper understanding of the parent-child relationship and further expand our current understanding of the relationship between family functioning and child physical activity. Furthermore, as the domains of family functioning do show promise to impact child and youth movement behaviors positively, it would be pertinent for future authors to use standardized measures that cover all the mentioned domains of family functioning. As there is yet to be a comprehensive family functioning assessment device, it would be best to use an aggregate of available devices to ensure that all domains of family functioning are covered. Use of the McMaster Family Assessment Device, the Family Environment scale, and the Confusion, Hubbub and Order Scale (CHAOS) is recommended in future studies [125–127]. This would not only fill the gap in the understudied domains of family functioning but also standardize the field to allow for easier comparison and interpretation. The same recommendations can be applied to the measurement of physical activity. To achieve standardization and accurate results within the field, device measurement, such as use of accelerometers would be considered best practice [128].

Further, despite the authors following PRISMA reporting protocol as closely as possible, the current review does present some limitations. Firstly, the use of an AI screening tool during the title and abstract screening portion of this study could have allowed for the exclusion of potentially relevant articles. However, a study evaluating the performance of active learning models during screening of systematic reviews demonstrated that the active learning model used in the current study finds 95% of relevant publications [129]. Additionally, human screening is not without its faults and can often be prone to error [51]. Secondly, the heterogeneous measurement of family functioning and physical activity across studies made it challenging to synthesize and interpret the results. We believe this is an appropriate first assessment of the association between family functioning and child and youth physical activity, but a more refined meta-analysis in the future may yield different findings.

## Conclusions

Our review appraised 43 studies to determine the association between the general domains of family functioning and child and youth physical activity. Our analysis revealed that the domains of general family functioning and cohesion had small effect sizes and an indeterminate association with child physical activity. The domains of general family functioning, cohesion, communication, and conflict all had small effect sizes and a positive (general family functioning, cohesion, and communication) or negative (conflict) association with youth physical activity. The domains of affective environment, organization, household chaos, and family problem-solving ability require further research for appraisal. We recommend that future research aim to explore the understudied domains of family functioning to provide a complete picture of the impacts of family functioning on child physical activity. Furthermore, given the heterogeneous nature of the literature, it is recommended that future research use previously tested and standardized measures to evaluate the domains of child/youth physical activity and family functioning.

## Supplementary Information

Supplementary Material 1.

Supplementary Material 2.

Supplementary Material 3.

Supplementary Material 4.

## Data Availability

All data used in this article can be found in Appendix C: Full Data Extraction.

## Abbreviations

PRISMA: Preferred Reporting Items for Systematic Reviews and Meta-Analyses
PROSPERO: International prospective register of systematic reviews
AI: Artificial Intelligence
TF-IDF: Term frequency-inverse document frequency
AXIS: Appraisal tool for cross-sectional studies
IQR: Interquartile range
CHAOS: Confusion, hubbub and order scale
