# Supplementary material for: Association between child and youth physical activity and family functioning: a systematic review of observational studies
Source: Int J Behav Nutr Phys Act. 2025 Jul 22;22:101. doi: 10.1186/s12966-025-01782-z (PMC12281683; doi:10.1186/s12966-025-01782-z)
Supplement: Supplementary file 1 — Supplementary Material 1. [file 12966_2025_1782_MOESM1_ESM.docx]

**Appendix A**

**Search Strategy for Each of the Included Databases**

**Medline (Ovid)**

**Family Functioning**

family relations/ or intergenerational relations/ or parent-child relations/ or father-child relations/ or mother-child relations/ or Family Characteristics/

(((house* or family or families or "Mother-child" or "Father-child" or "Parent-child" or intergenerational) adj3 (relation* or routine* or function or Functioning or cohesion or "social environment" or interaction or interactions or organization or "affective environment" or "problem solving" or communication or communications or involvement or closeness or dynamic or dynamics or chaos or disorder or "verbal behavior*" or adaptability or "cooperative behavior" or "interpersonal relations" or warmth or "affective expression" or "affective involvement" or connectedness or dysfunction)) or "family function" or "family functionality")

**Child/Youth**

adolescent/ or child/ or child, preschool/ or child, adopted/ or child, foster/

("Child*" or "Preschool*" or "Adolescen*" or "Pediatric*" or "Paediatric" or "Youth*" or "Elementary student*" or "High school student*" or "elementary school student*" or teenage* or girl or boy or boys or girls or "schoolgirl*" or "schoolboy*" or "schoolchild*" or "schoolkid*" or kid or kids)

**Physical Activity**

exercise/ or gymnastics/ or running/ or swimming/ or walking/ or dancing/ or sports/ or baseball/ or basketball/ or bicycling/ or boxing/ or football/ or golf/ or hockey/ or martial arts/ or racquet sports/ or tennis/ or skating/ or snow sports/ or skiing/ or soccer/ or team sports/ or "track and field"/ or volleyball/ or water sports/ or weight lifting/ or wrestling/ or youth sports/ or motor activity/ or physical conditioning, human/ or physical exertion/ or physical fitness/ or endurance training/ or weight lifting/ or wrestling/ or Motor Activity/

(((motor or physical or aerobic or anaerobic) adj3 (behavior or activity or activities or skill or control or performance or function or fitness or exercise or training or conditioning or condition or health or education or literacy or recreation or program)) or ("physical activity" or bicycling or swimming or tobogganing or "winter play" or "park play" or walking or skating or basketball or "home exercise" or "weight training" or trampoline or "skipping rope" or "jump rope" or "indoor chores" or "outdoor chores" or tag or baseball or softball or football or soccer or volleyball or dance or handball or "mini handball" or ball-playing or "street hockey" or "floor hockey" or "ice hockey" or ringette or "ice skating" or "in-line skating" or skateboarding or "track and field" or jogging or running or golf or gymnastics or yoga or "exercise class" or badminton or tennis or kayaking or canoeing or "downhill skiing" or snowboarding or skiing or boxing or wrestling or karate or judo or tai-chi or "tae kwon do" or "cross country skiing"))

**CINAHL (EBSCO)**

**Family Functioning**

(MH "Family Functioning") OR (MH "Family Relations") OR (MH "Intergenerational Relations") OR (MH "Family Conflict") OR (MH "Parent-Child Relations") OR (MH "Father-Child Relations") OR (MH "Mother-Child Relations") OR (MH "Parent-Infant Relations") OR (MH "Father-Infant Relations") OR (MH "Mother-Infant Relations") OR (MH "Parent-Infant Bonding") OR (MH "Family Structure") OR (MH "Home Environment")

SU: house*

TI, AU, AB: ((family OR families OR "Mother-child*" OR "Father-child*" OR "Parent-child*" OR intergeneration* OR “mother-adolescent*” OR “father-adolescent*” OR “parent-adolescent*”) N3 (relation* OR routine* OR function OR functioning OR cohesion OR "social environment" OR interaction OR interactions OR organization OR "affective environment" OR "problem solving" OR communication OR communications OR involvement OR closeness OR dynamic OR dynamics OR chaos OR disorder OR "verbal behavior*" OR adaptability OR "cooperative behavior" OR "interpersonal relations*" OR warmth OR "affective expression" OR "affective involvement" OR connectedness))

**Child/Youth**

(MH "Adolescence") OR (MH "Child") OR (MH "Child, Adopted") OR (MH "Child, Abandoned") OR (MH "Children with Disabilities") OR (MH "Child, Foster") OR (MH "Child, Gifted") OR (MH "Child, Hospitalized") OR (MH "Child, Institutionalized") OR (MH "Child, Medically Fragile") OR (MH "Child, Preschool") OR (MH "Students, Elementary") OR (MH "Students, Middle School") OR (MH "Students, High School")

SU: (Child OR Adolescent)

AG: ("infancy" OR "preschool age" OR "school age" OR "childhood" OR "adolescence")

TI, AU, AB: (("Child*" OR "Preschool*" OR "Adolescen*" OR "Pediatric*" OR "Paediatric" OR "Youth*" OR "Elementary student*" OR "High school student*" OR "elementary school student*" OR teenage* OR girl OR boy OR girls OR boys OR "schoolgirl*" OR "schoolboy*" OR "schoolchild*" OR "schoolkid*" OR kid OR kids OR “young athlete*” OR “grade level” OR “kindergarten student*” OR “middle school student*” OR “intermediate school student*” ))

**Physical Activity**

(MH "Physical Activity") OR (MH "Exercise+") OR (MH "Gymnastics") OR (MH "Running+") OR (MH "Running, Distance") OR (MH "Swimming") OR (MH "Walking+") OR (MH "Dancing+") OR (MH "Sports+") OR (MH "Baseball") OR (MH "Softball") OR (MH "Basketball") OR (MH "Cycling") OR (MH "Boxing") OR (MH "Football") OR (MH "Soccer") OR (MH "Golf") OR (MH "Hockey") OR (MH "Martial Arts") OR (MH "Racquet Sports+") OR (MH "Tennis") OR (MH "Skating+") OR (MH "Ice Skating") OR (MH "Winter Sports+") OR (MH "Skiing+") OR (MH "Snow Skiing+") OR (MH "Team Sports+") OR (MH "Track and Field") OR (MH "Volleyball") OR (MH "Aquatic Sports+") OR (MH "Weight Lifting") OR (MH "Wrestling") OR (MH "Motor Activity+") OR (MH "Exertion+") OR (MH "Physical Fitness+") OR (MH "Endurance Training")

SU: (Sport OR Sports)

TI, AU, AB: (((athletic OR motor OR physical OR aerobic OR anaerobic) N3 (behavior OR activit* OR skill OR skills OR control OR performance OR function OR functioning OR fitness OR exercise OR training OR conditioning OR health OR education OR literacy OR recreation OR program*)) OR bicycling OR cycling OR swimming OR tobogganing OR "winter play" OR "park play" OR walking OR skating OR basketball OR "home exercise" OR "weight training" OR trampoline OR "skipping rope" OR "jump rope" OR "indoor chores" OR "outdoor chores" OR tag OR baseball OR softball OR football OR soccer OR volleyball OR dance OR handball OR "mini handball" OR ball-playing OR "street hockey" OR "floor hockey" OR "ice hockey" OR ringette OR "ice skating" OR "in-line skating" OR skateboarding OR "track and field" OR jogging OR running OR golf OR gymnastics OR yoga OR "exercise class" OR badminton OR tennis OR kayaking OR canoeing OR "downhill skiing" OR snowboarding OR skiing OR boxing OR wrestling OR karate OR judo OR “tai-chi” OR "tae kwon do" OR "cross country skiing" OR “cycling” OR sport OR sports)))

**APA PsycINFO (EBSCO)**

**Family Functioning**

(DE "Father Child Relations" OR DE "Mother Child Relations" OR DE "Parent Child Communication" OR DE "Father Child Communication" OR DE "Mother Child Communication" OR DE "Intergenerational Relations" OR DE "Parent Child Relations" OR DE "Family Relations" OR DE "Family Conflict" OR DE "Family Separation" OR DE "Intergenerational Relations" OR DE "Parent Child Relations" OR DE "Parental Role" OR DE "Transgenerational Patterns")

SU: house*

TI, AU, AB, KW: ((family OR families OR "Mother-child*" OR "Father-child*" OR "Parent-child*" OR intergeneration* OR “mother-adolescent*” OR “father-adolescent*” OR “parent-adolescent*”) N3 (relation* OR routine* OR function OR functioning OR cohesion OR "social environment" OR interaction OR interactions OR organization OR "affective environment" OR "problem solving" OR communication OR communications OR involvement OR closeness OR dynamic OR dynamics OR chaos OR disorder OR "verbal behavior*" OR adaptability OR "cooperative behavior" OR "interpersonal relations*" OR warmth OR "affective expression" OR "affective involvement" OR connectedness))

**Child/Youth**

(DE "Early Adolescence" OR DE "Late Adolescence" OR DE "Pediatrics" OR DE "High School Students" OR DE "Junior High School Students" OR DE "Kindergarten Students" OR DE "Preschool Students" OR DE "Primary School Students" OR DE "Grade Level" OR DE "Middle School Students" OR DE "Intermediate School Students" OR DE "Elementary School Students" OR DE "Adopted Children")

SU: (Child OR Adolescent)

AG: ("infancy" OR "preschool age" OR "school age" OR "childhood" OR "adolescence")

TI, AU, AB, KW: (("Child*" OR "Preschool*" OR "Adolescen*" OR "Pediatric*" OR "Paediatric" OR "Youth*" OR "Elementary student*" OR "High school student*" OR "elementary school student*" OR teenage* OR girl OR boy OR girls OR boys OR "schoolgirl*" OR "schoolboy*" OR "schoolchild*" OR "schoolkid*" OR kid OR kids OR “young athlete*” OR “grade level” OR “kindergarten student*” OR “middle school student*” OR “intermediate school student*” ))

**Physical Activity**

(DE "Athletic Training” OR DE "Adaptive Sports" OR DE "Athletic Participation" OR DE "Endurance" OR DE "Athletic Performance" OR DE "Physical Activity" OR DE "Aerobic Exercise" OR DE "Swimming" OR DE "Running" OR DE "Walking" OR DE "High School Sports" OR DE "Baseball" OR DE "Basketball" OR DE "Dance" OR DE "Cycling" OR DE "Football" OR DE "Martial Arts" OR DE "Judo" OR DE "Self-Defense" OR DE "Tennis" OR DE "Soccer" OR DE "Weightlifting" OR DE "Exercise" OR DE "Sports" OR DE "Yoga" OR DE "Physical Endurance" OR DE "Physical Fitness" OR DE "Physical Strength")

SU: (Sport OR Sports)

TI, AU, AB, KW: (((athletic OR motor OR physical OR aerobic OR anaerobic) N3 (behavior OR activit* OR skill OR skills OR control OR performance OR function OR functioning OR fitness OR exercise OR training OR conditioning OR health OR education OR literacy OR recreation OR program*)) OR bicycling OR cycling OR swimming OR tobogganing OR "winter play" OR "park play" OR walking OR skating OR basketball OR "home exercise" OR "weight training" OR trampoline OR "skipping rope" OR "jump rope" OR "indoor chores" OR "outdoor chores" OR tag OR baseball OR softball OR football OR soccer OR volleyball OR dance OR handball OR "mini handball" OR ball-playing OR "street hockey" OR "floor hockey" OR "ice hockey" OR ringette OR "ice skating" OR "in-line skating" OR skateboarding OR "track and field" OR jogging OR running OR golf OR gymnastics OR yoga OR "exercise class" OR badminton OR tennis OR kayaking OR canoeing OR "downhill skiing" OR snowboarding OR skiing OR boxing OR wrestling OR karate OR judo OR “tai-chi” OR "tae kwon do" OR "cross country skiing" OR “cycling” OR sport OR sports)))

**SPORTDiscus (EBSCO)**

**Family Functioning**

N/A – Subject Terms

SU: house*

TI, AU, AB, KW: ((family OR families OR "Mother-child*" OR "Father-child*" OR "Parent-child*" OR intergeneration* OR “mother-adolescent*” OR “father-adolescent*” OR “parent-adolescent*”) N3 (relation* OR routine* OR function OR functioning OR cohesion OR "social environment" OR interaction OR interactions OR organization OR "affective environment" OR "problem solving" OR communication OR communications OR involvement OR closeness OR dynamic OR dynamics OR chaos OR disorder OR "verbal behavior*" OR adaptability OR "cooperative behavior" OR "interpersonal relations*" OR warmth OR "affective expression" OR "affective involvement" OR connectedness))

**Child/Youth**

DE "TEENAGERS" OR DE "GIRLS" OR DE "BOYS" OR DE "CHILD athletes" OR DE "CHILD development" OR DE "MOTOR ability in children" OR DE "STUDENTS" OR DE "SCHOOL children" OR DE "CHILDREN" OR DE "CHILD” OR DE "DEAFBLIND children" OR DE "OUTDOOR recreation for children" OR DE "OVERWEIGHT children" OR DE "SCHOOL children" OR DE "YOUTH" OR DE "SCOUTS (Youth organization members)"

SU: (Child OR Adolescent)

TI, AU, AB, KW: (("Child*" OR "Preschool*" OR "Adolescen*" OR "Pediatric*" OR "Paediatric" OR "Youth*" OR "Elementary student*" OR "High school student*" OR "elementary school student*" OR teenage* OR girl OR boy OR girls OR boys OR "schoolgirl*" OR "schoolboy*" OR "schoolchild*" OR "schoolkid*" OR kid OR kids OR “young athlete*” OR “grade level” OR “kindergarten student*” OR “middle school student*” OR “intermediate school student*” ))

**Physical Activity**

DE "EXERCISE for children" OR DE "EXERCISE for girls" OR DE "EXERCISE for youth" OR DE "STRENGTH training" OR DE "YOGA" OR DE "PHYSICAL activity" OR DE "PHYSICAL education (Elementary)" OR DE "PHYSICAL education (Middle school)" OR DE "PHYSICAL education (Primary)" OR DE "PHYSICAL education (Secondary)" OR DE "PHYSICAL education for children" OR DE "PHYSICAL education for girls" OR DE "GYMNASTICS" OR DE "PHYSICAL fitness" OR DE "RUNNING" OR DE "JOGGING" OR DE "AEROBIC exercises" OR DE "WALKING" OR DE "DANCE" OR DE "SWIMMING" OR DE "AQUATIC sports" OR DE "ATHLETICS" OR DE "BASEBALL" OR DE "BALL games" OR DE "SPORTS" OR DE "CYCLING" OR DE "FOOTBALL" OR DE "GOLF" OR DE "BASKETBALL" OR DE "SOCCER" OR DE "HOCKEY" OR DE "RECREATION" OR DE "VOLLEYBALL" OR DE "TEAM sports" OR DE "BOXING" OR DE "TRACK & field" OR DE "WEIGHT lifting" OR DE "MARTIAL arts" OR DE "SPORTS" OR DE "WRESTLING" OR DE "AEROBIC exercises" OR DE "SKIING" OR DE "TOBOGGANING" OR DE "SNOWBOARDING" OR DE "JUMPING rope" OR DE "PHYSICAL fitness" OR DE "ANAEROBIC exercises" OR DE "HANDBALL" OR DE "STRENGTH training" OR DE "ANAEROBIC training" OR DE "ICE skating" OR DE "WINTER sports" OR DE "IN-line skating" OR DE "SKATEBOARDING" OR DE "TENNIS" OR DE "BADMINTON training" OR DE "PHYSICAL training & conditioning" OR DE "CANOES & canoeing" OR DE "KAYAKING" OR DE "KARATE" OR DE "JUDO" OR DE "TAI chi"

SU: (Sport OR Sports)

TI, AU, AB, KW: (((athletic OR motor OR physical OR aerobic OR anaerobic) N3 (behavior OR activit* OR skill OR skills OR control OR performance OR function OR functioning OR fitness OR exercise OR training OR conditioning OR health OR education OR literacy OR recreation OR program*)) OR bicycling OR cycling OR swimming OR tobogganing OR "winter play" OR "park play" OR walking OR skating OR basketball OR "home exercise" OR "weight training" OR trampoline OR "skipping rope" OR "jump rope" OR "indoor chores" OR "outdoor chores" OR tag OR baseball OR softball OR football OR soccer OR volleyball OR dance OR handball OR ball-playing OR "street hockey" OR "floor hockey" OR "ice hockey" OR ringette OR "ice skating" OR "in-line skating" OR skateboarding OR "track and field" OR jogging OR running OR golf OR gymnastics OR yoga OR "exercise class" OR badminton OR tennis OR kayaking OR canoeing OR "downhill skiing" OR snowboarding OR skiing OR boxing OR wrestling OR karate OR judo OR “tai-chi” OR "tae kwon do" OR "cross country skiing" OR “cycling” OR sport OR sports)))

**Web of Science Core Collection (Web of Science)**

**Family Functioning**

Title, Abstract, Author Keywords, Keywords Plus: ((family OR families OR "Mother-child*" OR "Father-child*" OR "Parent-child*" OR intergeneration* OR “mother-adolescent*” OR “father-adolescent*” OR “parent-adolescent*”) Near/3 (relation* OR routine* OR function OR functioning OR cohesion OR "social environment" OR interaction OR interactions OR organization OR "affective environment" OR "problem solving" OR communication OR communications OR involvement OR closeness OR dynamic OR dynamics OR chaos OR disorder OR "verbal behavior*" OR adaptability OR "cooperative behavior" OR "interpersonal relations*" OR warmth OR "affective expression" OR "affective involvement" OR connectedness))

**Child**

Title, Abstract, Author Keywords, Keywords Plus: (("Child*" OR "Preschool*" OR "Adolescen*" OR "Pediatric*" OR "Paediatric" OR "Youth*" OR "Elementary student*" OR "High school student*" OR "elementary school student*" OR teenage* OR girl OR boy OR girls OR boys OR "schoolgirl*" OR "schoolboy*" OR "schoolchild*" OR "schoolkid*" OR kid OR kids OR “young athlete*” OR “grade level” OR “kindergarten student*” OR “middle school student*” OR “intermediate school student*” ))

**Physical Activity**

Title, Abstract, Author Keywords, Keywords Plus: (((athletic OR motor OR physical OR aerobic OR anaerobic) Near/3 (behavior OR activit* OR skill OR skills OR control OR performance OR function OR functioning OR fitness OR exercise OR training OR conditioning OR health OR education OR literacy OR recreation OR program*)) OR bicycling OR cycling OR swimming OR tobogganing OR "winter play" OR "park play" OR walking OR skating OR basketball OR "home exercise" OR "weight training" OR trampoline OR "skipping rope" OR "jump rope" OR "indoor chores" OR "outdoor chores" OR tag OR baseball OR softball OR football OR soccer OR volleyball OR dance OR handball OR "mini handball" OR ball-playing OR "street hockey" OR "floor hockey" OR "ice hockey" OR ringette OR "ice skating" OR "in-line skating" OR skateboarding OR "track and field" OR jogging OR running OR golf OR gymnastics OR yoga OR "exercise class" OR badminton OR tennis OR kayaking OR canoeing OR "downhill skiing" OR snowboarding OR skiing OR boxing OR wrestling OR karate OR judo OR “tai-chi” OR "tae kwon do" OR "cross country skiing" OR “cycling” OR sport OR sports)

**Scopus (Elsevier)**

**Family Functioning**

Title, Abstract, Keywords: ((family OR families OR "Mother-child*" OR "Father-child*" OR "Parent-child*" OR intergeneration* OR “mother-adolescent*” OR “father-adolescent*” OR “parent-adolescent*”) W/3 (relation* OR routine* OR function OR functioning OR cohesion OR "social environment" OR interaction OR interactions OR organization OR "affective environment" OR "problem-solving" OR communication OR communications OR involvement OR closeness OR dynamic OR dynamics OR chaos OR disorder OR "verbal behavior*" OR adaptability OR "cooperative behavior" OR "interpersonal relations*" OR warmth OR "affective expression" OR "affective involvement" OR connectedness))

**Child**

Title, Abstract, Keywords: (("Child*" OR "Preschool*" OR "Adolescen*" OR "Pediatric*" OR "Paediatric" OR "Youth*" OR "Elementary student*" OR "High school student*" OR "elementary school student*" OR teenage* OR girl OR boy OR "schoolgirl*" OR "schoolboy*" OR "schoolchild*" OR "schoolkid*" OR kid OR kids OR “young athlete*” OR “grade level” OR “kindergarten student*” OR “middle school student*” OR “intermediate school student*” ))

**Physical Activity**

Title, Abstract, Keywords: ((((athletic OR motor OR physical OR aerobic OR anaerobic) W/3 (behavior OR activit* OR skill OR skills OR control OR performance OR function OR functioning OR fitness OR exercise OR training OR conditioning OR health OR education OR literacy OR recreation OR program*)) OR bicycling OR cycling OR swimming OR tobogganing OR "winter play" OR "park play" OR walking OR skating OR basketball OR "home exercise" OR "weight training" OR trampoline OR "skipping rope" OR "jump rope" OR "indoor chores" OR "outdoor chores" OR tag OR baseball OR softball OR football OR soccer OR volleyball OR dance OR handball OR "mini handball" OR ball-playing OR "street hockey" OR "floor hockey" OR "ice hockey" OR ringette OR "ice skating" OR "in-line skating" OR skateboarding OR "track and field" OR jogging OR running OR golf OR gymnastics OR yoga OR "exercise class" OR badminton OR tennis OR kayaking OR canoeing OR "downhill skiing" OR snowboarding OR skiing OR boxing OR wrestling OR karate OR judo OR “tai-chi” OR "tae kwon do" OR "cross country skiing" OR “cycling” OR sport OR sports))
