# Supplementary material for: Association between child and youth physical activity and family functioning: a systematic review of observational studies
Source: Int J Behav Nutr Phys Act. 2025 Jul 22;22:101. doi: 10.1186/s12966-025-01782-z (PMC12281683; doi:10.1186/s12966-025-01782-z)
Supplement: Supplementary file 2 — Supplementary Material 2. [file 12966_2025_1782_MOESM2_ESM.docx]

**Appendix B**

**Further Analysis for Thematic Classification**

To execute thematic classification, effect sizes, and significance values directly measuring the relationship between family functioning and child physical activity were extracted.^42^ Median effect sizes and the interquartile range were prioritized during the analysis. As several studies presented multiple effect sizes the authors established a hierarchy of effect sizes. Firstly, as the authors were most interested in a bidirectional relationship, the Pearson correlation coefficient (*r*) was prioritized for median and interquartile range calculations. Secondly, for studies that presented multiple effect sizes based on different types/measurement devices of physical activity, moderate to vigorous measures of physical activity, and device-measured measurement (i.e., accelerometry) were prioritized. Moderate to vigorous physical activity was chosen as it is the intensity type described in the Canadian 24-hour movement guidelines for children and youth aged 5 to 17 years.^73^ Device measurement of physical activity was chosen as it has been shown to provide a more accurate representation of a child’s movement behaviors.^32^ When multiple effect sizes based on different measurements of family functioning were presented, parental self-report of family functioning was prioritized. Parental self-reporting has been shown to provide a more accurate picture of a family’s functioning compared to children’s reports, which can be more susceptible to variance.^22^ Lastly, an aggregate of values was taken for studies that presented separate effect sizes and significance levels for males and females, children and youth, or mothers and fathers.
