# Supplementary material for: Association between child and youth physical activity and family functioning: a systematic review of observational studies
Source: Int J Behav Nutr Phys Act. 2025 Jul 22;22:101. doi: 10.1186/s12966-025-01782-z (PMC12281683; doi:10.1186/s12966-025-01782-z)
Supplement: Supplementary file 3 — Supplementary Material 3. [file 12966_2025_1782_MOESM3_ESM.docx]

**Appendix C**

**Detailed Extracted Results**

| **Title of Article** | **Author(s) and Year of Publication** | **Country** | **Sample Population (Mean Number of Participants, Characteristics (e.g., ethnicity, SES, employment status of parent, age of child (mean and standard deviation)) . Please Describe the child demographics - disregard parent age, or any other defining facors of parent that do not directly impact the child.** | **Family Unit Description (e.g., mother-child, father child, parents-child) and number of family members within the household unit.** | **Type of Observational Study Design and Timeframe** | **Study Methods (e.g., Data Collection Methods, etc.)** | **If obtained via a subset of a prior study cohort please indicate; if not mark N/A** | **Physical Activity Reported (self reported, accelerometry, pedometry, etc.), and any additional description of PA measure.** | **Physical Activity Results** | **Family Functioning Measure** | **Domain of Family Functioning Examined** | **Report of Cohesion** | **Report of Communication** | **Report of Conflict** | **Report of Organization** | **Report of Affective Environment** | **Report of Family Problem Solving Ability** | **Report of Household Chaos** | **Report of General Family Functioning (i.e., Family relationships)** | **Transformed Effect Size Results** | **Prioritized Effect Size** |
| --- | --- | --- | --- | --- | --- | --- | --- | --- | --- | --- | --- | --- | --- | --- | --- | --- | --- | --- | --- | --- | --- |
| Family Cohesion and Moderate-to-Vigorous Physical Activity Among Mexican Origin Adolescents: A Longitudinal Perspective | Bigam, Rajesh, Koehly, Strong, Oluyomi, Strom, Wilkinson (2015) | USA | Participant (N = 711). Female (N = 403) , Male (N=308). Age(years): <13 = 179, 14 = 232, 15 = 216, >16 = 84. Mean(SD) Age (Years) = 14.3(1.1). | Mexican adolescents reported their physical activity. (parent-child dyad). | Longitudinal study design from years 2008-2011. | Data collected via personal interview (at home) in 3 waves: baseline (2005-06 - participants between the ages of 11-13), follow up 1 (2008-09), and follow up 2 (2010-2011). 5 minute interview - collecting demographic information. Following participants completed a survey on a personal digital assistant. Questions pertaining to PA were administered on follow ups 1 and 2. | Data obtained from participants enrolled in a prospective cohort study of smoking behaviour that began in 2005. Participants drawn from a population-based cohort of Mexican American households launched in 2001 by the department of Epidemiology at the university of Texas. The name of the cohort: Mano-a-Mano Mexican American Cohort Study | *Self-report:* Moderate to vigorous PA using the Youth Risk Behaviour Surveillance System (YRBSS). YRBSS asks "during the past 7 days, on how many days were you physically active for a total of at least 60 min per day) - proceed to add up all time spent in any kind of PA that increased HR and breathe heavier. Participants reporting to be physically active for 60 minutes a day on at least 5/7 days were coded 1 (adequate completion of MVPA) - anything less = 0. 0 was lableed as "sedentary and light behaviour". | 28.4% reported adequate MVPA - higher prevalence among boys (34.5%), girls (24.0%). | Family cohesion and conflict were assessed using 2 subscales - the Family Life Questionnaire (Foxcroft & Lowe, 1995) | *Cohesion and Conflict* | Crude and Adjusted Relative Risks for Physical Activity (SLPB vs. MVPA) in 2010-2011 Among Participants Who Completed Inadequate MVPA in 2008-09: Crude RR (N = 711) = RR = 1.32, 95%CI = 1.04-1.68, p = 0.023. Full Model (N = 706) RR = 1.32, 95%CI = 1.06-1.64, p=0.012. Reduced Model (N = 706) RR = 1.32, 95%CI = 1.07-1.63, p=0.010. | NA | Crude and Adjusted Relative Risks for Physical Activity (SLPB vs. MVPA) in 2010-2011 Among Participants Who Completed Inadequate MVPA in 2008-09: Crude RR (N = 711) = RR = 1.07, 95%CI = 0.87-1.32, p = 0.528. Full Model (N = 706) RR = 0.91, 95%CI = 0.74-1.13, p=0.410. Reduced Model (N = 706) RR = 0.91, 95%CI = 0.74-1.63, p=0.408. | NA | NA | NA | NA | N/A | COHESION: Crude and Adjusted Relative Risks for Physical Activity (SLPB vs. MVPA) in 2010-2011 Among Participants Who Completed Inadequate MVPA in 2008-09: Crude RR (N = 711) = RR = 1.32, z' = 0.076. Full Model RR= 1.32, z' = 0.076 Reduced Model RR = 1.32, z' = 0.076.  CONFLICT: Crude and Adjusted Relative Risks for Physical Activity (SLPB vs. MVPA) in 2010-2011 Among Participants Who Completed Inadequate MVPA in 2008-09: Crude RR = 1.07, z' = 0.019. Full Model RR = 0.91, z' = -0.026. Reduced Model) RR = 0.91, z' = -0.026. | Cohesion: Crude z' = 0.076. positive, significant. Conflict: Crude z' = 0.019. Positive, not significant. |
| The Association Between Physical Activity and General Life Satisfaction in Lower Secondary School Students: The Role of Individual and Family Factors | Kleszcezewska, Dzielska, Salonna, Mazur (2018) | Czech Republic | N = 4085 (boys = 42%, girls = 52%). Class of secondary school: I = 32.9%, II = 35.6%, III = 31.5%. | Not disclosed. | Cross sectional observational study | Random sampling through computer-assisted web survey. Questions of the survey consisted of Student life satisfaction scale (dependent variable), the family affluence scale, the vigorous physical activity indicator, the self-esteem scale, the scale of the perception of family relations, and the assessment of school performance were used. | N/A | *Self-report:* Vigorous physical activity was based on one question "how often, in your free time, out of your school classes, do you engage in physical exercise during which your physical effort is great, i.e., you feel that you are short of breath and sweating? There were seven categories of answers. VPA indictor was used in the analyses as a quasi-continuous variable (a range of 0-6 points), divided into five categories: rarely or never; once a week 2-3 times a week, 4-6 times a week; everyday. | rarely or never = 13.7% (N=545/1907), 1 time per week = 12.8% (N=512/1907), 2-3 times per week = 29.1% (N=1160/1907), 4-6 times per week = 20.9% (N=834/1907), everyday = 23.5% (N=935/1907). | the scale of the perception of family relations. Young people described how often in the last 4 weeks they experienced positive feelings in family relations. | *General Family Functioning (i.e., family relationship)* | N/A | N/A | N/A | N/A | N/A | N/A | N/A | Correlation matrix: family relationship = 0.058 (weak positive correlation between family relationships and physical activity) - not a very strong relationship. | r = 0.058, z' = 0.058. | General FF: I think positive significant w/ 0.058. |
| Exercise is positively related to adolescents' relationships and academics | Field, Diego, Sanders (2001) | USA | 89 suburban high school seniors (female N = 52, male N = 37). On average, they were of middle to upper middle SES (M = 3.9 on the Hollingshead Two-Factor Index). Ethnicity: 76% = Caucasian, 11% = Hispanic, 5% = Asian, 3% = African-American, 5% = other | Not disclosed. | Cross-sectional observational study | Participants completed a 181-item Likert-type questionnaire on the behavioral and psychological aspects of adolescent life. | N/A | *Self-report:* Exercise was assessed on a 5-pt Likert scale: rarely (1), sometimes (2), once a week (3), three or more times a week (4), daily (5). | median split on the exercise variable. High level of exercise = 36 adolescents (N female = 23, N male = 13). Low group = 53 adolescents (N female = 29, N males = 24). | Quality of relationships with parents/friends was assessed on a 5-pt Likert scale, ranging from not at all, to very much. Exp: "how much does your mother accept you no matter what you do?". Intimacy with parents (frequency of personal conversations, doing fun things together), touch (showing physical affection to parents or touching), (receiving physical affection from parents or touching), and family support (closeness to siblings and other relatives). | *Cohesion and General Family Functioning (I.e., family relationship)* | Mean Scores and Significant Group Differences: Intimacy with parents and high exercise group (N = 36): 16.1, p = 0.05. Intimacy with parents and low exercise group (N =53): 14.9, p=0.05. | N/A | N/A | N/A | N/A | N/A | N/A | The analyses suggested that adolescents in the high-exercise group had better relationships with their parents than did those in the low-exercise group. The high-exercise group reported higher quality relationships with parents. F(1,68) = 4.23, p<0.05. Mean Scores and Significant Group Differences: Quality of relationships with parents and high exercise group (N = 36) = 33.6, p = 0.05. Quality of relationships with parents and low-exercise group = 30.9, p=0.05. | N/A | Reported just p-values - significant positive? |
| Attachment relationships and physical activity in adolescents: The mediation role of physical self-concept | Li, Bunke, Psouni, 2015 | Sweden | 783 Chinese adolescents (49% male) between 11 and 15 years old (M = 12.92, years, SD = 0.86). | Not disclosed. | Cross-sectional observational study | Informed consent was obtained from parents, and verbal consent was obtained from students. Questionnaire administered to the students during class time. | N/A | *Self-report:* Participants regularly attended PE classes 2-3 times. Self-report PA was measured with the Physical Activity Questionnaire for Adolescents (PAQ-Q; Koqalski, Crocker, & Donen, 2004). It is a 7-day recall questionnaire with 9-items (e.g. "in the last 7 days, during your physical education classes, how often were you very active (playing hard, running, jumping, throwing)?". Responses were on a 5-point scale with higher scores indicating higher levels of PA. | PA range (1-5), M male (SD) = 2.33 (.63), M female (SD) = 2.02 (.54), M total (SD) = 2.17. (.60) | Attachment relationships with parents and friends were measured with the Inventory of Parent and Peer Attachment (Chinese version). 25 items, repeated for each attachment relationship (mother, father and peer). 3 dimensions trust (attachment figures understand and support one's needs and desires), communication (attachment figures are sensitive and responsive to one's emotional states (i like to get my father's view on things I'm worried about) and alienation (avoidance when I talk about my problems with my best friend). Responses are on 5 pt scale from almost never or never true to almost always or always true. | *Cohesion* | Significant direct effect was revealed between father attachment security and PA (β = .15, p<0.1). First Order Factors Models: attachment security in relation to father was only linked to PA directly (β = .13, p<0.05). The parsimonious higher-order model describing mediation of physical self-perception in the link between attachment security and PA: Father attachment and PA: β= .16. (PA was also found to be directly linked to father attachment (β = .16, p<0.01). The parsimonious first-order model describing mediation of the four subdomains of physical self-perception in the link between attachment security and PA: Father attachment and PA: β = .14. Attachment security in relation to mother and father was positively linked to PA (β = .12, p<0.05; β = 0.09, p<0.05). Gender Differences: path from father attachment to PA significant for males (γ = .20, p<0.05) but not females (γ=0.11, p>0.05). Descriptive statistics and pearson correlations for adolescent males (N = 375) and females (N = 369), and total sample (N =767) Security Attachment to Mother and PA = .17 (P<0.001). Security Attachment to Father and PA = .22 (P<0.001). | N/A | N/A | N/A | N/A | N/A | N/A | N/A | Father cohesion and PA = β = .15, z' = 0.151. First Order Factors Models: cohesion and PA β = .13, z' = 0.131. Parsimonious Higher-Order Model Describing Mediation of physical self-perception in the link between attachment security and PA: father cohesion and PA, β = .16, z' = 0.161. Parsimonious first-order model describing mediation of the four subdomains of physical self-perception in the link between attachment security and PA: father cohesion and PA: β = .14, z' = 0.141. mother and father cohesion and PA = β = .12, z' = .121, β = 0.09, z' = 0.090. Pearson Correlations for Adolescents cohesion to mother and PA r = 0.17, z' = 0.172, Father r = .22, z' = .224 | Cohesion: Used r values (took average of converted z' for mother and father) = 0.198. positive, significant. |
| Factors associated with family function in school children: Case-control study | Botero-Carvajal, Jimenez Urrego, Gutierrez-Posso, Calero-Florez, Hernandez-Carrillo (2023) | Columbia | Participants N = 1141 students in grades 6 to 11. Ages 10 to 18. Cases M&F = 57, Controls = 233. | Not disclosed. | analytical case-control study | family APGAR questionnaire was filled out by participants and a score was recorded out of 20. Based on the score, the researchers defined the children as either cases or controls (normal family function: 17-20 pts, mild dysfunction: 16-13pts, moderate dysfunction: 12-10 pts, severe dysfunction: 9pts or less). Children scoring less than or equal to 16 pts as cases, and children with scores greater than 16 points as controls. 1:1 analysis was conducted, paired by the municipality. Researchers used an analytical approach of paired cases and controls, the raw and adjusted Odds Ratio (OR) were calculated (using conditioned logistic regression). | study population came from the secondary database provided by the departmental health secretariat, which is made up of school children enrolled in grades 6 to 11, who attended official educational institutions in the municipalities of Candelarai, Florida, Jamundi, and Pradera, Valle del Cauca, Columbia | *Self-report:* self report "do some sport at least once a week). Method of quantifying results was not disclosed. | N/A | A questionnaires was applied that contained questions about the family relationship and the social environment of the schoolchild. The APGAR scale was used to identify family dysfunctions. Cronbach's alpha for the family APGAR was 0.90 in Columbia, suggesting that it represents unidimensional construct validity: family function. The items of the scale are scored as never, rarely, sometimes, almost always, and always. Total score range from 0-20; the higher the score, the better the family functionality. The family relationship was measured using dichotomous questions, about living with their parents, free time shared with them,  and talking with them daily; in addition, the physical and verbal punishment that was administered in the upbringing of the  schoolchild was investigated. | *Conflict* | N/A | N/A | Multivariate analysis using conditional logistic regression Factors associated with family dysfunction in school children enrolled in grades 6 to 11 in four municipalities in Valle del Cauca, Columbia Year 2009: Family dysfunction and "Do some sport at least once a week": OR = 0.42, p-value = 0.029 (p<0.05), I-C 95% = 0.20-0.91. | N/A | N/A | N/A | N/A | N/A | CONFLICT: Transformed Fischer z (z') = -0.237 for conflict (using OR = .42). | Conflict: Significant,negative, -0.237 |
| Internal versus external predictors of physical activity in youth | Ryan, Kaskas (2022) | USA | N = 5672. Participants’ ages ranged from 12 to 18 years (M = 15.92 years, SD = 1.64. Eighty-eight percent of participants self-identified as Non- Hispanic/Latino, 66% identified as Caucasian, 24%  identified as African American, 1% identified as Native  American, 4% identified as Asian, and 5% identified as  other. Fifty-one percent were female. Socio-economic status  was also collected; the mean annual income for participant  families was $47,700 (SD = $56,355). | Mother-child dyad - no further information disclosed. | Cross sectional observational study | Participants selected from the first wave of longitudinal data in the Add Health study. All data included in this study were included in the Wave I In-Home Interview Questionnaires completed by the adolescent. The instructions that were read to participants prior  to completing interviews. PA was measured along with planned covariates such as physical limitations and screen time Measures of predictor variables were also recorded, including items such as depressive and anxious symptoms, self-esteem, frequency of pain, BMI, time with friends, mother-child relationship, and risky behaviors. | Participants were selected from the first wave of longitudinal data in the national longitudinal study of adolescent health. The total add health database comprises a sample of over 10,000 adolescents from 80 high schools and 52 middle schools in the USA in 1994 to 1995 for wave I | *Self-report:* Self reported frequency of daily PA over the past week, using three items from a health questionnaire with a 4-pt Likert scale. The first item inquired about frequency per week of rollerblading, biking, skate-boarding, and roller-skating; the second item inquired about frequency of active sports (e.g., baseball, softball, basketball, soccer, swimming, football); and the third item inquired about frequency of exercise (e.g., jogging, walking, karate, jumping rope, gymnastics or dancing). Categorized youth into groups based off of nunbre of times per week they spent engaged in any form of PA. low (sum of PA frequency items 0-2), medium (sum of PA frequency items 3-4), and high (sum of PA frequency 5 or more). | 1,857 participants (i.e., 33%) were in the low group, 1,901 participants were in the medium group (i.e., 33%), and 1,914 participants were in the high group (i.e., 34%). Sum of PA items= M 3.66 , SD 2.13 | Aspects of mother-child relationship assessed through the sum of items such as: “Your mother is warm and loving towards you,” and “You are satisfied with how you and your mother communicate with each other.” The five items were coded on a 5-point Likert scale ranging from 1 (strongly agree) to 5 (strongly disagree) | *General Family Functioning (i.e., family relationship)* | N/A | N/A | N/A | N/A | N/A | N/A | N/A | Parameters of the Full Ordinal Logistic Regression Model: Physical activity and mother-child relationship: B = 0.004, Std. Error = 0.015, Wald Chi-Square = 0.066, p-value = 0.053. Odds Ratios of Full Ordinal Logistic Regression Model: Physical activity and mother-child relationship: Exp(B) = 1.004, Lower 95% OR = 0.975, Upper 95% OR = 1.033 (not significant) | Transformed Fischer z (z') = 0.009 for general FF (using OR = 1.033). | General FF: not significant, positive, 0.009 |
| An Investigation of Youth Assets and Physical Activity and BMI Using a Longitudinal Cohort Design | Oman, Clements-Noelle, Lu, Lensch (2018) | USA | N - 1111 parent and youth dyads. residents living in randomly samples census tracts located in Oklahoma city metropolitan area. mean age = 14.3 years (SD = 1.6); 53% female;  40.6% white, 28.6% Hispanic, 24.4% black, and 6.4% other.  Family yearly income was <$35 K (47%), $35K-$62 K (30%),  and >$62 K (23%) | parent and youth dyads. | longitudinal cohort | 5 waves of data were collected over a 4-year period from youth and their parents. Participants were interviewed annually in their homes in person using a computer-assisted interviewing and data entry system. Fourteen youth assets were assessed via multi-item constructs. Five assets represented strengths at the individual level, 4 at the family level, and 5 at the community level. The asset scale included 14 constructs. | N/A | *Self-report:* PA was assessed via the Godin Leisure-Time Exercise Questionnaire that assesses minutes of participation in strenuous (e.g., running), moderate (e.g., fast walking), and light (e.g., easy walking) intensity PA during a typical week. A weekly PA score was calculated by summing the total number of weekly minutes that the youth engaged in strenuous or moderate intensity PA. Weekly minutes engaged in light intensity activities were excluded from the PA score. | The baseline mean number of minutes per  week that youth reported participating in vigorous or moderate-intensity PA was 133 minutes (SD = 55, range = 30-240). | Fourteen youth assets were assessed with multi-item constructs, including four family level assets - based on a score from 1 (almost never/strongly etc.) to 4 (almost always/strongly agree, etc.) | *Communication* | N/A | Results of Generalized Linear Mixed Models Analyses of Youth Asset Groups on Youth Minutes of Weekly Physical Activity and BMI: PA minutes and family level assets: 2 vs 0-1: coefficient = 5.90, 95% CI = -0.04 to 11.85, p-value = 0.052. 3 vs. 0-1: coefficient = 8.65, 95%CI = 4.02 to 14.79, pvalue = 0.003. 4vs. 0-1: coefficient = 13.88, 95% CI = 11.80 to 23.32, pvalue <0.0001. Associations between differences in number of youth assets and youth weekly number of minutes in physical activity: Mean diff weekly mins of PA and 0-1 vs. 3 assets = 5.9. Mean diff weekly mins of PA and 0-1 vs. 4 assets = 8.65. Mean diff weekly mins of PA and 0-1 vs. 5 assets = 13.88. | N/A | N/A | N/A | N/A | N/A | N/A | COMMUNICATION: Results of Generalized Linear Mixed Models Analyses of Youth Asset Groups on Youth Minutes of Weekly Physical Activity and BMI: PA minutes and family level assets: 2 vs 0-1: OR = 5.90, z' = 0.472. 3 vs. 0-1: OR = 8.65, z' = 0.564. 4vs. 0-1: OR = 13.88, z' = 0.673. | Communication: taken average of all family-level assets z' values = 0.5667. Significant positive effect. |
| High school athletic participation, sexual behavior and adolescent pregnancy: a regional study | Sabo, Miller, Farrell, Melnick, Barnes (1999) | USA | Wave 3 (N = 612). 55% = female, 33% = black. Thirty-eight percent were single-parent households. Mothers’ average education was 13.2 years | family composition: mothers, fathers, and adolescent siblings (aged 13-18 years). | longitudinal observational study | data from previously collected longitudinal study of a random population sample of 699 families with adolescents ages 13 to 16 years living in households in a northeastern metropolitan area was used. Sampling was done by means of random-digit-dial procedures on a computer-assisted telephone network. In home interviews with trained interviewers was conducted along with self administered questionnaires completed by both adolescent and parent. | Previously collected data from the longitudinal study of a random population sample of 699 families with adolescents ages 13 to 16 | *Self-report:* Athletic participation was measured through a questionnaire related to participation in sports at school. | not disclosed. | *Cohesion:* measure of family cohesion: Olson, Portner, and Lavee's 1985 FACES III scale. 6-pt Likert scale ranged from "almost never" to "almost always" | *Cohesion* | Cohesion & Sports: Boys Standardized Regression Coefficient = 0.14, p<0.05. Girls not reported - as not significant? In separate models for boys and girls, a path analysis was used to examine the direct and indirect effects of participation in sports on frequency of sexual intercourse and involvement of pregnancy - in both models, the exogenous control variables were family cohesion. Just boys have higher participation in sports with higher levels of family cohesion | N/A | N/A | N/A | N/A | N/A | N/A | N/A | Cohesion: β = .14, z' = 0.141 | Cohesion: positive, significant, 0.141 |
| Cross-sectional and prospective associations between children’s 24-h time use and their health-related quality of life: a compositional isotemporal substitution approach | Tan, Padmapriya, Bernard, Toh, Wee, Tan, Peng Yap, Lee, Chong, Godfrey, Eriksson, Pei-Chi Shek, Sheng Tan, Chong, Muller-Riemenschneider (2023) | Singapore | 836 initial participants; 711 participants at follow up ages 8 and 10 years. 370 participants had valid accelerometer data at both time points and completed the KINDL-Kid questionnaire. N = 370 participants included for analysis. Boys = 183 (49.5%), Girls = 187 (50.5%). Ethnicity: Chinese = 216(58.4%), Malay = 201 (27.3%), Indian = 53 (14.3%). Maternal education: no education/primary/secondary = 112 (30.3%), Post-secondary = 77 (20.8%), degree and above = 181 (48.9%). | Mother-child dyad - no further information disclosed. | longitudinal observational study | GUSTO cohort study started in 2009. Pregnant women recruited from public maternity units in Singapore. SES collected from medical records at birth of child or via follow-up interview. BMI was recorded based on anthropometric measures at 8 year visit. At the 8-10 year follow up, participants movement behaviour was gathered via triaxial accelerometer. Health related quality of life was collected via the KINDL-Kid questionnaire. Data analysis was performed using multivariable linear regression models; results presented as β(95% CI). | Child participants part of the GUSTO mother-offspring cohort | *Device-measured:* Triaxial accelerometer (ActiGraph wGT3X + BT). Accelerometer removed on the 9th day - this allowed for 7 complete days of continuous 24-hour data capture. | light PA h/day = M = 5 SD = 0.9. MVPA min/day = M = 71.5 SD = 28.6 | *General family functioning:* Part of HRQoL score from KINDL-Kid - extracted out relationship with family at 10 years of age. | *General Family Functioning (i.e., family relationship)* | N/A | N/A | N/A | N/A | N/A | N/A | N/A | We found no cross-sectional association between movement behaviours and overall HRQol, or with the domains of the HRQoL relating to relationship with family. Compositional time use at age 10 years and its association with HRQoL: Relationship with family and LPA: β = 5.11 (−5.03, 15.26) (p = 0.322). Relationship with family and MVPA: β = 0.09 (−5.32, 5.49) (p = 0.975).  Compositional time use at age 8 years and its association with HRQoL: relationship with family and LPA: β = −0.67 (−12.30, 10.96) (p = 0.910). relationship with family and MVPA: β = −2.93 (−9.00, 3.13) (p = 0.342) | General FF: β = 0.09, z' = 0.09. | General FF: Used specifically just MVPA (standard for this study): z' = 0.09. Positive, not significant. |
| Adolescents' reports of chaos within the family home environment: Investigating associations with lifestyle behaviours and obesity | Van Hulst, Jayanetti, Sanson-Rosas, Harbec, Kakinami, Barnett, Henderson (2023) | USA | N = 377. Age: Overall = 16.8 ± 1.0, Boys = 16.8 ± 0.9, Girls = 16.8 ± 1.0. Sex: Boys = 54.1% (n=204), Girls = 45.9% (n = 173). Household income (Canadian $): Full Sample = 57 040 ± 24 491, Boys = 56 692 ± 23 709, girls = 57 448 ± 25 440. Parental education: 1 or 2 parents with university degree: Full sample = 55.7 (210), Boys = 55.9 (114), Girls = 55.5 (96). Both parents with less than a university degree: full sample = 44.0 (166), boys = 43.6 (89), 44.5 (77). | Family structure (lives with both parents): full sample = 74.5 (281), boys = 75.0 (153). Girls = 74.0 (128) | Cross sectional | Data obtained from QUALITY cohort, an ongoing longitudinal investigation on obesity and CV risk factors among children 8-10 years at baseline. Recruited from elementary schools located in 3 major urban centers in Quebec. Participants were required to be white and both parents to be available to participate at baseline w/ at least one parent having obesity. Two follow-up assessments were conducted at ages 10-12 and 15-17 years. Data collection occurred between 2012 to 2016. Household chaos, BMI, MVPA, sleep duration, screen time, dietary intake was collected. Multiple linear regression models were used to estimate associations between each dependent variable (zBMI, MVPA, sleep duration, screen time, and fruit and vegetable servings), and the primary independent variable (CHAOS). | Cross-sectional data of the 3rd wave of data collection from the Quebec Adipose and Lifestyle Investigation in Youth (QUALITY) cohort study were analysed | *Device-measured:* MVPA was assessed with an Actigraph monitor (Triaxial, GT3X). Participants instructed to wear the monitor for 7 days following the research visit. The data were included only for those participants who wore the activity monitor for at least 4 days and at least 10 hours per day. MVPA was calculated by adding the total number of minutes of daily MVPA per day averaged over the total valid days of wear. | MVPA (mins/day) = 24.2 (13.6, 37.4). Boys = 29.9 (17.6, 42.5). Girls = 17.7 (11.4, 30.3) | *Chaos:* Household chaos was self-reported by adolescents using the Confusion, Hubbub, and Order Scale (CHAOS). Adolescents were asked to score the 15 CHAOS items using 5-pt Likert scales ranging from definitely false to definitely true. | *Household Chaos* | N/A | N/A | N/A | N/A | N/A | N/A | Association (Beta, 95% CI) between household chaos, lifestyle behaviours and zBMI, in the full sample and stratified by sex, QUALITY cohort study, visit 3(n=377): Model A (full sample): CHAOS continuous and MVPA = 1.42 (-0.15, 2.98). Model B (full sample): CHAOS higher vs. lower and MVPA = 6.57 (-1.65, 14,79). Girls (n = 173): Model A: CHAOS continuous: 1.35 (-0.73, 3.44). Model B: CHAOS higher vs. lower = 6.11 (-5.21, 17.42). Boys (n=204): Model A: CHAOS continuous: 1.56 (-0.82, 3.94. Model B: CHAOS higher vs. lower = 9.27 (-3.22, 21.77) | N/A | N/A - study used non standardized Beta values, thus unable to transform to z'. | Household Chaos: positive, not significant for the p-value table. |
| Utilizing a Board Game to Measure Family/Parenting Factors and Childhood Obesity Risk | Berge, Telke, Tate, Troflholz (2019) | USA | child n = 150. female = n = 71(41%). age = M = 6.4, SD = 0.8. Race/ethncity:American Indian or Alaskan Native parent = 21 (14) child = 25 (17) Asian parent = 25 (17) child = 25 (17), Black or African American parent = 22 (15) child = 25 (17), White parent = 27 (18) child = 25 (17), Somali parent = 25 (17) child = 25 (17), Hispanic parent = 23 (15) child = 25 (17), Mixed/other parent = 7 (5). Educational attainment (parent): Middle school or junior high 15 (10), Some high school 17 (11), High school or General Equivalency, Diploma 60 (40), Vocational, technical, trade certificate program, or associate degree 28 (18), College degree (eg, bachelors, masters, or doctorate) 25 (16), Other 4 (3), Missing 1 (1). Primary caregiver work status: Working full time 63 (42), Working part time 32 (21), Stay at home caregiver 25 (17), Currently unemployed, seeking work 18 (12), Not working for pay (unable to work, retired, student, etc) 11 (7), Not applicable 1 (1). Household income: <$20,000 50 (33), $20,000 to $34,999 55 (37), $35,000 to $49,999 16 (11), $50,000 to $74,999 12 (8), $75,000 to $99,999 7 (5), ≥$100,000 9 (6), Missing 1 (1) | Household structure: 1 parent (no other adults = n = 37 (25%), 1 parent (with other adults) = n = 18 (12%), 2 parents (no other adults) = n = 78(52%), 2 parents (with other adults) = n = 17 (11%). Primary caregiver marital status: Married 78 (51), Committed dating relationship or engaged 31 (21), Casually dating 2 (1), Separated or divorced 6 (4), Widowed 1 (1), Single/never married 31 (21), Missing 1 (1) | Mixed methods, cross-sectional | Data was used from the phase I of the Family Matters study. Mixed methods analysis was conducted of the home environments of children aged 5-7 years from 6 racial and ethnic groups (African American, American Indian, Hispanic/Latino, Hmong, Somali, and white). Aim was to identify familial risk and protective factors for childhood obesity. Two home visits 10 days apart, conducted by 2 trained research staff matching the cultural background of the participant. Data collected occurred in the participant's home. Interactive family board game task was recorded. Families engaged in this task on the first home visit. Game took between 20-50 minutes to complete. Exposure variables were recorded using the Iowa Family Interaction Rating Scale. Blinded and trained individuals were responsible for coding the video recorded of the family interactive task. Child weight status, anthropometric measures, child overall diet quality and PA and sedentary activity was measured. Race/ethnicity and age were assessed as covariates. Descriptive analyses were performed to describe the sample and evaluate modeling assumptions. Adjusted logistic and linear regression models w/ robust SEs were used to examine whether dyadic interactions across all family members were associated with child overweight status, diet quality, PA and sedentary behaviour. logistic and linear regression models w/ robust SEs were used to examine specific parent-child, and parent-sibling interactions. | Data for the current study were from Family Matters, 21 a 5-year incremental (phase I was 2014−2016; phase II was 2017−2019), mixed-methods (e.g., video-recorded tasks, ecological momentary assessment, interviews, surveys) longitudinal study designed to identify novel risk and protective factors for childhood obesity in the home environments of racially and ethnically diverse, primarily low income children. | *Device-measured:* Child PA was measured by accelerometer to capture participant frequency and intensity of motion. Participants instructed to wear the device for at least 4 days for 8 hours/day, including at least 1 weekend day. | not disclosed. | *Cohesion, communication, conflict:* Iowa Family Interaction Rating Scale was used to code video-recorded family interactive task. Includes measures such as warmth/support (cohesion), communication, and hostility (conflict) | *Cohesion, Communication, Affective Environment and General Family Functioning (i.e., family relationship)* | There was a significant positive association (p < 0.05) between engaging in warm/supportive behaviors and the child engaging in more hours of MVPA for 3 of the 6 dyads. Primary caregiver to study child warmth/support and MVPA = Mean difference = 11.87 95% CI= (4.99 to 18.75) p-value =.001. Study child to primary caregiver warmth/support and MVPA = Mean difference = 3.30 95% CI= (−3.19 to 9.78) p-value = .32 | Primary caregiver to study child communication to MVPA = mean difference = 1.53, 95% CI = (-7.9 to 10.96), p-value = 0.75. study child to primary caregiver communication to MVPA = mean difference 4.90, 95%CI = (−1.82 to 11.61), p-value=.15 | N/A | N/A | primary caregiver to study child hostility and MVPA = mean difference = −3.67 95%CI = (−13.29 to 5.95) p-value = .45. study child to primary caregiver and MVPA = mean difference = −10.16, 95% CI = (−19.33 to −0.99), p-value = .03 | N/A | N/A | In addition, for primary caregiver−child and primary caregiver−sibling dyads, there was a significant positive association between relationship quality and the child engaging in more hours of MVPA (P < .05). There was no significant associations between other FF behaviours including warmth/support or communication and child PA for any of the 6 family dyads. | N/A - no standard deviation reported, thus unable to convert to cohen's d. | Cohesion: significant positive. Communication: positive, not significant. Affective Environment: positive not significant. General FF: significant positive. |
| Association of Family Functioning on Youth Physical Activity and Sedentary Behavior | Loprinzi (2015) | USA | NSCH 2003: N = 61,226. Child Age ( M = 11.7, SD = 0.02), %Boy = 50.8). # of kids in household: N = 2.3, SD = 0.007. Child race %: Hispanic = 88.5%, Not Hispanic = 11.4%. Parent relationship with child: Mother % = 78.7, Father % = 16.7, Other % = 4.5. Number of Adults in house M = 2.0, SD = 0.004. Parental education: < high school % = 4.9, high school % = 25.7. high school or more % = 69.2. NSCH 2011-2012: N = 40,446. Child Age (M = 13.5, SD = 0.02), %Boy = 51.4). # of kids in household: N = 2.3, SD = 0.01. Child race %: Hispanic = 81.5%, Not Hispanic = 18.4%. Parent relationship with child: Mother % = 71.0, Father % = 23.3, Other % = 5.5. Number of Adults in house M = 2.1, SD = 0.007. Parental education: < high school % = 21.0, high school % = 34.3. high school or more % = 44.6. | Parent Relationship with child, Mother = 78.7%, Father = 16.7%, Other = 4.5%. | Cross-sectional observational study | Data from the 2003 cycle and 2011-2012 cycles of the National Survey of Children's Health | N/A | *Self Report:* parents/guardians asked "during the past week, on how many days did [child] exercise or participate in PA for at least 20 minutes that made [him/her] sweat and breathe hard?" | Number of Days in past week child engaged in PA for at least 20 minutes : M = 4.1, SD = 0.01 | Asked several FF related questions: 1. “Is your relationship with [child] very close, somewhat close, not very close, or not close at all?” 2. “How well can you and [child] share ideas or talk about things that really matter?” 3. “In general, how well do you feel you are coping with the day to-day demands of [parenthood/raising children]?” 4. “During the past month, how often have you felt [child] is much harder to care for than most children [his/her] age?” 5. “During the past month, how often have you felt [he/she] does things that really bother you a lot?” 6. “During the past month, how often have you felt you are giving up more of your life to meet [child]’s needs that you ever expected?” 7. “During the past month, how often have you felt angry with [him/her]?”. Answers for Q1 (coded 1 to 4) (very close, somewhat close, not very close, or not close at all). Answers Q2 to Q3 (coded 1 to 4) (very well, somewhat well, not very well, not very well at all). Answers for Q4 to Q7 (coded 1 to 5) (never, sometimes, usually, and always). For the 2003 NSCH cycle that consisted of  the above-mentioned 7 items, the possible range for the family functioning index variable was 7 to 28, with higher values indicating worse family functioning. For the 2011–2012 NSCH cycle,  only 5 items were able to be assessed, with the possible range for  the family functioning index variable being 5 to 23 | *Cohesion, Communication, Conflict and General Family Functioning (i.e., family relationship)* | Multivariable Linear Regression Association Between Family Functioning (Independent Variable) and Child Physical Activity, 2003 NSCH (n=61,226) and 2011-2012 NSCH (n=40,446): 2003: is your relationship with [child] somewhat close vs. very close: –0.55 (–0.64 to –0.45) <0.001. | Multivariable Linear Regression Association Between Family Functioning (Independent Variable) and Child Physical Activity, 2003 NSCH (n=61,226) and 2011-2012 NSCH (n=40,446): 2003: how well can you and [child] share ideas or talk about things that really matter? *Somewhat well vs. very well =* –0.39 (–0.46 to –0.31), p <0.001. 2011-2012 = –0.27 (–0.38 to –0.17), p <0.001 | Multivariable Linear Regression Association Between Family Functioning (Independent Variable) and Child Physical Activity, 2003 NSCH (n=61,226) and 2011-2012 NSCH (n=40,446): 2003: During the past month, how often have you felt angry with [him/her]? *Sometimes vs. never:* 2003 = –0.10 (–0.17 to –0.01), p = 0.01. 2011-2012 = –0.15 (–0.26 to –0.05), p = 0.004 | N/A | N/A | N/A | N/A | When the family functioning index variable (ie, sum of the individual items) was used, youth engaged in less physical activity if the family had worse family functioning (for both cycles: β = –0.06, P < .001). Similarly, youth engaged in more sedentary behavior if the family had worse family functioning (for both cycles: β = 0.05, P < .001). Multivariable Linear Regression Association Between Family Functioning (Independent Variable) and Child Physical Activity, 2003 NSCH (n=61,226) and 2011-2012 NSCH (n=40,446): 2003: overall family functioning index variable = –0.06 (–0.08 to –0.05) p <0.001. 2011-2012 = –0.06 (–0.08 to –0.04) p <0.001 | Multivariable Linear Regression Association Between Family Functioning (Independent Variable) and Child Physical Activity, 2003 NSCH (n=61,226) and 2011-2012 NSCH (n=40,446): 2003 overall family FF: β = -0.06, z' = -0.06. 2011-2012: β = -0.06, z' = -0.06. Cohesion: β = –0.55, z' = -0.618. Communication: β = –0.39, z' = -0.412, β = –0.27, z' = -0.277. Conflict: β = –0.10, z' = -0.100 β = –0.15, z' = -0.151. | General FF: taken average of 2003 and 2011-2012: z' = -0.06, significant negative effect. Cohesion: taken just 2003 (2011-2012 not reported due to data availability): z'= -0.618, significant negative effect. Communication: taken average of 2003 and 2011-2012: z' = -0.345, significant negative effect. Conflict: taken average of 2003 and 2011-2012 z' values, z' =-0.126. significant negative effect. |
| Contextualizing Parental/Familial Influence on Physical Activity in Adolescents before and during COVID-19 Pandemic: A Prospective Analysis | Gilic, Ostojic, Corluka, Volaric, Sekulic (2020) | Croatia | N=688, N=322 females. 17 years old at the baseline period of study, and between 15-18 years. 445 participants (65%) (N=202 females) resided in urban centers, 35% in rural communities. SES of family: below average boys = F=12(3.3%), average boys = F=328(89.6%), above average boys = F= 26(7.1%). below average girls =F=2(0.2%), average girls = F= 308(95.7%), above average girls =F=12(3.7%). Paternal education: elementary school boys = F= 16(4.4%), high school boys F = 257 (70.2%), college boys =F = 47 (12.8%), university level boys=F= 46(12.6%). elementary school girls = F= 36(11.2%), high school girls F = 226 (70.2%), college girls =F = 36 (11.2%), university level girls =F= 24(7.5%). Maternal education: elementary school boys = F= 84(23%), high school boys F = 238 (65%), college boys =F = 24 (6.6%), university level boys=F= 20(5.5%). elementary school girls = F= 120(37.3%), high school girls F = 160(49.7%), college girls =F = 20(6.2%), university level girls =F= 22(6.8%). | Not disclosed. Most likely father, mother, child dyads, based on extracted data. | longitudinal - however I think originally meant to be cross-sectional, however COVID-19 measures in place which impeded data collection. | Baseline testing (PA levels, parental/familial factors, anthropometrics) occurred Jan 6-12. COVID-19 restrictive measures prevented further data collection until April 20-26 where follow-up testing for PA levels was done. Variables included in the study were Sociodemographic factors such as age, gender, familiac/parental factors (predictors), and PALs criteria. Normality of distribution checked w/ Kolmogorov-Smirnov test. Mean and SD for PAQ-A, and age were calculated. To identify associations between predictors (sociodemographica, parental/familial variables), and dichotomized PAL-criteria, logistic regressions were calculated, with Odds Ratios and corresponding 95% CI values reported. Since girls were slightly older than boys, and preliminary statistics identified significantly influence of age and gender on PAL - logistic regressions were calculated as crude model (model 0), and additionally controlled for gender and age as covariates (model 1). p-value of 0.05 was applied. | This study is part of another large study (physical activity, substance misuse, and factors of influence in adolescence) | *Self-report:* PAQ-A: physical activity questionnaire for adolescents was used to assess PALs at baseline and at a follow-up measurement period. 7 day recall and self-administered questionnaire that was developed to measure the PAL of adolescents aged 14 to 19. Items are scored on a scale from 1 to 5 with 1 representing no activity, or a low activity level, and 5 representing a high PA level. | Baseline - all participants attended regular physical education classes 2x per week, and some adolescents also took part in extracurricular sport activities. PAQ-A score total: baseline to follow up = 2.98 ± 0.71 to 2.31 ± 0.68; t-test: 11.88, p < 0.001. Girls from baseline to follow up = 3.12 ± 0.56 to 2.50 ± 0.44; t-test: 10.01, p < 0.01. Boys from baseline to follow up = t-test: 12.55, p < 0.001, t-test: 11.99, p < 0.001 | (i) “How often do you have a conflict with your parents/family?” (never–rarely–from time to time–regularly/frequently); (iv) “How would you rate how much your parents/family care about you and your personal life?”  (Very poor care–Low care–My parents/family care about me–My parents/family care about me a lot). | *Cohesion and Conflict* | A positive correlation between family cohesion and children's PAL has been recorded. Estimates based on graph: Model 0 = (OR: 0.83, 95% CI: 0.60-1.0.5) Model 1 = (OR: 0.86, 95% CI: 0.62-0.1) | N/A | Family conflict was found to have a negative association with physical activity levels among adolescents in both testing waves (before and during COVID-19 pandemic). A lower likelihood of a sufficient level of PAL at baseline was found for adolescents who reported a higher level of conflict with parents/family members (OR: 0.72, 95% CI: 0.57-0.90). Significant correlations were found between familial conflict and follow-up PAL (OR: 0.77, 95% CI: 0.60-0.99) with a lower likelihood of sufficient PAL at the time of follow up among adolescents who reported a higher level of conflict with their parents/family. | N/A | N/A | N/A | N/A | N/A | Conflict: OR = .72, z' = -0.090. OR = 0.77, z' = -0.072. Cohesion: OR = 0.83, z' = -0.051. OR = 0.86, z' = -0.042 | Cohesion: used model 0 (seemed closest to bivariate): z' = -0.051, significant negative association. Conflict: used model 0 (closest to bivariate): z' = -0.090, significant negative association. |
| Paternal Closeness in Adolescence: The Association of Sports and Gender | Leppard, Dufur (2022) | USA | N = 5700. Mean/proportion = Male: 48.63 Female: 51.37. Self reported racial group = White: 60.35 Hispanic: 10.65, Black: 21.21, Native: 2.61, Asian: 4.04, Other: 1.14. Parental income = 42.337, SD = 26.355. parental education = 14.12, SD - 3.311 | Mean/proportion = Two parent bio: 50.47 Blended: 15.67 Single parent: 33.86. Number of siblings = 1.385 | Cross sectional | Data from Wave 1 of the National Longitudinal Study of Adolescent to Adult Health (Add Health) was used. Background characteristics like race, SES, parental health, family structure, and number of siblings was applied as control variables. Used an ordered logistic regression analysis to predict adolescents' perceptions of closeness to father. | Data from Wave 1 of the National Longitudinal Study of Adolescent to Adult Health (Add Health) was used. | *Self-report:* participation in sport using a binary variable indicating whether respondents had participated in any sport in the last year (1 = participated, 0 = did not participate) | Mean/proportion: No sport boys: 17.48 Sport boys: 31.11 No sport girls: 25.9 Sport girls: 25.46 | *Us*ed questions measuring closeness/relationship with fathers that are embedded in the AddHealth data. Perceived closeness between adolescents and fathers was indicated by a variable reported by adolescents asking how close they feel to their father. Responses ranged from 1 (not at all close to my father) to 5 (very close to my father). | *General Family Functioning (i.e., family relationship)* | N/A | N/A | N/A | N/A | N/A | N/A | N/A | Model 1 for each outcome includes only positive relationship between sport participation and relationships with fathers (p<0.001 for both outcomes). Ordered Logistic and OLS Regressions of Perceived Relationship with Father and Activities with Father by Participation in any Sport: Model 1: (any sport and perceived relationship) = 1.327, p<0.001 (0.074), Model 3 (any sport and perceived relationship) = 1.189(0.069). Ordered Logistic Regression of Perceived Relationship with Father by Sports Participation for Boys and Girls: Boys sport: mean perceived relationship = 4.28. Girls sports: mean perceived relationship = 3.96. | Ordered Logistic and OLS Regressions of Perceived Relationship with Father and Activities with Father by Participation in any Sport: Model 1: (any sport and perceived relationship) OR = 1.327, z' = 0.078. Model 3 (any sport and perceived relationship) OR = 1.189, z' = 0.048. | General FF: Used model one for calculations (closest to bivariate): 0.078. Positive, significant. |
| Effects of parent-adolescent reported family functioning discrepancy on physical activity and diet among Hispanic youth | Lebron, Lee, George, Messiah, Park, Prado (2018) | USA | N = 280 dyads. Adolescent: (Female = 52%. Age: M = 13.01, SD = 0.83. Country of Origin: USA = 64%, Cuba = 19.3%, Honduras = 4.3%, Venezuela = 3.6%). Parent: (Female = 88.2%. Age: M = 44.88, SD = 6.5. Country of Origin: USA = 8.9%, Cuba = 34.3%, Nicaragua = 15%, Honduras = 11.4%. Annual income: less than $30,000 = 65.4%, greater than $30,000 = 20.7%, greater than $50,000 = 13.9%. Marital status: married = 57.9%, divorced = 12.9%, living w/ someone = 10%, separated = 10%, never married & not living w/ someone = 8.6%, widowed = 0.7%. Employment: full time employment = 50.7%.) | Hispanic overweight and obese 7th and 8th grade youth and their primary caregivers. | Secondary data analysis study | Data utilized from the baseline assessment of a randomized controlled trial conducted in collaboration with the Miami Dade County Public School System. The study investigated differences between parent and adolescent-reported family functioning scores using t-tests and correlations. It standardized these differences and assessed their relationship with obesity-related behaviors using SEM, controlling for gender and BMI. Analysis was conducted using Mplus software with FIML estimation to handle missing data. | Data was obtained from the baseline assessment of a randomized controlled trial conducted in collaboration with the Miami Dade County Public School System. | *Self-report:* MVPA assessed by asking youth: "During the past 7 days, on how many days were you physically active for a total of at least 60 minutes per day?" Add up all the time you spend in any kind of physical activity that increase your heart rate and make you breathe hard some of the time" - based on the NHANEs physical activity and physical fitness questionnaire. |  | FF assessed using adolescent and parent reports of 5 indicators: positive parenting, parental involvement, family communication, parental monitoring of peers, and parent-adolescent communication. Corresponding subscales from the parenting practices scale were used to assess positive parenting and parental involvement. The communication subscale from the Family Relations Scale was used to assess family communication, sample questions included "My family and I have the same views about what is right and wrong" and "My family knows what I mean when I say something"; response choices ranged from 1 = not true at all to 3 = true a lot. The Parent-Adolescent Communication Scale was used to assess parent-adolescent communication (20 items): adolescent: "When I ask questions, I get honest answers from my mother/father" and for parent " parent: "when I ask questions I get honest answers from my child". response choices ranged from 1 = strongly disagree to 5 = strongly agree. | *Communication and General Family Functioning (i.e., family relationship)* | N/A | Descriptive Statistics Among Study Variables: P-A communication (parental report) and physically active days = .07, p>0.05. P-A communication (adolescent report) and physically active days = 0.06, p>0.05 | N/A | N/A | N/A | N/A | N/A | General Family Functioning Discrepancy: *the effect of discrepancy in parent-adolescent family functioning ratings on obesity-related behaviors.* FF and physically active days (At least 60 mins per day) β = -.14*, 95% CI (-.26, -.05), p<0.05. | General FF: β = -0.140, z' = -0.141. Communication: r = 0.07, z' = 0.07. r = 0.06, z' = 0.060. | General FF: significant, negative, -0.141. Communication: Used parental report (based off of standards decided for this study) z' = 0.07, not significant positive effect. |
| Child and family factors associated with child mental health and well-being during COVID-19 | McArthur, Racine, McDonald, Tough, Medigan (2021) | Canada | n=846. Child sex, no.(%) = Male 447 (52.8), Female 398 (47.1), Missing 1 (0.1). Child age, no. (%) = 9 years 334 (39.5) 10 years 308 (36.4) 11 years 203 (24.0) Missing 1 (0.1) Maternal race/ethnicity, no. (%)= Asian 104 (12.2) Black 4 (0.5) First Nations, Inuit, Metis 2 (0.2) Latin 11 (1.3) Other/mixed 22 (2.6) White 701 (82.9) Missing 2 (0.3) Household income before COVID-19, no. (%) = ≥$80,000 679 (80.3), ≤$79,999 139 (16.4), Missing 28 (3.3) | not disclosed. | cross-sectional | Data was drawn from the all our families cohort, specifically between May 2020 to August 2020. In order to control for pre-pandemic factors, a maternal report of children's pre-pandemic anxiety, depressive symptoms, or adaptive skills at 8 years of age (2017-2019) was used. Child sex was also controlled for. mother's self reported symptoms of depression from May -July 2020. Children reported parent-child relationship, time spent on electronic device, PA, sleep, recreational activities and connectedness to school peers (July-August 2020).Multivariable linear regression analysis was used to predict each outcome independently—anxiety, depression, and happiness. Effect modification was tested in each of the regression models based on child sex (male, female), child age (9, 10, 11), and previous at-risk mental health status (T-score≥60 vs. T-score<60) at 8 years of age (BASC-2), using Chi-square difference tests with the Satorra–Bentler scaling correction to  adjust for nested models. | All Our Families cohort in Calgary, Canada | *Self-report:*  how many of the last 7 days they participated in PA that made their heart rate go up for at least 60 minutes (0, 0 days; 7, 7 days). | not disclosed. | children reported on their parent-child relationship using the connections with adults at home subscale of the middle years development instrument. Children indicated on three items (e.g., “In my home there is a parent or another adult who listens to me when I have something to say.”) how much they agree with each statement (1, not at all true; 4, very much true) and higher scores indicated greater connection to caregivers. | *Cohesion* | Correlations among COVID-19 predictors, pre-pandemic mental health, and COVID-19 child mental health and well-being: connection to caregivers and physical activity = 0.06, p > .05 | N/A | N/A | N/A | N/A | N/A | N/A | N/A | Cohesion: Pearson r = 0.06, z' = 0.060 | Cohesion: not significant, positive, 0.060. |
| Physical activity intensity among adolescents and association with parent–adolescent relationship and well-being | Shennar-Golan, Walter (2018) | Israel | 233 adolescents, in grades 8 to 12, 126 (54.1%) girls and 107 (45.9%) boys, aged 13 to 18 years (girls: M = 15.26, SD = 1.43; boys: M = 15.44, SD = 1.31) | Not disclosed. | Cross sectional | Research questionnaires were distributed; students completed them anonymously in class. The data were cleaned, coded, and analyzed using SPSS version 18. Descriptive statistics were used to describe the sample and the main variables. Measures included a demographic questionnaire, godin shephard leisure time PA questionnaire, welling being, parent-adolescent relationship and BMI. Data were analyzed—using Pearson correlations, t tests,  and regressions—to assess the effects of BMI, SWB, and parent–adolescent relationship on intensity of PA across sexes. A multiple regression was run to determine the contributions of these independent variables to explaining the variance in the intensity of PA participation. All predictors were standardized.  Pearson correlations were computed to analyze the relationships between intensity of PA, the dependent variable; the independent variables SWB, sex, and parent–adolescent relationship; and BMI as an objective measure. Parent– adolescent relationship was analyzed in relation to BMI, SWB, and PA intensity. A t test assessed the mean differences in the variables by sex. A partial Pearson correlation assessed the connection between PA level, SWB, parent– adolescent relationship, and BMI. Finally, a multiple regression was run to determine the contribution of the independent variables to explaining the variance in PA intensity. | N/A | *Self-report:* Godin-Shephard Leisure-Time Physical Activity Questionnaire - a four-time self-administered questionnaire. Gathers information on the number of times one engages in light (minimal effort), moderate (not exhausting), and strenuous (heart beats rapidly) leisure-time PA (LTPA) of at least 15-min duration during a typical 7-day period. Then, each frequency score is multiplied by a corresponding metabolic equivalent of task value (i.e., 3, 5, and 9 for light, moderate, and strenuous intensity, respectively) and summed to obtain a leisure score index expressed in arbitrary units. | moderate physical activity M = 2.2 (SD = 2.1), strenuous physical activity M = 2.7 (SD= 2.3) | parent-adolescent relationship was measuring using the parental attachment scale. Parental attachment in this study was measured by six items assessing the affective component of the parent-child bond in terms of affection, caring and mutual respect. The sum of the items is totaled for an overall score ranging from 0 to 24. Higher mean total scores indicate greater attachment to parents. | *General Family Functioning (i.e., family relationship)* | N/A | N/A | N/A | N/A | N/A | N/A | N/A | A nonsignificant correlation was found between parent-adolescent relationship and moderate PA, r(233) = .013, p > .05; a significant weak correlation was found for strenuous PA, r(223) = .150, p<0.01. Means, Standard Deviations, and Intercorrelations of Study Variables (N=233): parent-adolescent relationship and light activity r = -0.046, p >.05; moderate activity = r = 0.13, p > .05; strenuous activity r = .150, p < .05. Means, Standard Deviations, and Intercorrelations for Study Variables, by Sex: boys: parent-adolescent relationship and light activity = -0.001, p > .05; moderate activity = 0.062, p > .05, strenuous activity = .220, p < .05; girls: parent-adolescent relationship and light activity r = -0.082, p > .05; moderate activity r = -0.029, p > .05; strenuous activity r = .185, p < .05 | Pearson r = .150, z' = 0.151. Means, Standard Deviations, and Intercorrelations of Study Variables (N=233): light activity: r = -0.046, z' = -0.046. moderate activity: r = 0.13, z' = 0.131. strenuous activity: r = .15, z' = 0.151. Means, Standard Deviations, and Intercorrelations for Study Variables, by Sex: BOYS: light activity r = -0.001, z' = -0.001. moderate activity r = 0.062, z' = 0.062. strenuous activity r = .220, z' = 0.224. GIRLS: light activity r = -0.082, z' = -0.082. moderate activity r = -0.029, z' = -0.029. strenuous activity r = .220, z' = 0.187. | General FF: taken average of strenuous and moderate to get closest to MVPA (standard for study): z' = 0.141, significant, positive (between moderate and strenuous p-values). |
| Healthy youth development: The role of youth assets | MacKay (2007) - dissertation | Canada | n = 30588. 49.7% = male, 50.3% = female. Ethnicity = 61% European background, 18% east Asian background, 7% aboriginal background, 5% southeast Asian background, 4% southeast Asian background, 2% African, 2% west Asian, 3% other, 8% unknown. | Not disclosed/measured. | Cross sectional with a stratified cluster sampling design | Data was derived from the BC AHS III - surveyed 30,588 BC students from grades 7 to 12 in the spring of 2003. BC AHS III = 140 item test assessing factors related to adolescents physical and emotional health. Demographic variables, family connectedness, parental presence at home, school connectedness, other adult support, positive peer influence, religiosity, perceived competence, volunteerism, extracurricular activities, perceived health status, PA, academic achievement, educational expectations, seatbelt and bicycle helmet use were measured. | British Columbia Adolescent Health Survey (BC AHS III) | *Self-report:* PA was assessed by asking students on how many of the past 7 days did they exercise for at least 20 minutes to the extent it made them sweat or breathe hard. Extracurricular activities questionnaire: 6 questions: how often they played sports without a coach, played sports with a coach, take part in a dance or aerobic class (more Qs, but unrelated to PA) | 66% played sports with a coach, 71% played sports w/out a coach in the past year, 31% took a dance or aerobic class. | Family connectedness: A student's feeling of connectedness to his or her family was measured by 11 questions. the items were on a scale ranging from 1 to 3. | *Cohesion* | Correlations Among Youth Assets and Health Outcomes: Family connectedness and physical activity r = .128, p < .0001 Statistics for Best Subset for Physical Activity: Family connectedness B = .56, SE = 0.01, β = .10, t = 45.89, tolerance = .75, contribution to R^2 = .01, p < .001 | N/A | N/A | N/A | N/A | N/A | N/A | N/A | Cohesion r = .128, z' = 0.129 | Cohesion: significant, positive, 0.129. |
| Health outcomes in adolescence: associations with family, friends and school engagement | Carter, McGee, Taylor, Williams (2007) | New Zealand | N = 643. 326 = males, females = 317. Median age = 15 years and 10 months. 91.3% = NZ European, 9.5% = Maori, 2% = Cook Island, 9% = Other. | not disclosed/measured | Cross-sectional prevalence survey | 12 Dunedin (NZ) high schools were invited to take part in the survey and were asked to select every second Year 11 student. Based study around the American youth risk behaviour survey - modified slightly to suit language and specific issues related to NZ youth. Study assessed family and friends connectedness, and school climate. Study data collected occurred between july and October 2001. Survey took most students around less than 30 min to complete. The study examined the association between family, friends, and school factors and both health-compromising and health-promoting behaviors in adolescents. Health-compromising behaviors included substance use, depressed mood, physical aggression, and sexual activity, while health-promoting behaviors include physical activity, fruit and vegetable consumption, safe sexual activity, and sun protection. Responses were coded as 1 for positive behaviors and 0 for negative behaviors. The study used generalized estimating equations (GEE) to analyze the data, considering the multilevel structure of the schools as the primary sampling units. | N/A | *Self-report:* "how often did you engage in PA for at least 20 min a day that made you sweat and breathe hard on at least 3 of the past 7 days" | Vigorous PA on at least 3 days in the last 7 days: male = 65.7%, female = 51.1% | Measure connectedness to family and friends: "who do you talk to when you have a problem or feel upset about something?’’ and ‘‘who takes notice of you  (e.g. understands, comforts, asks what is wrong) when you are upset or angry about something?’’  Response options included a list of family members, friends, other individuals (e.g. religious  minister), and ‘‘no-one.' | *Cohesion* | Multivariate models for school, family, and friends and health promoting behaviours: medium family connectedness and vigorous physical activity on at least 3 days in last 7 days OR = 1.58 (95% CI: .94-2.67), p > .05; high family connectedness OR = 1.96 (95% CI: 1.09-3.55), p < .05 | N/A | N/A | N/A | N/A | N/A | N/A | N/A | Cohesion: MVPA and medium cohesion: OR = 1.58, z' = -0.489. MVPA and high cohesion: OR = 1.96, z' = 0.184 | Cohesion: taken average of medium and high cohesion z' values: z' = -0.153. significant positive. |
| Factors associated with children's health in Taiwan and the United States | Chen (2002) | Taiwan/USA | 95 children and their mothers in Taiwan, 68 children and their mothers in the Bay area. Taiwan = 42 boys, 53 girls, 31 8 year old children, 33 nine year old children, 31 ten year old children. average age of mothers = 35.94 (SD=3.91), average amount of education = 11.1 years (SD = 2.77). 89 mothers = married, 4 = divorced, 1 = single, 1 = widowed. USA = 31 boys, 37 girls. 8 year olds = 23, 9 year olds = 23, 10 year olds 22. Average age of mothers = 41.09 (SD = 3.81), average amount of education = 13.43 years (SD = 4.16). 65 mothers = married, 2 = widowed, 1 = single. In both sites, the majority of the parents were married (ROC-93.7%; CA-95.6%). In ROC, 4.7% of the family had annual income less than NT$12,000 (low income) whereas 6.0% of the CA family had annual income less than US$10,000, 34.8% of the family in ROC had annual income greater than NT$85,000 (high income) while 44.8% of the family in CA had annual income greater than US$40,000.The total of 163 children included slightly more girls (55.4%) than boys (44.6%) in the study. In total sample, approximately one third of children were eight years old (N=54), one third were nine years old (N=54) and one third was ten years old (N=53). Both sites had similar proportions of age group children. | mother-child dyads answering the questionnaire. | Cross-sectional study design | Taiwan, participants were recruited through Nan-Chang elementary school. USA: recruited through two Chinese language schools located in the bay area of San Francisco. Mothers filled out questionnaires at home. Standard procedure for measuring body mass was used. 24 hours dietary intake and PA between the same day interview and next day interview. Taiwan = children in the same grade were asked on Monday, Tuesday and Friday morning to record what they did (type and intensity of activity) and ate during the previous day (Sunday, Monday and Thursday). USA = chldren recorded their dietary intake and activity questionnaires on Sunday, Monday and Thursday at home.  The statistical methods utilized in the study served to determine associations between factors and children's body composition. Cronbach's alpha and KR-20 assessed the reliability of measurement scales, while chi-square tests and t-tests compared scores across demographic variables. Pearson correlation coefficients examined relationships between variables and BMI, while stepwise multiple linear regressions identified significant predictors of body composition, considering variables from the correlation matrix and controlling for confounding factors like country. | N/A | *Self-report:* children self administrated physical activity checklist: Self-reported - Taiwan, child taught to estimate PA (e.g., recess lasts for 10 min, PE class for 45 min). Children were asked to recall activities that occurred the previous day before school, during school, and after school. In the original questionnaire, there are 25 activities and two questions related to TV/video viewing and video/computer game time |  | Family Assessment Device - 60 item self report questionnaire measuring 6 dimensions of FF based on the McMaster model of FF; 6 subscales (problem solving, communication, roles, affective responsiveness, affective involvement, and behavior control) . Plus a 12-item general functioning scale used as a global assessment of general health of the family. 4-pt Likert type scale ranging from strongly agree, agree, disagree, and strongly disagree, is utilized to evaluate a family member's perception of the family. Score from 1 to 4; 4 = unhealthy functioning, 1 = healthy functioning. | *Communication, Organization, Problem Solving Ability, Affective Environment, and General Family Functioning (i.e., family relationship)* | N/A | Pearson Correlation Coefficient = Communication and MVPA METs = .001 (p = .993); Communication and MVPA minutes = 0.001 (p = .993). no relationship was found between family communication and children's PA level. | N/A | Pearson Correlation Coefficient = FAD-Roles and MVPA minutes = -0.102 (p = .194). | There was no correlation between affective responsiveness and MVPAC MET (r = -.087, p = .267), MVPAC minutes (r = -.090, p = .251). no relationship between family affective interaction and children's PA level. | Pearson Correlation Coefficient: FAD-problem solving and MVPA minutes r = -.119 (p = .129); FAD-problem solving and MVPA METs r = -.072 (p = .358) |  | Pearson Correlation Coefficient = FAD general functioning and MVPA minutes = -.123(.119). | Communication and MVPA minutes r = 0.001, z' = 0.001. Organization and MVPA minutes r = -.102, z' = -.102. Problem Solving Ability and MVPA minutes r = -.119, z' = -0.120. Affective Environment: r = -0.09, z' = -0.090 General FF and MVPA r = -.123, z' = -0.1240 | Communication: positive, not significant, 0.001. Organization: negative, not significant, 0.102. Problem-solving ability: negative, not significant, -0.120. Affective environment: not significant, negative, -0.090. General FF: not significant negative, -0.1240. |
| Parental influences on adolescent physical activity: a longitudinal study | Ornelas, Perreira, Ayala (2007) | USA | Females: White 3,654 60.8%, males: 3,541 72.7%. Females: African-American 1,485 49.4%, males: 1,307 74.4%. Females: Hispanic/Latino 1,105 52.8%, males: 1,141 70.9%. Females: Asian-American 486 52.5%, males: 527 70.3%. Females: Immigrant Generation Third or More 5,246 58.2%, males: 5,027 72.5%. Females: Second 965 58.9%, males: 720 75.1% . Females: First 519 47.2%, males: 493 68.7% . Females: Parent Education College graduate or more 2,280 61.6%, males: 2,389 77.2%. Females: Some college 1,354 60.0%, males: 1,216 70.9%, females: High school graduate 1,896 55.4%, males: 1,834 71.5%. Females: Less than high school graduate 870 53.7%, males: 735 67.4% | Family Structure Two-parent family females: 3,573 58.9%, males: 3,516 73.5%. Females: Step-parent family 1,124 60.3%, males: 1,177 71.1%. Females: Single mother 1,538 55.3%, males: 1,290 73.2%. Female: Single father 152 59.7%, males: 238 70.2%. females: Other 343 42.9%, males: 295 66.4% | Longitudinal | Wave I in-home interviews were conducted from April to December 1995 with a random sample of 20,745 students. The study utilized data from the National Longitudinal Study of Adolescent Health (Add Health), a nationally representative study of U.S. adolescents in grades 6 through 12. Data were collected through in-home interviews, with measures including physical activity, family cohesion, parenting, self-esteem, and depression. Physical activity was assessed using a standard seven-day recall scale, while family cohesion, parenting, and other variables were measured through questionnaires. Analyses involved descriptive statistics and logit models for physical activity, examining family influences on adolescents' well-being. Covariates included age, race/ethnicity, immigrant status, family structure, and parental education. The study used STATA for statistical analysis, employing survey estimation procedures to correct for sample design and weights. Descriptive statistics were calculated for all variables, including frequencies, means, and standard errors. Differences in proportions of engaging in vigorous physical activity (MVPA) were tested across various demographic factors. Logit models were used to identify the likelihood of engaging in MVPA, with odds ratios reported. Models were estimated individually for each parental influence variable and then together. Mediation by self-esteem and depression was tested. Analyses were stratified by gender and adjusted for demographic factors. Missing values in parenting variables were addressed using mean substitution, with results showing minimal influence. | Add health | *Self-report:* measured at wave II. Measured total weekly bouts of MVPA. This outcome, examined in several previous studies was derived using a standard seven day physical activity recall scale similar to those used in other large-scale studies although not capturing time period. Questions were worded, "During the past week, how many times did you...," followed by a list of activities, allowing calculation of the number of physical activity bouts per week. MVPA activities included skating, cycling, exercise and active sports, and had an estimated energy cost of five to eight METs (metabolic equivalent values; 1 MET = resting metabolic rate, or 3.5 ml 02 body weight/minute). Overall physical activity frequency was summed to obtain total weekly bouts of MVPA. Then, an indicator variable (1/0) was created based on whether the adolescent met the 1995 national recommendations for physical activity | Physical Activity Bouts of physical activity per week: females: M = 6.03 (s.e. = 0.11). Males = 7.88 (s.e. = 0.11) | family cohesion: measured summing responses to adolescent reports for three items (ranging from 1 = low to 5 = high) on how much people in their family understand them, how much they and their family have fun together, and how much their family pays attention to them. Parent-child communication was calculated as the sum of three types of communication that adolescents had with his/her primary caregiver in the last 4 weeks (talking with them about dating, a personal problem, and a school work; range 0 = low to 3 = high). | *Cohesion and Communication* | Partially Adjusted Logits on Moderate to Vigorous Physical Activity by Gender: Females: family cohesion = OR = 1.09, 95% CI = 1.05-1.12, p </.001; Males OR = 1.08, 95%CI = 1.04-1.11, p </ .001 Fully Adjusted Logits on Moderate to Vigorous Physical Activity, by Gender: Family cohesion (female) OR = 1.05, 95%CI = 0.00-1.09, p </ .01; male family cohesion = OR = 1.05, 95%CI = 1.02-1.09, p </ .01 | Partially Adjusted Logits on Moderate to Vigorous Physical Activity by Gender: Females: parent-child communication = OR = 1.13, 95% CI = 1.07-1.19, p </ .001; Males OR = 1.14, 95%CI = 1.07-1.23, p </ .001. Fully Adjusted Logits on Moderate to Vigorous Physical Activity by Gender: Female: Model 1: parent-child communication = OR = 1.06 (95% CI: 1.00-1.12). p<0.05. Male: Model 4: parent-child communication = OR = 1.08(95%CI: 1.01-1.16), p<0.05. | N/A | N/A | N/A | N/A | N/A | N/A | Partially Adjusted: Female cohesion OR = 1.09, z' = 0.024. Male Cohesion OR = 1.08, z' = 0.021. Fully Adjusted: Female cohesion OR = 1.05, z' = 0.013. Male cohesion OR = 1.05, z' = 0.013. Partially Adjusted: Female Communication OR = 1.13, z' = 0.034. Male Communication OR = 1.14, z' = 0.036. Fully Adjusted Female: Model 1 = OR = 1.06, z' = 0.016. Fully Adjusted Male: Model 4 = OR = 1.08, z' = 0.021. | Cohesion: taken average of partially adjusted, male and female (less variables): z' = 0.0225, significant, positive. Communication: taken average of partially adjusted z' values for males and females: z' = 0.035, significant, positive. |
| Social Anxiety May Modify the Relationship Between Internet Addiction and Its Determining Factors in Chinese Adolescents | Dong, Zhao, Wu, Wang, Li, Zhang, Sun (2018) | China | n = 10,158. Boys: 4716, Girls: 5442. Age: <18 = 1834, >18 = 8324. Residence: Rural: 7729,  Urban: 2429. Only child: Yes: 2487, No: 7671. Living conditions: Both parents: 8632, Others: 1526. Mother’s educational level: Primary school or below: 4844, Above primary school: 5221. Father’s educational level: Primary school or below: 2315, Above primary school: 7766 | not disclosed - assuming father, mother - child dyads. | Cross-sectional | Cross-sectional study in Anhui's occupational schools (vocational high schools and higher vocational colleges) with 10,574 adolescents, investigating internet addiction risk due to unique characteristics. Random cluster sampling, 96.07% participation. Sociodemographic variables, Internet Addiction (IA), Social Anxiety Symptoms, Self-esteem assessed. Chi-square, t-test, logistic regression, correlation, path analysis used. Factors associated with IA analyzed to determine direct or indirect association. | N/A | *Self-report:* Physical exercise (days/week) was divided into three grades: (1) 0 days, (2) 1~3 days, and (3) >3 days. | 0 = 2042, 1~3 = 6520, >3 = 1596. 64.2% reported physical exercise was 1~3 days/week. | parent-adolescent relationship classified as good or bad. | *General Family Functioning (i.e., family relationship)* | N/A | N/A | N/A | N/A | N/A | N/A | N/A | father-adolescent relationship was positively correlated with physical exercise. Father-adolescent relationship and physical exercise only had an indirect effect on internet addiction, mediated by self-esteem and social anxiety. The correlation coefficient between variables: Father-adolescent relationship and physical exercise = 0.040, P<0.01. | r = 0.040, z' = 0.040. | General FF: positive, significant, 0.040. |
| Association of lifestyle behaviours with self-esteem through health-related quality of life in Spanish adolescents | Knox, Muros (2017) | UK | N = 456. Age (years) 12.57 ± 1.17/. Gender (% male) 51.5%  BMI (kg/m2) 19.75 ± 3.85. | not disclosed/measured. | Cross-sectional | Study sampled 456 adolescents aged 11-14 from five randomly selected schools in 2014. Sample recruited from medium-high socioeconomic areas. Written informed consent obtained from parents/guardians. Ethical approval from University of Granada. No incentives provided. Data collection during physical education lessons. The study utilized path analysis to explore how components of HRQoL mediate the relationship between physical activity/diet adherence and self-esteem, while adjusting for BMI and gender. Additionally, bootstrapping was employed to estimate indirect effects in the mediation models. This involved analyzing four paths: a (from the independent variable to the mediator), b (from the mediator to self-esteem), c (from the mediator to self-esteem), and c' (the direct effect of the independent variable to self-esteem). The significance of each path was determined by examining bootstrapped confidence intervals, with 1000 bootstrap resamples used to ensure statistical robustness. | N/A | *Self-report:* PA levels evaluated using the PA questionnaire for older children (PAQ-C). The questionnaire provides a general measure of physical activity for 8- to 20-year-olds. The PAQ-C is a self-administrated questionnaire consisting of nine items rated on a five-point scale. A higher score indicates more active children. | PA score: 2.92 ± 0.64 | Family relationships and autonomy support score - part of KIDSCREEN-27 questionnaire assessing health-related quality of life. Specifically assesses relationship with parents | *General Family Functioning (i.e., family relationship)* | N/A | N/A | N/A | N/A | N/A | N/A | N/A | Physical activity was related with family relationships, and autonomy support, and social relationships with peer pressure: (path a; β = 3.95, SE = 0.77, p < 0.001; β = 3.27, SE = 0.75, p < 0.001). Mediation Analysis of the influence of physical activity engagement on self-esteem through the five components of wellbeing: PA and autonomy and family = 2.74 | N/A: used non standardized beta values - cannot transform to z' | General FF: significant, positive - based off of p-values. |
| Predictors of Leisure Participation in 6 to 14-Year-Old Children with Cerebral Palsy: Structural Equation Modeling | Ghaffari, Kalantari, Rezaee, Akbarzadeh Baghban (2019) | Iran | n = 232. Sex: Boys: 141 (60.8%), girls: 91 (39.2%). Father education: Lower than Diploma: 178 (76.7%), Bachelor: 43(18.5%), Master and higher: 11(4.7%). Mother education: Lower than Diploma: 193 (83.2%), Bachelor: 36(15.5%), Master and higher: 3(1.3%). Father job: Government job: 58 (25%), Private job: 20 (8.6%), Self-employed: 118 (50.9%), unemployment: 36 (15.5%). Mother job: Government job: 17 (7.3%), Private job: 8 (3.4%), Self-employed: 17 (7.3%), housekeeper: 190 (81.9%) | Number of children: 1: 77(33.2%), 2: 106 (45.7%), >3: 49 (21.1%) | Cross-sectional | study enrolled 232 cerebral palsy children aged 6 to 14 and their parents from schools and rehabilitation centers in Shiraz, Iran. Inclusion criteria included age 6 to 14, cerebral palsy diagnosis, parental ability to read and write, and no history of other neurological diseases. The Persian versions of the Children’s Assessment of Participation and Enjoyment (CAPE), Family Environment Scale, Craig Hospital Inventory of Environmental Factors, and Strengths and Difficulties Questionnaire were used for assessment. Data collection involved self-administered questionnaires completed by parents and children, with assistance if needed, and demographic information was gathered. The structural model, tested using AMOS software, comprised measurement and structural analyses. Some paths, including age, caregiver education, and income, were excluded due to fit. | N/A | *Self-report:* Persian version of Children's Assessment of Participation and Enjoyment (CAPE) for evaluating the rate of leisure participation in children. 55-item measure of leisure and recreational activities done during the past four months. The dimension “intensity” (“how often” activities were done) is rated on a seven-point scale from 1, “once in the past four months” to 7, “once a day or more” | Children with CP mostly participate in recreational (mean: 34.4, SD:14.1) activities. | The family environment scale has 90 (true and false) two way questions. This scale assesses the social climate of the family, the interpersonal relationships of family members and the family's efforts to maintain iteself in critical situations. The questionnaire has 10 subscales, and we measured the subscales of cohesion, organization, and conflict for family structure and relationship construct and the subscales of intellectual cultural orientation and family participation in recreation for family activity orientation construct. | *General Family Functioning (i.e., family relationship)* | N/A | N/A | N/A | N/A | N/A | N/A | N/A | Correlations Among Study Variables: family structure and relationship and intensity of child participation = .172, p<0.01. | r = 0.172, z' = 0.174 | General FF: positive, significant, 0.174. |
| Parental bonding may moderate the relationship between parent physical activity and youth physical activity after school | Dzewaltowski, Ryan, Rosenkranz (2008) | USA | n=57. 23-36% were eligible for free and reduced lunch status (lower SES status). 25 = sixth grade, 32 = seventh grade students. Age = 11 to 13 years, M = 12.4 years. n =37 girls, n = 18 boys. 46 = Caucasian, 4 = Asian-Americans, 2 = African Americans, 2 = African Americans, 2 = Hispanics, 1 = Native American. | not disclosed/measured. | Cross-sectional | A convenience sample of 57 students from elementary and middle school classrooms. Students completed physical activity and bonding surveys, along with multiple Previous-Day Physical Activity Recall assessments administered by registered dieticians. Descriptive stats for parental variables and youth after-school physical activity. ANOVA tested grade and gender differences. Moderation hypothesis tested via hierarchical multiple regression, centralizing first-order effects, then entering their product. Significant R² change indicated a moderator effect (α=0.05). | N/A | *Self-report:* PA measurements were focused on after-school time. The PDPAR was used to measure the level of youth's PA after school. Validated measure requires youths to report the type and intensity of activity they engaged in during each half-hour block of time (3:00pm to 11:30pm) the previous day. The, each half hour block is assigned a MET value, based on the type and intensity of activity. Activities are coded as MVPA if the assigned MET value is three or greater, and as vigorous physical activity (VPA) if the assigned MET value is six or greater. The recall was completed three times, and data from a minimum of two recalls were used to calculate the average number of half-hour blocks of MVPA and VPA. | Youth engaged in 3.467 +/- 2.1 half-hour blocks of activity >3 METs (MVPA) and 1.637 +/- 1.8 half-hour blocks of activity >6 METS (VPA). | Students reported their perceptions of bonding with their parents. Assessed using a 6-item questionnaire. 3 Qs asked whether students share their thoughts and feelings with their mother, would like to be like their mother, and felt like their mother really cared about them. 3 items asked whether students shared their thoughts and feelings with their father, would like to be like their father, and felt like their father really cared about them. | *Cohesion* | Results of the multiple regression analysis testing the moderating effect of parental bonding on the relationship between parental PA and youth PA. The interaction term, included in the second step of the regression analysis, was significantly related to the MVPA standard (β = 0.27, t(53) = 2.11, p = 0.04) and contributed to a significant increase in R² (ΔR²= 0.07), while the overall regression model approached significance (F(3, 53) = 2.57, p = 0.06). None of the other relationships were statistically significant. | N/A | N/A | N/A | N/A | N/A | N/A | N/A | COHESION: β = 0.27, z' = 0.277. ΔR²= 0.07, z' = 0.271. | Cohesion: Used beta value (closest to r), positive, significant, z' = 0.277 |
| ADOLESCENTS' PARTICIPATION IN SPORT ACTIVITIES AND ATTACHMENT TO PARENTS AND PEERS | Sukys, Lisinskiene, Tilndiene (2015) | Lithuania | n = 1348, girls = 716, boys = 632. Ages 12 to 16, M = 14.2, SD = 1.51. | Not disclosed/measured | Cross-sectional | Multistage sampling from 7 schools in Kaunas, Lithuania. Divided into early (12-14) and middle (15-16) adolescence for analysis. Surveys completed in classroom with researchers present. Data analyses were performed using SPSS version 19.0, and included calculating Cronbach’s alpha coefficients, descriptive statistics, independent samples t tests, Cohen’s d effect sizes, Pearson correlation coefficients, and multiple regression coefficients. Statistical significance was set at p < .05 for all tests | N/A | *Self-report:* PA was measured between if the child participated in sport vs. not participating. Sports participation assessed through self-report. Participants categorized as athletes (regular sports involvement) or nonathletes for analysis. | Of the participants, 39.2% (n = 529) were involved in competitive sport, and had been participating for an average of 3.97 years (SD = 2.43). | The Inventory of Parent and Peer Attachment-Revised (IPPA-R) measures attachment with 28 items for parents and 25 for peers, using Likert scales. Subscales include trust, communication, and alienation. Validated for children and early adolescents. | *Cohesion and Communication.* | Correlations Among Study Variables and Descriptive Statistics: Total IPPAR-R parent-attachment = .10, p<0.01. In terms of differences between adolescents’ participation in sport activities and their parent and peer attachment, our results showed that athletes scored significantly higher than nonathletes did on the subscales of overall parent attachment, M (SD) = 24.54 (4.76) vs. 23.75 (4.79), t(1,346) = 2.99, p < .05, Cohen’s d = 0.17. | Correlations Among Study Variables and Descriptive Statistics: communication and involvement in sport = .11, p<0.01. In terms of differences between adolescents' participation in sport activities and their parent attachment, our results showed that athletes scored significantly higher than non athletes did on communication, M(SD) = 16.97 (2.93) vs. 16.44(3.01), t(1,346) = 3.19, p<0.001, cohen's d = 0.18. | N/A | N/A | N/A | N/A | N/A | N/A | Cohesion: r = .10, z' = 0.1. cohen's d = .17, z' = 0.085. Communication: r = .11, z' = .11. cohen's d = 0.18, z' = 0.090. | Cohesion: just used r-value - set as standard for the study: z' = 0.1, significant positive. Communication: used r -value (standard for study): z' = .11, significant, positive. |
| Physical activity improves mental health through resilience in Hong Kong Chinese adolescents | Wing Ho, Tak Louie, Chow, Sang Wong, Ip (2015) | Hong Kong | n = 775 students. 775 (99.5%). Age M = 12.28 years (SD = 0.77). The female-to-male ratio was 1.26. | not disclosed/measured. | Cross-sectional | Study recruited Grade 7-8 students from 12 secondary schools in Hong Kong. Schools chosen for diversity in academic performance and socioeconomic areas. Parental consent obtained for questionnaire completion in class. Questionnaires measured: mental well being. Mediators: psychological pathway, social pathway, SES. Pearson correlations examined bivariate associations. Single-mediator path models compared using AIC and fit indices. Significant mediators entered into multiple-mediator model. Multi-group path model compared associations between genders. Models estimated with maximum likelihood and robust standard error estimator. Fit considered satisfactory if CFI ≥ 0.96, RMSEA ≤ 0.06, SRMR ≤ 0.09. Sensitivity analyses conducted using bootstrap standard error estimations and complete case analysis. Power analysis was done using a full path model: PA level, mental well-being, self-efficacy, resilience, school connectedness, family connectedness, gender, and SES. Sample size of 775 meets criteria for statistical power. | N/A | *Self-report:* Physical Activity Rating Questionnaire for Children and Youth (PARCY) was used to assess the student’s physical activity level. The questionnaire consists of one item that evaluates the student’s average weekly physical activity level in the past year and takes into consideration the physical activity frequency, duration, and intensity. The PARCY score is assessed on 11-point scale ranging from 0 (‘no exercise at all in the last year’) to 10 (‘doing vigorous exercise almost every day in the last year’). | PA: M = 5.41, SD = 2.68. | Resnick family connectedness scale - 13 items that are scored on a 5-pt Likert scale, which assesses the participant's closeness to and perceived support from their family and parents (e.g., feeling loved and wanted within the family). | *Cohesion* | Descriptive statistics and Pearson's correlation coefficients of the measurements: family connectedness and physical activity level = 0.06, p > .05 | N/A | N/A | N/A | N/A | N/A | N/A | N/A | Cohesion r = 0.06, z' = 0.060. | Cohesion: positive, not significant, 0.060. |
| Familial Environment and Overweight/Obese Adolescents' Physical Activity | Carbert, Brussoni, Geller, Masse (2019) | Canada | n = 172. Adolescent age: Mean = 13.1 years, SD = 1.8, Range = 11.0–16.0. Adolescent sex: Female = 55.2%. Parent age: Mean = 45.7 years, SD = 6.2, Range = 31–66. Parent sex: Female = 84.3%. Household income in CAD: $60,000 or less = 34.9%, $60,000–$100,000 = 33.7%, $100,001 or more = 31.4%. Parent education: High school or less = 17.4%, Trade certificate/diploma = 41.3%, Bachelor degree = 18.6%, More than bachelor degree = 22.7%. Parental marital status: Married or Common-law = 70.9%, Single/Widowed/Separated/Divorced = 29.1%. Ethnicity: White = 48.0%, East or Southeast Asian = 13.5%, South Asian = 12.3%, Aboriginal = 10.0%, Other ethnicity = 16.4% | not disclosed/measured | Cross-sectional | Secondary analysis of baseline data collected as part of a study elucidating the individual and household factors that predict adherence to an e-health family-based lifestyle behaviors modification intervention for overweight/obese adolescents and their family. baseline data analyzed from 172 parent/adolescent dyads for e-health lifestyle intervention. Participants recruited from various sources, including advertisements and healthcare programs. Eligibility criteria included overweight/obese status, residence in Greater Vancouver, and English literacy. Measures were recorded through self-reporting measures; parenting practices, parenting styles, FF, and PA were measured. Path analysis in Stata 13. Two models run: one with accelerometer-measured MVPA, one with self-report MVPA. Model 1 tests association between PA-related parenting practices, parental modeling, and MVPA. Final model adds authoritative, permissive parenting styles, family functioning, interaction terms. Variables standardized. Adjusted for adolescent sex, age, parental income. Residual plots and scatterplots checked for linear regression assumptions. Magnitude and significance of paths examined. | N/A | *Device-measured:* Accelerometers (GT3X/GT3X+) measured MVPA in 10-second intervals, aggregated to 1-minute intervals. Valid day defined as ≥10 hours of wear. Non-wear: no activity for ≥60 minutes. Inclusion required ≥3 valid days (1 weekend). Total MVPA minutes calculated from counts exceeding cutoffs.   *Self-report:* Seven-day PAR interview estimates MVPA for child and parent separately. Participants report MVPA activities ≥10 minutes over the past week. Intensity levels (leisure walking, moderate, very hard) defined. Probing ensures comprehensive data collection. MET values assigned using Compendium of Energy Expenditure for Youth. MVPA defined as activities ≥4 MET. Total MVPA minutes divided by 7 for daily average. | mean = 2.4 ± 0.8 , range = 1–4. Measured MVPA (min/day): 2.4 ± 0.8 1–4, range = 1.0-103.1. Self-reported MVPA (min/day): 68.9 ± 89.7, range = 0.0–488.6 | The Family Adaptability and Cohesion Evaluation Scale IV (FACES IV) [32] assessed family functioning. The original measure comprises 42 items assessed on a five-point Likert scale ranging from strongly agree to strongly disagree. The measure contains six subscale: balanced cohesion (e.g., “feeling very close”), balanced flexibility (e.g., “able to adjust to change”), enmeshed (e.g., “spending too much time together”), disengaged (e.g., “avoid contact with each other”), chaotic (e.g., “never seem to get organized”), and rigid (e.g., “rules for every possible occasions”). These six subscales measured two overarching dimensions of cohesion and flexibility | *General Family Functioning (i.e., family relationship)* | N/A | N/A | N/A | N/A | N/A | N/A | N/A | Association between physical activity (parenting practices of physical activity) and adolescents' accelerometer and self-report of moderate-vigorous physical activity (standard coefficient [SC]): family functioning and accelerometer MVPA time = final model (SC(SE)) = −0.05 (0.09), p = 0.53. Self reported MVPA time final model = 0.12 (0.08), p = 0.15. | SC = -0.05, z' = -0.050. SC = 0.12, z' = 0.121. | General FF: used accelerometry time - more accurate vs. self-report: z' = -0.050, non significant negative. |
| Mother-child engagement in sports and outdoor activities: Intensive mothering, purposive leisure, and implications for health and relationship closeness | Knoester, Fields (2020) | USA | n = 3252. female child: 47%. Mother’s age: M = 34.52, SD = 6.01, Min = 23, Max = 56. High school education: 21%, Some college education: 42%, College or more education: 17%. Ethnicity: Black ethnicity: 49%, Latina ethnicity: 26%. | cohabitating with father: 10%, living apart from father: 59%, new partner: 20%. Number of children: M = 2.93, SD = 1.34, Min = 1, Max = 6 | cross sectional - however did use data from different waves of a longitudinal study | Data sourced from Fragile Families and Child Wellbeing Study (FFCW), a longitudinal birth cohort study of 4898 children born in the US (1998-2000). Primarily uses data from when children were nine years old (W5), conducted between 2007 and 2010. Sample includes 3252 mothers reporting on mother-child interactions in sports and outdoor activities. Descriptive statistics revealed the frequency of mother-child engagement in sports/outdoor activities. Ordinary Least Squares (OLS) regression analyzed associations between engagement frequency and various outcomes, including parenting perceptions, health, and relationship closeness. Logistic regression predicted child's organized sports participation. These statistical methods were employed to assess associations with demographics, SES, and family dynamics. | Data was derived from the Fragile Families and Child Wellbeing Study (FFCW). The FFCW is a longitudinal birth cohort study of the families of 4898 children born in the US between 1998 and 2000 | *Self-report:* Three variables indicate engagement in sports or outdoor activities. Mother's reports of children's participation in organized athletic activities outside of school hours in the past year (1 = yes), in order to consider a child's organized sports experiences. |  | reports of a mother’s and their child's health and relationship closeness (both mother and child report). | *Cohesion* | Results from OLS regression predicting mother-child relationship closeness: mother's reports (Model I) and child's organized sports participation = b = 0.05, p<0.05 SE = 0.02. child's reports (model 2) and child's organized sports participation = b = 0.08, p<0.01 SE = 0.03. | N/A | N/A | N/A | N/A | N/A | N/A | N/A | Cohesion: mother's reports and child PA = b = 0.05, z' =0.05. child's reports and PA = b = 0.08, z' = 0.08 | Cohesion: taken mother report (standard for study - more reliable): z' = 0.05, significant, positive. |
| Physical Activity, Screen Time, and Mood Disturbance Among Chinese Adolescents During COVID-19 | Xiao, Yan, Zhao (2021) | China | Male n = 862, female n = 818. Grade: 7th n = 609, 8th = 386, 9th = 116, 10th = 271, 11th = 253, 12th = 45. | Not disclosed. | Cross sectional | A description of the survey and the survey link were sent to students in grades 7 to 12 and their parents in the middle-high school via WeChat, a popular social media application (app) in China. Different WeChat groups were organized by grades and classes. All data were entered, coded, and analyzed using SPSS 26.0. Mood disturbance score, physical activity score, types of physical activity, online study time, and other screen time were reported descriptively. Hierarchical regression analyses were performed to explore factors that predicted mood disturbance and relationships with parents. | N/A | *Self-report:* physical activity participation was measured by the Leisure-Time Exercise Questions. This measure estimated student's participation in weekly PA during the stay-home period in a range of mild (e.g., easy walking), moderate (e.g., fast walking), and strenuous (e.g., running) forms. Based on this information, exercise metabolic (MET) units were calculated according to the following formula: [(Mild × 3) + (Moderate × 5) + (Strenuous × 9)]. Participants were also asked whether they had undertaken at least 150 minutes of physical activity each week. | PA score: Male M=69.73(SD=71.13), female M = 61.44 (SD = 65.70). Grade: 7th M = 70.15 (SD = 65.55), 8th M = 70.29 (SD = 67.53), 9th M = 70.31 (SD = 83.83), 10th M = 59.63(SD = 73.29) 11th = M = 52.05 (SD = 62.80), 12th M = 65.37 (SD = 70.57). A total of 69.5% (males = 72.4%;  females = 66.4%) of participants undertook at least 150 minutes of physical activity each week during lockdown. Male students had signifi cantly higher physical activity scores than females (t[1,578] = 2.40, p < 0.05). Students in lower grades were also more physically active than those in higher grades (F[5, 1,674 = 3.21, p < 0.01). Specifically, 7th grade students had a higher physical activity score than 10th (p < 0.05) and 11th (p < 0.01) grade students; 8th grade students were more active than 11th grade students (p < 0.01); and 9th  grade students were more active than 11th grade students (p < 0.01). | Measured predictors of conflicts with parents. | *Conflict* | N/A | N/A | In model 3, PA participation did not predict conflict with parents, however after controlling other variables, undertaking at least 150 minutes of PA each week predicted fewer conflicts with parents (model 4): Hierarchical regression analysis on conflicts with parents: participating in >150 minutes of PA = -0.21, p<0.001. | N/A | N/A | N/A | N/A | N/A | Conflict: β = -0.21, z' = -0.213 | Conflict: negative, significant, -0.213 |
| Physical activity level, mediterranean diet adherence, and emotional intelligence as a function of family functioning in elementary school students | Melguizo-Ibanez, Viciana-Garofano, Zurita-Ortega, Luis Ubago-Jimenez, Gonzalez-Valero (2020) | Spain | The sample was formed by 189 students from the province of Granada with self-reported ages between 11 and 12 years (M = 11.45 ± 0.31). Sample distribution was homogenous, with 101 (53.4%) being male and 88 (46.6%) being female. | Not disclosed. | descriptive cross-sectional | An ad-hoc questionnaire was used for data collection, which was designed to collect sociodemographic variables such as sex and age. Data on emotional intelligence, diet type, and family functioning was collected. In order to complete the data collection processes, the different schools selected via convenience sampling were contacted. Once permission was received from the schools, an information pack was developed that was targeted towards students’ legal guardians so that students could participate in the study while ensuring anonymity at all times. Researchers were present throughout the data collection processes in order to resolve any doubts arising during questionnaire completion. The normality of the data and homogeneity of the variables were examined according to the Kolmogorov–Smirnov test. Following this, descriptive analysis was conducted via an analysis of frequencies and means. For the comparative analysis, contingency tables and Student’s t-test for independent samples were used. Differences between participants were determined via Pearson chi-square analysis. One-way analysis of variance (ANOVA) comprising a single factor with Bonferroni post-hoc analysis was also used to conduct between-variable analysis. Likewise, bivariate Pearson correlations were conducted. The level of significance was set at p < 0.05 and p < 0.01. The magnitude of differences (effect size; ES) was obtained according to standardised measures of Cohen’s d. This value is interpreted as being null (0–0.19), small (0.20–0.49), medium (0.50–0.79), or large (≥0.80). Finally, 95% confidence intervals (95% CI) were calculated for each effect size. | N/A | *Self-report:* ad-hoc questionnaire designed to collect sociodemographic variables also estimated the level of physical activity engaged in outside of timetabled school hours. Responses were categorised as “yes” or “no” according to whether they met the recommended physical activity levels proposed by the World Health Organization | most individuals were highly physically active, with 87.8% (n = 166) engaging in extra-curricular physical activity, while only 12.2% (n = 23) reported not engaging in this behaviour | APGAR questionnaire was used to measure FF. Specifically, the version adapted into Spanish by Suarez and Alcalá. This questionnaire includes a total of five questions about the relationship maintained between  students and their family. In order to obtain the final score, the scores provided in relation to each value indicated on a three-point Likert type scale are considered. In this sense, 0 relates to “almost never”, 1 equates to “sometimes”, and 2 describes “almost always”. Finally, all of the questions are summed together in order to obtain a final score. | *General Family Functioning (i.e., family relationship)* | N/A | N/A | N/A | N/A | N/A | N/A | N/A | Relational analysis of the variables describing family functioning: PA: and FF = p = 0.677. Normal FF: 11.8% don't engage in PA, 88.2% engage in PA; Moderate FF: 17.6% don't engage in PA, 82.4% engage in PA; Severe FF: 0.0% don't engage in PA, 100% engage in PA | N/A - no effect size. | General FF: non significant, positive - from p-value. |
| Levels of Physical Activity, Family Functioning and Self-Concept in Elementary and High School Education Students: A Structural Equation Model | Zurita-Ortega, Alonso-Vargas, Puertas-Molero, Gonzalez-Valero, Ubago-Jimenez, Melguizo-Ibanez (2023) | Spain | The sample size consisted of 706 participants. The average age of the participants ranged from 11 to 15 years old (Age = 12.01; SD = 1.13). The gender distribution was 56.1% (n = 396) male and 43.9% (n = 310) female | Not disclosed. | cross sectional | Prior to carrying out this research, a systematic search was carried out on the subject to study the most reliable instruments for data collection, as well as to guide this research. Then, once the sample group had been selected, the different educational institutions were contacted and invited to collaborate in this research. Once a favourable response was obtained from the educational establishments, the research team drafted an informative letter from the department of Didactics of Musical, Plastic and Bodily Expression, which was addressed to the legal guardians of the children, authorising their sons and daughters to participate in the study. The researchers were present during data collection, helping to resolve any doubts that arose during the completion of the questionnaires. To avoid possible random responses, one question was duplicated, eliminating all participants where this question did not match. In this case, a total of 12 responses were eliminated. In this case, the comparative analysis was carried out using the T-Student test for independent samples. For the study of statistically significant differences, the Pearson Chi-square test was used, establishing the level of significance at p ≤ 0.05. For the effect size, Cohen’s standardised d measure was used. | N/A | *Self-report:* Whether or not student participates in more than 3h of PA weekly. | not disclosed. | APGAR questionnaire was used to measure the degree of family functioning. This is made up of a total of five questions, including  “do you feel that your family loves you?”, through which the degree of family functionality is measured. | *General Family Functioning (i.e., family relationship)* | N/A | N/A | N/A | N/A | N/A | N/A | N/A | participants who do meet the physical sport criterion show higher scores in family functioning (2.80 ± 0.50). comparative study between time spent in physical activity and family functioning: More than 3h of PA = n = 340, M = 2.80, SD = 0.50, sig = ≤0.05. Less than 3h = n = 366, M = 2.71, SD = .57, sig = ≤0.05; Cohen's d = .17 Comparative study between time spent in PA and FF: FF and more than 3 h = N = 340, M = 2.80 SD = 0.50. Less than 3h = N = 366, M = 2.71, SD = .57, sig = <0.05. | General FF: cohen's d = .17, z' = 0.085 | General FF: significant, positive, 0.085. |
| Family functioning: associations with weight status, eating behaviors, and physical activity in adolescents | Berge, Wall, Larson, Loth, Neumark-Sztainer (2012) | USA | girls n = 1,486. boys n = 1,307. Ethnicity/race: White girls =16.7%, White boys = 21.2%, Black girls =28.9%, black boys = 29.0%, Hispanic girls =17.2%, Hispanic boys= 16.5%, Asian girls =19.9, Asian boys =19.9%, Native American girls= 3.6%, Native American Boys = 3.7%, Mixed/other girls =13.7%, Mixed/other boys = 9.7%. Socioeconomic status: Low girls = 42.8%, low boys =33.4%. Low middle girls =20.9%, low middle boys= 21.8%, Middle girls = 16.2%, middle boys= 17.7%, High middle girls =11.2%, high middle boys= 13.8%, High girls = 5.9%, high boys= 8.8%, Missing girls =3.0%, Missing boys =4.6%. Age in years girls = M= 14.4 (SD=1.9), boys = M = 14.5 (SD=2.1) | not disclosed. | Cross sectional. | EAT 2010 (Eating and Activity in Teens) is a population-based study designed to assess dietary intake, physical activity, weight control behaviors, and weight status in adolescents. Surveys and anthropometric measures were completed by 2,793 adolescents from 20 public middle schools and high schools in the Minneapolis/St. Paul metropolitan area of Minnesota during the 2009 – 2010 academic year. Trained research staff administered surveys and measured  adolescents’ height and weight during selected required health,  physical education, and science classes. Measurements were  completed in a private area, and surveys were administered  during two class periods that were typically 45–50 minutes.  After survey completion, participants were given a $10 gift card | N/A | *Self-report:* Physical activity questions were adapted from the Godin Leisure-Time Exercise Questionnaire. Adolescents were asked: “In a usual week, how many hours do you spend doing the following activities: (1) strenuous exercise (e.g. biking fast, aerobics, jogging, swimming laps, soccer, rollerblading) and (2) moderate exercise (e.g. walking quickly, easy bicycling, skiing, dancing, skateboarding, snowboarding).” | Moderate-to-vigorous physical activity hours per week = girls = M=5.0 (SD =4.4), boys = M = 6.7 (SD = 4.9) | Six items were drawn from the general functioning scale of the Family Assessment Device to measure overall family functioning. The general functioning scale on the Family Assessment Device measures structural, organizational, and interaction patterns of the family, including problem solving, communication,roles, affective responsiveness, affective involvement, and behavior control among family members. Adolescents were asked,“How strongly do you agree with the following statements? For these questions, think about your family in general (including your parents and your brothers and sisters). . . [Strongly disagree, Somewhat disagree, Somewhat agree, Strongly agree] (a) Family members are accepted for who they are; (b) Making decisions is a problem for the family; (c)We don’t get along well together; (d)  We can express feelings to each other; (e) Planning family activities is difficult because we misunderstand each other; (f) We confide in each other (By ‘confide’ we mean to trust your family members enough to tell them something that is important to you).” Responses were assigned values from 1 to 4, and all statements were converted to the positive form before the values were summed. The responses for this scale ranged from 6 to 24, with higher scores representing higher family functioning (scale alpha = .70) | *General Family Functioning (i.e., family relationship)* | N/A | N/A | N/A | N/A | N/A | N/A | N/A | Relationship between family functioning and adolescent girls BMI and health behaviour outcomes: physical activity: MVPA (hrs/wk) and FF score at 5th percentile = 4.65, FF score at 95th percentile = 5.12, standardized β = 0.03, SE = 0.03, t-value = 1.17, p-value = .207. Relationship between family functioning and adolescent boys' BMI and health behaviour outcomes: physical activity: MVPA (hrs/wk) and FF score at 5th percentile = 5.81, FF score at 95th percentile = 7.60, standardized β = 0.10, SE = 0.03, t-value = 3.71, p-value = <.001. | Relationship between family functioning and adolescent girls BMI and health behavior outcomes: β = 0.03, z' = 0.030. Relationship between family functioning and adolescent boys' BMI and health behavior outcomes: β = 0.10, z' = 0.100. | General FF: taken average of boys and girls z' values: z' = 0.065. Considering that 50% of values are considered significant - study would be considered positive and significant. |
| The effects of connectedness on health-promoting and health-compromising behaviors in adolescents: Evidence from a statewide survey | Yang, Tan, Cheng, 2014 | USA | n = 46,588 ninth and eleventh-grade students. n = 10,210 Asian American, n = 3759 Pacific Islander, n = 32,619 White American. | not measured/disclosed. | cross sectional | Study data was acquired from the 2006-2008 high school questionnaire of the California Healthy Kids survey. students in the 9th and 11th grades were administered paper and pencil versions of the scales. Sampling procedures were based on the size of school districts. In districts with more than 900 students per grade, there was a total of 900 students randomly selected per grade. In districts with less than 900 students per grade, a census of students was selected. The study utilized IBM SPSS Statistics 20 for data analysis, acknowledging the non-normal distribution and skewness of variables in behavioral and social sciences. To address this, they dichotomized variables following Farrington and Loeber's recommendations, simplifying presentation while potentially losing some information. Generalized Estimating Equations (GEEs) were employed to control for clustering effects in the multi-level data, particularly for binary outcomes, using logistic link functions. The analysis focused on the effects of connectedness across different domains on health behaviors in Asian Americans (AAs), Pacific Islanders (PIs), and White Americans (WAs), controlling for age, sex, and school-level socioeconomic status (SES). Model stratification by race/ethnicity was chosen over interaction terms to prevent model overspecification. Chi-square tests were also conducted to compare outcome variables across racial/ethnic groups. | N/A | *Self-report:* Participants indicated the number of days they exercised (e.g., strength training and aerobic activity) in the past week. Participants rated three items on an 8-pt scale ranging from A (0) through H (7). | Not specifically reported | *Cohesion:* Family connectedness measures one's caring relationships in family dynamics, opportunities to participate in the home environment, and parents' expectations for success. Participants rated nine items on a 4-pt Likert scale ranging from A (not at all true) through D (very much true). The average was then take to indicate family connectedness, with higher scores denoting higher family connectedness. | *Cohesion* | Generalized estimating equation analyses predicting physical activity from control and connectedness variables: Asian American and Family Connectedness: B = 0.06, Std.error = 0.07, 95% Wald confidence interval =-0.09 to 0.19, wald chi square = 0.54. Pacific Islander and family connectedness B = 0.11, Std.error = 0.12, 95% Wald confidence interval =-0.12 to 0.34, wald chi square = 0.84. White American and family connectedness: B = 0.03, Std.error = 0.05, 95% Wald confidence interval =-0.06 to 0.12, wald chi square = 0.41. | N/A | N/A | N/A | N/A | N/A | N/A | N/A | Cohesion: Asian American, β = 0.06, z' = 0.060. Pacific Islander β = 0.11, z' =0.11. White American β = 0.03, z' = 0.03. | Cohesion: taken average of all ethnicities z' values: z' = 0.067, positive not significant. |
| Socio-economic position and adolescents' health in Italy: the role of the quality of social relations | Zambon, Lemma, Borraccino, Dalmasso, Cavallo (2006) | Italy | n = 4386. 51.555 = female, 23.75% = 11 years old, 37.23% = 13 years old, 28.02% = 15 years old. | not measured/disclosed. | cross sectional | The sample was chosen from a systematic sample of school classes divided into five geographical strata (north-west, north-east, centre, south, islands). Questionnaires were self-administered, and were sent to 314 school classes. The methods of data collection and the questionnaire followed the HBSC study's international research protocol. Interpersonal relations analyzed by age, gender, social class, geography. Health behaviors tested vs. Family Affluence Scale (FAS) via ordinal gamma index. Logistic model fitted with health behaviors as dependent, social relations as independent variables. Age, gender, socio-economic position controlled. Dependent variables dichotomized (e.g., daily smoking vs. less often). Independent variables dichotomized based on ease of communication for parents and friends, and perceptions of teacher fairness. | N/A | *Self-report:* Frequency of PA was measured through self report. | not reported. | Under 'assessment of quality of social relations'. Assessed 'how is it for the child to talk to their mother and father' with a 5-category scale: 'very easy', 'easy', 'difficult', 'very difficult', 'I don't have or see this person'. | *Communication* | N/A | OR (95% CI) for the effect of the relations with father, mother, best friend, and teachers on four unhealthy behaviours (smoking daily, drinking alcohol once a week or more often, doing physical exercise for 1 h twice a week or less often, having used cannabis), controlled by gender, age, and FAS: father: easy relation and low PA = 1.00. father difficult relation = 1.03 (0.89-1.20), p = significant. mother easy relation = 1.00. mother difficult relation = 0.96(0.81-1.15), p = significant | N/A | N/A | N/A | N/A | N/A | N/A | Communication: easy relation father OR = 1.00, z' = 0. difficult relation father OR = 1.03, z' = 0.008. mother easy relation OR = 1.00, z' = 0. difficult relation mother OR = 0.96, z' = -0.011. | Communication: used difficult relation value - easy relation is the scaler value. Took average of father and mother z' values: z' = 0.5095. positive significant. |
| 儿童体育活动的家庭代际传递:家庭亲密度的中介效应 (Intergenerational Family Transmission of Children's Physical Activity Medicating Effect of Family Closeness). | Yonghe (2021) | China | Child age = 11.95± 2.091 years. n = 327 boys and 335 girls; father's age = 41.67± 3.942 years, junior high school education or below = 138, high school/college education = 209, university/college degree or above = 315. mother’s age = 38.92±2.787 years, junior high school education or below = 144, high school/junior college = 221, university/college degree or above = 297. | not measured/disclosed. | Cross-sectional | combination of convenience and stratified cluster sampling was used in elementary and junior high schools. The study utilized Liang Deqing's Physical Activity Rating Scale (PARS) and Fei Lipeng et al.'s Family Cohesion Scale (FCS) to assess physical activity and family intimacy respectively. Data from children, fathers, and mothers were imported into Excel 2016, screened for validity, and then analyzed using SPSS24.0. Analysis included parametric testing, exploratory and confirmatory factor analyses, reliability analysis, Spearman correlation, and regression. Bootstrap method (version 3.5) was used to assess family intimacy's mediating effect on the relationship between parental activity and children's activity. | N/A | *Self-report:* Leung's Physical activity rating scale was used. Parents, mothers and children responded individually to the questionnaire. This scale was designed to examine the intensity, duration, and frequency of the respondents' participation in PA in the past month. Five levels with duration, intensity and frequency scored on the scales. Leung Tak Ching's measurement formula was used to obtain the measurement (amount of PA of the participant = intensity x time). Frequency was based on a score of <19 for small exercise, 20 for moderate exercise. The activity level was categorized into 3 grades of 42 points. |  | Family closeness scale was used as a measure of family functioning. 12 questions, and 4 reverse questions (e.g., in our family, recreational activities are done by the whole family). Each item was scored using the Likert 5-point scale from never to always. Total family intimacy score was obtained and used. | *Cohesion* | The direct impact of degree on children's physical activity was measured between Child PA and family intimacy: r = 0.449. There is a significant positive correlation between family intimacy (P<0.001). Family intimacy (β= 0.446) had a significant positive impact on children's physical activities (P<0.001). | N/A | N/A | N/A | N/A | N/A | N/A | N/A | Cohesion: Child PA and cohesion r = 0.449, z' =0.483. | Cohesion: significant, positive, 0.0483. |
| Psychosocial and health behavioural characteristics of longitudinal physical activity patterns: a cohort study from adolescence to young adulthood | Aira, Vasankari, Heinonen, Korpelainen, Kotkajuuri, Parkkari, Savonen, Toivo, Uusitalo, Valtonen, Villberg, Vaha-Ypya, Hokko (2023) | Finland | Baseline: (n = 583, mean age 15.5, SD 0.6). Follow-up measurement: (n = 371, mean age 19.4, SD 0.6). 62% of participants lived in families w/ high affluence. | not disclosed/measured | Observational cohort study | Data drawn from HPSC study, assessed PA patterns and correlates in Finnish adolescents. Data collected via surveys and medical examinations. PA measured via accelerometers. Longitudinal PA patterns identified using k-means. Exposure variables included sports club participation, active commuting, psychosocial factors, health behaviors (alcohol, snuff, toothbrushing, breakfast, school meals, fruit/vegetable intake, sweets/drinks), and sleep duration. Researchers assessed differences in physical activity (PA) patterns using cross-tabulations, Chi-square/Fisher’s exact tests for categorical variables, and Kruskall-Wallis test with post hoc Dunn’s test for continuous variables. Multinomial logistic regression calculated odds ratios (ORs) with 95% confidence intervals (CIs) for associations between exposure variables and PA pattern membership. Activity maintainers and increasers were combined due to small sample size (n=20) and favorable health outcomes. Models were developed separately for baseline (mean age 15) and follow-up (mean age 19), adjusting for measurement interval and change in device wear-time. SPSS version 26 was used for analysis, with significance set at p<0.05. | Data was drawn from the health promoting sports club study conducted in the years 2013 to 2014 and 2017-2018. | *Device-measured:* 54 adolescents (60% females) provided valid accelerometry data for both measurements (swimmers excluded: n=22, at least four days, 10 h/day). PA was measured using a Hookie accelerometer (AM20 Activity Meter, Hookie Technologies Ltd., Helsinki, Finland). The data were analysed in units of 6 seconds’ duration. The PA analysis was based on mean amplitude deviation analyses (MAD), calculated from a resultant tri-axial raw acceleration signal, and converted to metabolic equivalents (METs). | the inactivity maintainers were mostly female (73%), decreases from high to moderate were mostly males (19% females). | Communication with parents: The study participants were asked how easy it was for them to talk to their mother about things that really bothered them. The response options were: very easy, easy, difficult, and very difficult, with the additional response option I don’t have or see this person. Identical questions were posed regarding communication with the father and (if applicable) stepfather and stepmother. Separate dichotomised variables (easy vs. difficult) were formed for communication with (1) the mother and (2) the father. The latter also included talking over difficulties with one’s stepfather in cases where the respondent did not have a father (baseline n=3, follow-up n=5). Cases were excluded where there was neither mother nor stepmother (baseline n=3, follow-up n=8) (and similarly neither father nor stepfather, baseline n=14, follow-up n=16). | *Communication* | N/A | Fig 1 Analysis stemming from exposure variables presented at baseline (mean age 15 ): Odds ratios (log-scale) with their 95% confidence intervals, calculated via a multinomial logistic regression analysis predicting belonging to longitudinal PA patterns. Comparisons of inactivity maintainers and decreaser groups with the combined group of activity maintainers and increasers. Adjusted for the measurement interval and the change in device wear time between baseline and follow up. *Difficult to talk with father:* inactivity maintainers p = 0.025, decreasers from moderate to low PA = 0.002, decreasers from high to moderate PA = 0.329. *Communication difficulties with one's father* OR = 2.4;  CI: 1.1–5.1) at age 15 were associated with increased odds of being an inactivity maintainer as compared to membership of a group with favourable PA development (p = .025). *Communication difficulties with one’s father at age 15* (OR = 3.3; CI: 1.6–7.1) was related to belonging to decreasers from moderate to low PA (p = .002). | N/A | N/A | N/A | N/A | N/A | N/A | Communication difficulties with one's father: OR = 2.4, z' = 0.239. Communication difficulties with one’s father at age 15: OR = 3.3, z' = 0.323. | Communication: taken average of initial and age 15 z' values: z' = 0.281, significant, positive. |
| Don't stop, don't stop: physical activity and adolescence | Suris, Parera (2005) | Switzerland | n = 6928. PA group n = 4185 (43.5% = females). Inactivity group n = 2743 (68.9% = females). |  | cross sectional | The study utilized data from the Catalonia Adolescent Health Survey 2001, involving in-school adolescents aged 14 to 19. Out of 97 selected schools, 84 participated. A self-administered questionnaire with 92 questions was used. 6,952 valid responses were analyzed. The physical activity group included 4,185 subjects (43.5% females), while 2,743 (68.9% females) were classified as inactive. Variables measured included personal, family, school, and lifestyle factors, including health perception, parental smoking, academic performance, and substance use. SPSS 10.0® for Windows® was used. Chi-square and t-tests compared variables. Bivariate analysis included Odds Ratios (OR) with 95% CI for categorical variables. Multivariate analysis used nonconditional multiple regression with significant variables from bivariate analysis plus age, done separately by gender due to age and gender-dependent physical activity changes. | Data was obtained from the Catalonia Adolescent Health Survey 2001, a cross-sectional study of in-school adolescents aged 14 to 19 years. | *Self report:* Questionnaire referred to the practice of PA (sports, dancing, etc.) during leisure time (outside school hours). All those who reported that they exercised at least once a week were included in the PA group (N=4,185; 43.5% females) and the remaining 2,743 (68.9% females) in the inactivity group. | All those who reported that they exercised at least once a week were included in the physical activity group (N=4,185; 43.5% females) and the remaining 2,743 (68.9% females) in the inactivity group. There were significantly more males (73.5%) than females (49.0%), who practiced some kind of PA. | FF measured as a variable under category "family". questions on top of relationship with father (scale from 1-10), and relationship with mother (scale from 1-10). | *General Family Functioning (i.e., family relationship)* | N/A | N/A | N/A | N/A | N/A | N/A | N/A | Bivariate analysis by physical activity - males: Group with PA = Y: relationship with father = 7.75+/-2.0, Group with PA =N: relationship with father = 7.29+/-2.1 p = 0.000. Group with PA = Y: Relationship with mother = 8.12+/-1.7, Group with PA = N: 7.89+/-1.7, p = 0.001. Multivariate analysis. Dependent variable: PA (Y/N) - Males: Relationship with father = -0.154, OR(95%CI) = 1.10(1.04/1.16), p=0.001. Relationship with mother = -0.007, OR(95%CI) = 0.99(0.93/1.06). Bivariate analysis by physical activity - females: Group with PA = Y: relationship with father = Ί .36+/-1-2 .1, Group with PA =N: relationship with father = 7.12+/-2.3, p = 0.001. Group with PA = Y: Relationship with mother = 8.14+/-1 .7, Group with PA = N: 8.02+/-1.8, p = 0.038. Multivariate analysis. Dependent variable: physical activity (Y/N) - females: relationship with father = 0.018, OR (95%CI) = 1.62 (1.38/1.89), p = 0.000. Relationship with mother = 0.008, OR (95%CI) = 1.01 (0.96/1.05). adolescents who exercised had a significantly better relationship with their father, and mother. In the multivariate analysis, the only variables that remained significant were age (inversely), the relationship with their father (but not with their mother). | General FF: Multivariate: Males: relationship w/ father OR = 1.10, z' = 0.026. relationship w/ mother OR =0.99, z' = -0.003. Multivariate: Females: relationship w/ father OR = 1.62, z' = 0.133. relationship w/ mother OR = 1.01, z' = 0.003. | General FF: taken average of relationship w/ mother and father across males and females: z' = 0.0398. significant positive. |
| The relationship among characteristics of the family environment and behavioral and physiologic cardiovascular risk factors in parents and their adolescent twins | Riley-Lawless (2000) | USA | n = 63 families analyzed for the present study. mothers age: M = 41.2 (SD = 5.09). Mean education = 13.8 years, SD = 2.44. Father age: M = 42.7 years (SD = 2.88). Twin age: M = 13 year (SD = 1.87). Median family income = 40,000$. | n = 63 mothers, n = 50 fathers, n = 63 sets of twins. 79% = four family members in family unit. 21% = three family members in the family unit. | Secondary data analysis study | Families suggested for selection were contacted by phone, and screened for interest and eligibility. This study performed a secondary analysis of data previously collected from a longitudinal study. Specifically, phase 2 of the longitudinal study was used - registered nurses collected the data during two home visits between 1.5 to 2.5 hours. Anthropometric, fasting blood samples, and questionnaires were completed during these visits. Family environment, PA behavior, cigarette smoking, total cholesterol, blood pressure, and obesity were all measured as risk factors/outcome variables. A power analysis was conducted at a significance level of 0.05. Researchers used SPSS 6.0 to conduct their data analysis. Descriptive statistics were examined for all study variables. Central tendency, variability, skewness, and kurtosis were calculated and examined for the independent variables cohesion and conflict. Internal consistency reliabilities were calculated. Frequency, variability, and distribution were calculated for the dependent variables. Correlation matrices were constructed for each independent variable and dependent variable for each family member (significance of p<0.05). | Study is a secondary analysis of data from the Delaware Valley Twin Study, a longitudinal, observational, epidemiologic investigation of psychosocial, behavioral, and physiological risk factors in twin children and their parents. | *Self-report:* PA was measured by self-report items adopted from previous studies. Parental PA was measured through this instruction: "Circle all the items below which most closely describe your weekly levels of PA, and write the approximate number of hours you spend weekly in each circled activity". Responses were coded as either 1 = inactive, 2 = low active, 3 = active, 4 = high active. Child PA was measured through this question: "How do you spend your leisure time?" - twins selected one of four categories. 1 = less active, 4 = most active. | the percentage of inactive twins was similar in both groups (25% vs. 26%), but a larger percentage of twins in group 1 were in the highest activity group (54% vs. 44%). | The family environment scale was used to measure dimensions of the family environment. FES was developed to assess the perceived social climate or "personality" of the home environment. 10 subscales, with 9 T/F questions in each subscale, that measure the social environment of families. Specifically, cohesion and conflict were measured for this study. The higher the score, the more family cohesion/conflict. | *Cohesion and Conflict* | In both groups, cohesion correlated positively with physical activity. Spearman correlation with cohesion and PA: Twin 1 = .25 (p > .05), Twin 2 = .18 (p > .05). In one twin group, cohesion and PA just missed statistical significance, and in the other twin group, there was no significance or near significant results. | N/A | Spearman Correlation with conflict and PA: Twin 1 = 0.01 (p > .05), Twin 2 = -0.003 (p > .05). | N/A | N/A | N/A | N/A | N/A | Spearman Correlation with conflict and PA: Twin 1 = 0.01, z' = 0.010. Twin 2 = -0.003. z' = -0.003. Spearman correlation with cohesion and PA: Twin 1 = .25, z' = 0.255. Twin 2 = 0.18, z' = 0.182. | Conflict: taken average of twin 1 and twin 2 z' values: z' = 0.0035, positive, not significant. Cohesion: Taken average of twin 1 and twin 2 z' values: z' = 0.437, positive, not significant. |
| Exploring Chinese children's physical and sedentary activity | Chen, Unnithan, Kennedy (2006) | Taiwan | Child participants n = 153. Age = 8-10 years. girls = 55.2%, boys = 44.8%. mothers: USA = 68, Taiwan = 95. USA mother age M = 42.1, SD = 3.8 years, Taiwan mother age M = 35.9, Sd = 3.9 years. USA mothers years of education M = 13.4, Sd - 4.2. Taiwan mothers years of education M = 11.1, SD = 2.8. Taiwan mother occupation: housewives = 42.1%. USA mother occupation: housewives = 28%. Taiwan mothers marriage status: married = 93.7%. USA mothers marriage status: married = 95.6%. Taiwan family annual income: less than 12,000$ USD = 4.7%. USA family annual income: less than 10,000$ USD = 6.0%. | not measured/disclosed. | Cross sectional | Participants recruited from one elementary school located in Southern Taiwan, participants in the USA were recruited from 2 Chinese-language schools. Data was collected between Sep 2001 and Feb 2002. BMI, family demographics, self-identity acculturation, family functioning, attitudes toward child-rearing, and food frequency were all measured. Data analysis consisted of t-tests, chi-squares, e-way analysis for age, sex, and country with variables such as FAD, ATCRS, FFQ, SAPAC, BMI and SCSI used. A step-wise multiple linear regression model was also performed for physical and sedentary activities. | N/A | *Self-report:* Self-administered physical activity checklist (SAPAC) was used and translated into Chinese. SAPAC was originally designed for a study on cardiovascular health of children - children were asked to recall activities that occurred the previous day such as before school, during school, and after school. The amount of time in minutes they spent engaged in activity during these 3 periods was estimated and reported. Additionally, reports of MVPA, VPA, or LPA was reported to estimate METs. | MVPA Minutes: Taiwan (n=95): 98.5 (84.55), USA (n=68): 83.58(69.66) | The family assessment device was used to measure family functioning in 6 domains. This device was based on the McMaster model, which in turn was derived from systems, roles and communication theories. 7 subscales: problem-solving, communication, roles, affective responsiveness, affective involvement, behavior control, and general functioning. A higher score indictive of poor family functioning. | *Affective environment* | N/A | N/A | N/A | N/A | A multiple linear regression model identified 2 variables that contributed to differences in the Taiwanese children's MVPA METs (MVPA METs). These 2 variables were better family affective responsiveness (sr^2 = 0.06, F = 5.2, p =0.03). | N/A | N/A | N/A | Affective Environment: sr^2 = 0.06, z' = 0.250 | Affective environment: positive, significant, 0.250. |
| ŚRODOWISKO RODZINNE A POZIOM AKTYWNOŚCI FIZYCZNEJ DZIECI or Family Environment and the Level of Physical Activity of Children | Ostrowska-Karpisz, Siekanska, Wojtowicz (2018) | Czech Republic | The study involved 65 families (191 people). Children (M age = 13.3 years). | not disclosed/measured | June - September 2016. Cross sectional. | The study involved 65 families (191 people). Children (M age = 13.3 years) had to be 10-15 years old, engage in extracurricular physical activity, and have parental consent. Parents (M age = 42.8 years) only needed to consent. Parents were categorized as physically active or inactive. The research was conducted in Krakow from June to September 2016. Participants were recruited through schools, gyms, and pools. Children were surveyed in the presence of the researcher, while some parents completed surveys at home. Participation was voluntary and anonymous, with informed consent obtained. The study used four tools The study used four tools: 1. Athlete's Family Environment Questionnaire (KŚRS): 83 items across 9 scales on family environment, rated 1-5. 2. International Physical Activity Questionnaire (IPAQ): 7 questions on physical activity, expressed in MET units. 3. Parent Questionnaire: Self-designed, 16 questions on demographics, physical activity, and family environment. 4. Child Questionnaire:** Self-designed, 13 questions on demographics, physical activity, and future plans, allowing comparisons with parents. Statistical analyses used Statistica 13 and included descriptive statistics, Pearson's r correlations, regression analysis, Student's t test, ANOVA, Tukey post-hoc test, and Shapiro-Wilk test for normality. | N/A | *Self-report:* The International Physical Activity Questionnaire was used. It consists of 7 questions related to the time the participants spend sitting, walking, moderate, and intense PA. Only activities longer than 10 minutes were counted. | High PA = 55.38%, sufficient PA = 38.47%, insufficient PA = 6.15%. | The athlete's Family Environment Questionnaire was used. There were 83 questions with 9 scales. It consisted of questions related to the athlete's family environment (e.g., SES, family relationships, sport significance, genetic and environmental activities). Questions were answered on a liker scale 1 = strongly disagree, 5 = strongly agree. | *General Family Functioning (i.e., family relationship)* | N/A | N/A | N/A | N/A | N/A | N/A | N/A | father-adolescent relationship was positively correlated with physical exercise. Father-adolescent relationship and physical exercise only had an indirect effect on internet addiction, mediated by self-esteem and social anxiety. The correlation coefficient between variables: Father-adolescent relationship and physical exercise = 0.040, P<0.01. | General FF: r = 0.040, z' = 0.040 | General FF: z' = 0.040, positive, significant. |
| The effects of parental stress on physical activity among overweight and obese hispanic adolescents: Moderating role of family communication and gender | Kobayashi, Lee, Leite, Esquives, Prado, Messiah, & St. George (2019) | USA | N = 280. Adolescent Female: 52%, Age: 13.01 (0.83), Country of origin: United States 64%, Cuba 19.3%, Honduras 4.3%, Venezuela 3.6%. Parent Female: 88.2%, Age: 44.88 (6.5), Country of origin: United States 8.9%, Cuba 34.3%, Nicaragua 15%, Honduras 11.4%, Marital status: Married 57.9%, Divorced 12.9%, Living with someone 10%, Separated 10%, Never married and not living with someone 8.6%, Widowed 0.7%, Employment: Full-time employment 50.7%, Part-time or temporary employment 22.9%, Homemaker 15.7%, Unemployed 7.1%, Other 3.6%, Annual income: <$30,000 65.4%, >$30,000 20.7%, >$50,000 13.9%, Education: Some or less than high school 18.7%, High school graduate 32.5%, Some college 29.3%, College graduate or more 19.6% | not measured/ disclosed | cross sectional | Secondary analysis used baseline data from an obesity prevention trial involving 280 low-income Hispanic/Latino adolescents (7th/8th grade, BMI ≥ 85th percentile) and their parents in South Florida. Recruitment targeted schools with predominantly Hispanic students near recreational areas. Eligible participants were visually screened for BMI and required parental consent and, if necessary, physician approval for serious health issues. Physical activity was measured using the WHO's Global Physical Activity Questionnaire, summing work, transport, and recreational activities for a total MVPA score. Parent stress was assessed with the abbreviated Hispanic Stress Inventory, creating a composite score from 25 items (Cronbach α = .84). Family communication was measured with the Family Relations Scale, using 3 items (Cronbach α = .92). Descriptive statistics and correlations were analyzed. Multiple linear regressions examined parental stress effects on adolescent MVPA. MVPA was scaled down to avoid convergence issues. Interaction effects of parental stress and family communication were tested, and gender differences analyzed. Demographic covariates were considered, and nonsignificant ones removed. Standardized regression coefficients and effect sizes were reported. Missing data was handled with full-information maximum likelihood. Model fit was evaluated using CFI and TLI. Analyses were done with MPlus. | Secondary data analysis based on baseline data from an obesity prevention efficacy trial. | *Self-report:* PA was measured using WHO's global physical activity questionnaire. This questionnaire was previously used among children and adolescents participating in the National Health and Nutrition Examination survey. This questionnaire assessed the amount of self-reported PA engaged in (used FITT principle). Based on the WHO global PA questionnaire, a summed score of the work in units of minutes for MVPA levels during a typical week was created to capture the total MVPA score. | Adolescent PA (min/wk): 860 (1230) | Family communication was measured using the family relations scale. Parents reported their family communication using 3 items where higher scores indicate more positive communication. Sample question: "my family knows what I mean when I say something." response range: 1 (not true at all) - 3 (true alot). Cronbach α = .92 | *Communication* | N/A | Zero correlations: Family communication was significantly associated with adolescent PA (min/week): r = .171, p < .05 Adjusted models controlling for demographic covariates (Model 2): Beta = .07, p = .39 | N/A | N/A | N/A | N/A | N/A | N/A | COMMUNICATION: r = .171, z' = 0.173. r = .07, z' = 0.07 | Communication: used model 1, model 2 is adjusted: z' = 0.173. signficant, positive. |
| Child-parent reciprocal influences in exercise behavior | Coviak (1998) | USA | N = 121 families, 55 boys, 69 girls. Early adolescents ages: 12 to 15, mid adolescents ages: 15 to 18. M age of fathers = 42.99 (range 32 to 62, SD - 6.39). M age of mothers = 40.30 (range 31 to 54, SD = 4.83). Racial/Ethnic backgrounds of families: African American n = 31, 25.6%. European American n = 81, 66.9%, Hispanic n = 2, 1.7%, Asian n = 2, 1.7%, Arabic n = 1, .8%, unreported n = 4, 3.3%. | Two birth parents n = 69, 57%. mother and stepfather n = 15, 12.4%, mother, unpartnered n = 32, 26.4%. father, unpartnered n = 1, .8%. father and stepmother n = 2. Other family types n = 2, 1.7%. Total number of children living in the homes ranged from 1 to 9 (M = 2.636, SD - 1.372). 90.9% of families reporting 4 or less children. | Descriptive design, and utilized data from two sources - children = longitudinal study, parents = researcher collected data. A pilot study collecting data from parents at two time points, two weeks apart | Pilot Study: the pilot study included adult volunteers recruited from the university medical center and surrounding hospitals of southeastern Michigan. The respondents were parents of at least one child between the ages of 9 and 15 and willing to complete the instruments twice, two weeks apart. Eligible participants were sent out packages in the mail. Once the package was completed and sent back by the participant, a period of 2 weeks was given to then send out a second package (same as the first) for completion. Parent data analysis: To determine instrument reliability scores were entered into SPSS, correlated to estimate reliability, and compared using paired t-tests to check for significant differences. Main study group was drawn from the parents of 341 early and mid-adolescents in grades 7,8,9 and 10 in two southeastern Michigan school districts who had previously participated in a longitudinal study (see Box H). While all adult family members were eligible to participate, questionnaires were only given to one male and one female of the household. Data concerning one of the children was used for analysis as it would be conceivable that parents would respond similarity in their reports about two different children when there were sibling participants in Child/Adolescent Exercise study - coin flip was used to determine which sibling to use. | child participant data drawn from group who had participated in the longitudinal study "Antecedents, Patterns and Health researchers from the University of Michigan School of Nursing and Division of Kinesiology". | Child exercise frequency was measured using the Child/Adolescent Activity Log (CAAL). Validity was tested through correlation of Caltrac accelerometer recordings of one day of exercise with reported exercise from the Log. Correlations in the two of three data collection periods were .51, and .38. Test-retest reliability was estimated from the responses of 25 students who completed the instrument at the beginning and the end of a 45-minute class period over a period of a week. The daily stability coefficients ranged from .73 to .94, and an average summary score attained a correlation of .95. For this study, only children who had four or more days of completed exercise logs for the week of data collection were included in analyses in which exercise frequency is the dependent variable. For each day of the week that the log data was collected, the number of minutes of activity the child reported was counted. These daily exercise totals were summed to obtain a score that reflected the total number of minutes of exercise the child spent in a week. |  | The family flexibility and cohesion was measured through use of FACES II. This instrument was developed by Olson, Portner, and Bell (1982) to test the Circumplex model of Marital and Family Systems. While FACES II was not the most recent form of the instrument, Olson et al. recommended its use of III due to "superior reliability". FACES II = 30 items, 14 to measure flexibility, and 16 to cohesion. There is two versions of the instrument, where the respondent describes the perceived version of their family, and an Ideal version, in which they are asked to complex the questions twice, once to describe their family, and a second time asking how they would like their family to be. Faces II is reported to have an internal consistency coefficient of .9. For the cohesion subscale = .87, and for flexibility = .78. The subscales have been found to have concurrent validity coefficients of .93 (cohesion) and .79 (flexibility) when compared to the global family health measure of the Dallas self report family inventory. | *Cohesion* | Correlations of Model Variables Between Mother and Child (Table 4.13): cohesion and child exercise r = .260 (p < .05). Correlations of Model Variables Between Father and Child: cohesion and child exercise r = -0.087 (p > .05). | N/A | N/A | N/A | N/A | N/A | N/A | N/A | Correlations of Model Variables Between Mother and Child (Table 4.13): cohesion and child exercise r = .260, z' = 0.266. Correlations of Model Variables Between Father and Child: cohesion and child exercise r = -0.087, z' = -0.087 | Cohesion: taken average of mother and father: z' = 0.0895. significant as 50% of p values are signficant. signficant positive. |
